# Co‐Assemblies Regulate the Catalytic Activity of Peptide Fibrils

**DOI:** 10.1002/anie.202511165

**Published:** 2025-12-04

**Authors:** Albin Lahu, Shao‐Lin Wu, Maximilian Schuler, Francesca Mazzotta, Ardit Ramadani, Emirhan Koca, Ingo Lieberwirth, Katharina Landfester, Torsten John, David Y. W. Ng, Tanja Weil

**Affiliations:** ^1^ Max Planck Institute for Polymer Research Ackermannweg 10 D‐55128 Mainz Germany; ^2^ Max Planck School Matter to Life D‐69120 Heidelberg Germany; ^3^ School of Science Constructor University Campus Ring 1 D‐28759 Bremen Germany

**Keywords:** Co‐assembly, Peptide catalysis, Peptides, Retro‐aldolase, Self‐assembly

## Abstract

Short peptide sequences self‐assemble into supramolecular structures through intermolecular interactions, creating a microenvironment in which chemical reactions can be catalyzed. In recent years, many peptide sequences have shown to demonstrate catalytic activity upon nanostructure formation, but the engineering of the catalytic microenvironment through co‐assembly strategies have not been explored. We introduce a peptide sequence that gains retro‐aldolase activity upon assembly to supramolecular peptide fibrils in aqueous buffer solution (pH 7.4). The catalytic activity is first optimized through synthetic sequence variation and the structure formation properties of the peptides are characterized. Co‐assembly with inactive peptide sequences enables the up‐ or downregulation of the catalytic activity over a dynamic range, by modulating the likelihood for substrate interaction and thus the distance of the substrate to the nucleophilic lysine at the active site. It is observed that co‐assemblies with positively charged sequences increase activity, whereas negatively charged peptide sequences decrease activity. We show that the emerging field of peptide‐based catalysts can be further advanced by the engineering of the catalytic domain using heterogeneous supramolecular assembly.

## Graphical abstract

Catalytic self‐assembling peptides (cSAPs) form fibrils that catalyze the retro‐aldol reaction of Methodol. Co‐assembly with inactive peptides tunes catalytic efficiency by altering substrate accessibility and the distance to the nucleophilic lysine. This approach enables precise engineering of catalytic domains and activities in peptide‐based catalytic nanostructures.

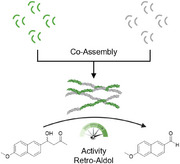

## Introduction

Supramolecular peptide catalysts have been established in both fundamental and application‐driven research due to their numerous advantages, such as facile synthesis and the engineering of supramolecular structures, for instance nanofibers or nanotubes.^[^ ^1^ ^]^ The most common scaffold material for self‐assembly consists of β‐sheet forming peptide amphiphiles, which can be synthesized through alternating sequence patterns of polar and non‐polar amino acids, augmented by an aromatic moiety at the *N*‐terminus.^[^ ^2^, ^3^ ^]^ The development of the next‐generation peptide materials have built upon the existing knowledge to pack higher ordered functionalities, such as catalysis and responsiveness, within a minimalistic scaffold.^[^ ^4^, ^5^ ^]^

Due to their typically short sequences (4–10 residues), catalytic self‐assembling peptides (cSAPs) offer a compact, seamless design to introduce catalytic functions into supramolecular materials. Activities are gained through the formation of nanoscale structures, driven by hydrophobicity, charge, and size of the peptide monomers.^[^ ^6^, ^7^ ^]^ These supramolecular structures in turn create microenvironments through the interactions between functional groups of amino acid side chains. Chemical parameters, such as p*K*_a_‐values, nucleophilicities and reaction centers, can be drastically modified within these microenvironments akin to those found in enzyme active sites.

Beyond β‐sheets, other scaffolds such as β‐turns or α‐helices can be tailored to provide the necessary environment for reaction catalysis.^[^ ^4^, ^8^ ^]^ For instance, helical bundles formed by helix‐loop‐helix motifs have been used to mediate hydrolysis of esters through the formation of an active center with two histidine and four arginine side chains, while β‐sheets have been proven to be effective scaffolds in aldol reactions with proline and lysine in the active sites.^[^ ^9^, ^10^, ^11^ ^]^ In addition to secondary structural motifs, nanostructure morphologies, such as nanofibers, nanotubes, and micelles also influence catalytic efficiencies. Nanofibers and nanotubes have been reported to be advantageous due to high densities of catalytic sites imparted by their internal order.^[^ ^12^, ^13^, ^14^ ^]^ Since the inception of cSAPs, their catalytic portfolio has expanded by using different amino acids within the active site (His for hydrolysis^[^ ^15^ ^]^ and oxidation,^[^ ^16^ ^]^ Pro for *C*–*C*‐bond formation,^[^ ^17^ ^]^ and Lys for *C*–*C*‐bond cleavage).^[^ ^18^ ^]^

Among these reactions, the retro‐aldol reaction is particularly significant due to its essential role in metabolism as well as synthetic chemistry.^[^ ^19^, ^20^, ^21^ ^]^ The conversion of 4‐hydroxy‐4‐(6‐methoxy‐2‐naphthyl)‐2‐butanone (Methodol **1**) is used as a standard to evaluate retro‐aldolase activity due to the fluorogenic properties of its product, 6‐methoxy‐2‐naphthylaldehyde **2**.^[^ ^22^ ^]^ In aqueous media, retro‐aldol reactions can be catalyzed through addition of acids or bases. To perform the reaction at neutral pH, enzymes use the ε‐NH_2_‐group of Lys to conduct a nucleophilic attack on the carbonyl‐group of the substrate, followed by the formation of an enamine. The p*K*_a_‐value of the ε‐NH_2_ of Lys is 10.3 and has to be lowered in order to enable this capability, for instance, by subjecting the functional group within a less polar microenvironment.^[^ ^23^, ^24^ ^]^ Nonetheless, the *C*–*C*‐bond remains a major challenge for efficient catalysis due to its stability.^[^ ^25^ ^]^

Within known peptide sequences exhibiting retro‐aldolase function, it was postulated that the active Lys moiety has to be situated at either terminus of a β‐sheet forming peptide monomer.^[^ ^18^, ^26^ ^]^ We hypothesized that an internal Lys, flanked by hydrophobic amino acids, can create a similar catalytic environment and thus alleviate the positional limitation.

**Figure 1:**
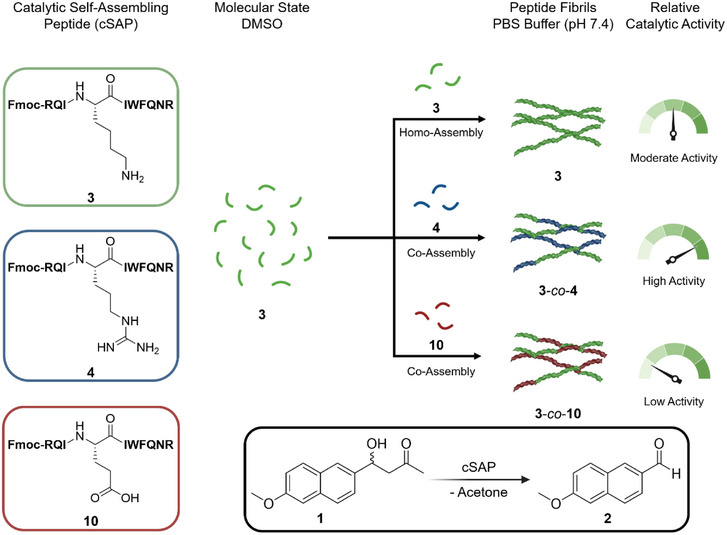
Simplified chemical structure of cSAP's Fmoc‐RQIKIWFQNR **3**, Fmoc‐RQIRIWFQNR **4**, and Fmoc‐RQIEIWFQNR **10** with a graphical depiction showing the formation of homo‐ and co‐assembled cSAP fibrils, starting from the molecular state. The depiction includes the relative catalytic activities of the fibrils regarding the retro‐aldol reaction of Methodol **1** to 6‐methoxy‐2‐naphthaldehyde **2**.

Herein, we designed a cSAP, Fmoc‐RQIKIWFQNR **3**, containing an internal Lys, to catalyze the retro‐aldol reaction of Methodol **1** (Figure 1). The internal Lys moiety in Fmoc‐RQIKIWFQNR **3** is flanked by two Ile amino acids, creating a hydrophobic microenvironment that is expected to be catalytically active under neutral, aqueous conditions. Several peptide variants are tested regarding their assembling properties and activity to further elucidate the activity of the sequence, inspired by biological site‐directed mutagenesis principles.^[^ ^27^ ^]^ Furthermore, the impact of the aromatic π‐block on assembly and activity will be investigated as it confers amphiphilic character onto the peptide sequence.^[^ ^28^, ^29^ ^]^

Although the control over catalytic activity can be obtained through implementation of stimulus‐responsive groups,^[^ ^30^, ^31^, ^32^ ^]^ we believe that their chemical space and catalytic efficiencies could achieve further diversification through co‐assembly. As mixtures of monomers interact, they access new structural portfolios^[^ ^33^ ^]^ with additive features, such as new mechanical, optical and chemical functionalities.^[^ ^34^, ^35^ ^]^ As such, we summarized that co‐assembly strategies could be used to dynamically engineer the catalytic domain in active peptide assemblies. Due to the sensitivity of the catalytic site, we expect that the catalytic activity will be augmented or suppressed by charged co‐monomers that increase or decrease, respectively, the average minimum distance of the substrate and Lys. By showing how simple co‐assembly technology expands the activity range of cSAPs and the potential chemical space that can be offered, we hope to inspire further advancements of cSAPs in complex and sustainable catalytic systems.

## Results and Discussion

### Synthesis and Characterization of cSAP's

**Figure 2:**
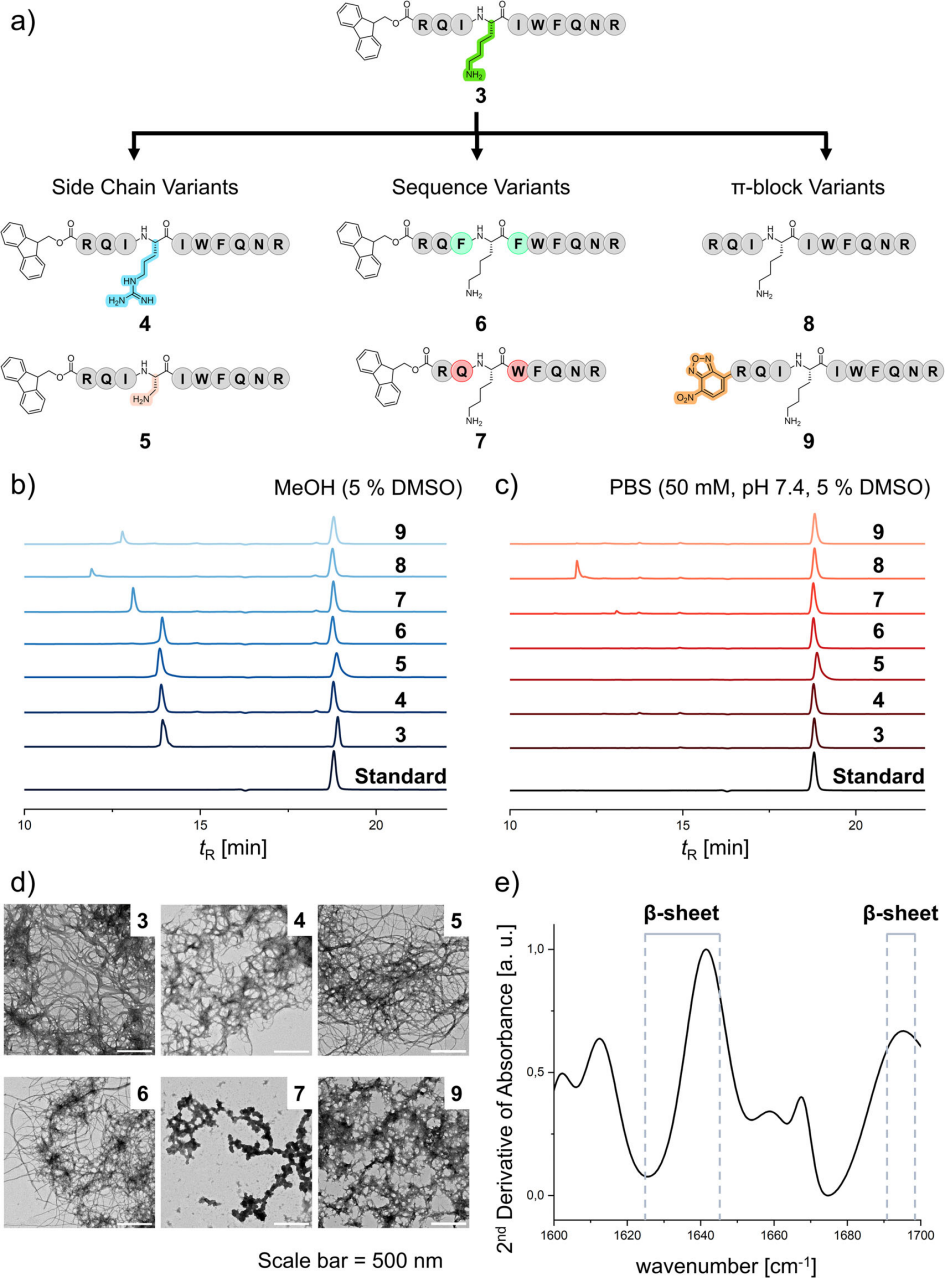
a) Schematic depiction of the cSAPs and the structural changes compared to the lead peptide Fmoc‐RQIKIWFQNR **3**. b) Elugrams of Fmoc‐RQIKIWFQNR **3**, Fmoc‐RQIRIWFQNR **4**, Fmoc‐RQI*Dap*IWFQNR **5**, Fmoc‐RQFKFWFQNR **6**, Fmoc‐RQKWFQNR **7**, RQIKIWFQNR **8**, and NBD‐RQIKIWFQNR **9** (all 100 µm) in MeOH (5% DMSO) as well as the internal standard Fmoc‐Trp(Boc)‐OH (100 µM in MeOH, **Standard**). Elugrams are shown as a plot of absorbance intensity at 214 nm against retention time *t*_R_ and referenced to the standard. c) Elugrams of Fmoc‐RQIKIWFQNR **3**, Fmoc‐RQIRIWFQNR **4**, Fmoc‐RQI*Dap*IWFQNR **5**, Fmoc‐RQFKFWFQNR **6**, Fmoc‐RQKWFQNR **7**, RQIKIWFQNR **8** and NBD‐RQIKIWFQNR **9** (all 100 µM) in PBS (50 mM, pH 7.4, 5% DMSO) as well as the internal standard Fmoc‐Trp(Boc)‐OH (100 µM in MeOH, **Standard**). Elugrams are shown as a plot of absorbance intensity at 214 nm against retention time *t*_R_ and referenced to the standard. d) TEM micrographs of nanostructures formed by Fmoc‐RQIKIWFQNR **3**, Fmoc‐RQIRIWFQNR **4**, Fmoc‐RQI*Dap*IWFQNR **5**, Fmoc‐RQFKFWFQNR 6, Fmoc‐RQKWFQNR **7**, and NBD‐RQIKIWFQNR **9** (all 100 µm) in PBS (50 mm, pH 7.4, 5% DMSO), stained with uranyl acetate (4%), scale bar = 500 nm. e) FTIR‐spectrum of Fmoc‐RQIKIWFQNR **3** (1 mm) in PBS (50 mm, pH 7.4, 5% DMSO) in the range of 1600 – 1700 cm^−1^. The 2^nd^ derivative of absorbance is plotted against the wavenumber.

The substrate Methodol **1** was synthesized and characterized according to literature (Figures S1–S4).^[^ ^36^ ^]^ Next, we synthesized the cSAP Fmoc‐RQIKIWFQNR **3**. The sequence was chosen to contain Lys, since natural Type I Aldolases require Lys‐side chains in their active center to catalyze the *C*–*C*‐bond degradation in its substrate.^[^ ^37^ ^]^ To further understand the importance of Lys in the catalyzed reaction, a Lys→Arg‐variant, Fmoc‐RQI **R** IWFQNR **4**, of the original sequence was designed. This sequence maintains the positive charge of the side chains, while changing the amino‐group to a less nucleophilic guanidino‐group. To analyze the impact of the side chain accessibility on catalytic activity, we synthesized the variant Fmoc‐RQI *Dap* IWFQNR **5**, thus shortening the side chain length from four to one methylene‐unit and limiting access to the amino‐group. Furthermore, changes close to the catalytic center have been reported previously to interfere with the catalytic pocket, causing changes in activity and stability of enzymes.^[^ ^38^, ^39^ ^]^ Therefore, we synthesized peptide variants with alternative side chains in close proximity to the active Lys‐side chain, namely Fmoc‐RQ **FKF** WFQNR **6** and Fmoc‐R **QKW** FQNR **7**, to analyze their effects on the self‐assembly and catalytic behavior of the peptides. Finally, the impact of the *N*‐terminal π‐block was analyzed with the absence of the π‐group in native RQIKIWFQNR **8** as a control and by swapping the Fmoc‐group with a smaller nitrobenzofurazan (NBD)‐group in NBD‐RQIKIWFQNR **9**. The variations are summarized graphically in Figure 2a.

Peptide synthesis was performed on *Wang*‐resin using Fmoc solid‐phase synthesis with *N,N*’‐diisopropylcarbodiimide (DIC) and ethyl (2*Z*)‐2‐cyano‐2‐(hydroxyimino)acetate (Oxyma Pure) for the coupling steps. The peptides were purified via high performance liquid chromatography (HPLC) and subsequently characterized using liquid chromatography‐mass spectrometry (LC‐MS), matrix‐assisted laser desorption/ionization (MALDI) and high‐resolution electrospray ionization (High‐Res ESI) revealing high purities (>95%) and sufficient yields (20% – 50%) of the cSAPs (Figures S5–S11).

The self‐assembly behavior of the cSAPs was first analyzed using analytical HPLC. The assembly concentration of the peptide monomers in the chosen aqueous buffer system was determined by preparing high concentration stock solutions in DMSO (10 mm) followed by dilution into PBS buffer (50 mm, pH 7.4) affording final cSAP concentrations of 100, 75, 50, and 25 µm with 5% DMSO. The diluted solutions were incubated for 24 h at room temperature to allow for self‐assembly. Identical preparations in methanol (5% DMSO) were used as non‐assembled, molecular references. After incubation, the samples were filtered (0.2 µm) to remove any aggregates or assembled supramolecular structures in solution and Fmoc‐Trp(Boc)‐OH (100 µm in MeOH, **Standard**) was added to the filtrate as an internal standard. In MeOH, where no assembly occurs, the signals of the analyzed peptides occur between 12 and 15 min (Figure 2b). In contrast, no peptide monomer signal could be detected in the PBS‐incubated samples for peptides **3**, **4**, **5**, **6**, **7**, and **9** (Figure 2c). Analysis at lower concentrations indicate that peptides **3** – **7** and **9** already assemble at the lowest tested concentrations of 25 µm (Figures S17–S19). Only the native sequence **8** does not assemble in PBS at the tested concentrations (Figures 2c and S17–S19).

Based on these results, the critical assembly concentrations are above 100 µm for RQIKIWFQNR **8** and below 25 µm for the other cSAPs, indicating that aromatic π‐blocks are necessary for assembly at the tested concentrations and conditions. These analyses were further used to determine the assembly conversion rates (*CR*) of the cSAPs at 100 µm concentrations. The area under the curve was calculated for the respective peptide signals in MeOH and PBS buffer. Through comparison of the signals, the conversion of each peptide was obtained. For peptides **3** – **7** as well as **9** complete assembly conversion was observed (*CR* > 95%), whereas no conversion was detected for the native sequence **8**. The results were further confirmed by Proteostat^®^ Aggregation Assay at 100 µm peptide concentrations. An increase in Proteostat^®^ fluorescence was observed for cSAPs **3** – **7**, whereas for the native sequence **8**, the fluorescence does not increase due to the absence of supramolecular structures (Figure S22).

The formed supramolecular assemblies were further analyzed using Fourier‐transform infrared (FTIR) spectroscopy. Secondary structures within peptide assemblies can be identified through FTIR‐analysis of the amide I bond frequencies, which characterize the *C*═*O* stretching vibration and are minimally affected by the nature of neighboring side chains.^[^ ^40^, ^41^, ^42^ ^]^ The FTIR‐analyses in PBS (50 mm, pH 7.4, 5% DMSO) after 24 h incubation at room temperature revealed strong bands at 1640 cm^−1^ for the cSAPs, correlating to β‐sheet secondary structures (Figures 2e and S24) after subtraction of the PBS spectrum (Figure S23).^[^ ^40^ ^]^ Weaker bands are observed in the random coil region around 1648 cm^−1^ and in the β‐turn region at 1668 cm^−1^, indicating the presence of these structures to a smaller extent. For Fmoc‐RQKWFQNR **7** and RQIKIWFQNR **8**, the β‐sheet band is also observed, however the intensity of this band is almost equal to that of the random coil band, indicating that both secondary structures are present at approximate equal ratios (Figure S24). Therefore, FTIR‐analyses imply that hydrophobic amino acids in position three and five of the peptide sequence, as well as a hydrophobic π‐block at the *N*‐terminus, favor the formation of β‐sheets, most likely due to their hydrophobic interactions and π–π‐stacking respectively. Circular Dichroism (CD) spectroscopy measurements indicate the presence of antiparallel β‐sheets for cSAP **3** (Figure S27), characterized by a positive band at 195 nm followed by a negative band at 220 nm,^[^ ^43^ ^]^ after changing of the co‐solvent DMSO to 2,2,2‐trifluoroethanol (TFE) due to the interfering absorption of DMSO in the secondary structure range.

Using transmission electron microscopy (TEM), the nanostructures and morphologies of the synthesized cSAPs were characterized. Here, fibrillar structures were observed for cSAPs **3** – **6** and **9** (Figure 2d). Peptide **7** exhibited amorphous aggregates (Figure 2d), whereas for RQIKIWFQNR **8**, no structures were observed (Figure S28). The TEM study supports the results of the analytical HPLC‐study, suggesting that a π‐block is required for structure formation at the tested concentrations. Furthermore, we observed that hydrophobic amino acids in position three and five of the peptide sequence are required for the formation of fibrillar structures, whereas deletions in this region lead to the formation of amorphous aggregates. The formation of fibrillar structures was also confirmed using cryogenic electron microscopy (cryo‐EM) for cSAP Fmoc‐RQIKIWFQNR **3** at 100 µm concentration (Figure S29).

### cSAP Homo‐Assemblies Mediate Catalysis of the Retro‐Aldol Reaction

**Figure 3:**
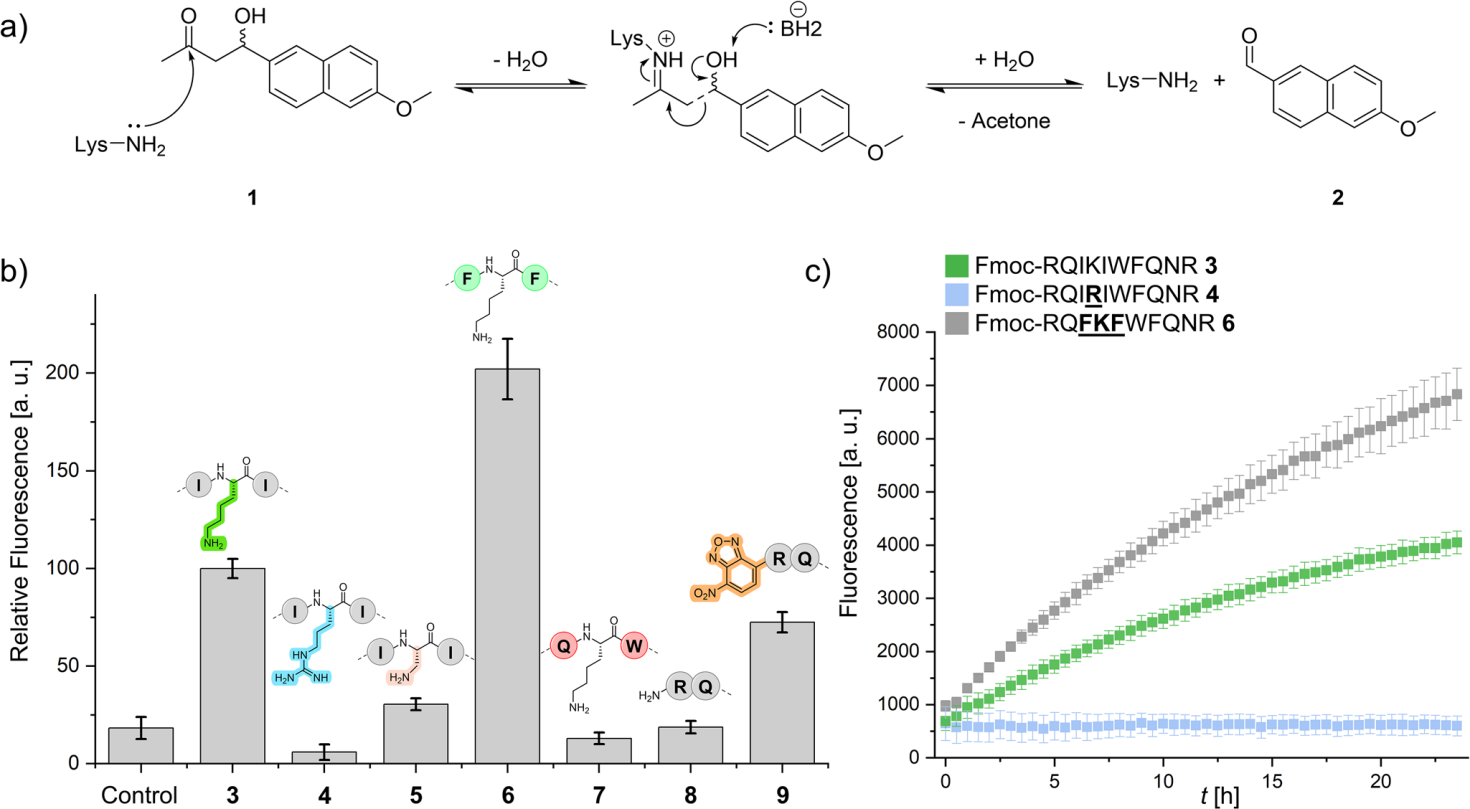
a) Chemical equation of the peptide‐catalyzed retro‐aldol reaction of Methodol **1** to 6‐methoxy‐2‐naphthaldehyde **2**. b) Fluorescence intensity bar chart of Fmoc‐RQIKIWFQNR **3**, Fmoc‐RQIRIWFQNR **4**, Fmoc‐RQI*Dap*IWFQNR **5**, Fmoc‐RQFKFWFQNR **6**, Fmoc‐RQKWFQNR **7**, RQIKIWFQNR **8**, and NBD‐RQIKIWFQNR **9** (all 100 µm) at *λ*_ex_ = 330 nm and *λ*_em_ = 452 nm after 24 h incubation with Methodol **1**. Control shows Methodol **1** (50 µm) in PBS (50 mm, pH 7.4, 5% DMSO) without peptide. c) Kinetic analysis of 6‐methoxy‐2‐naphthaldehyde **2** formation from Methodol **1** catalyzed by cSAPs **3**, **4**, and **6** at 100 µm in PBS (50 mm, pH 7.4, 5% DMSO) over 24 h. The fluorescence intensity at *λ*_ex_ = 330 nm and *λ*_em_ = 452 nm is plotted against time *t* with 30 min time points over 24 h.

The activities of the cSAPs on the retro‐aldol reaction were tested with the degradation of Methodol **1**. The mechanism of the retro‐aldol reaction requires an amine‐group which performs a nucleophilic attack on the carbonyl atom of Methodol **1**, subsequently forming an enamine (Figure 3a), followed by the release of 6‐methoxy‐2‐naphthaldehyde **2** and acetone. The complete mechanism is thoroughly reported in previous literature.^[^ ^44^, ^45^, ^46^ ^]^ The differences in fluorescence characteristics between substrate **1** and product **2** (Figure S33A) enable the detection of catalytic turnover (*λ*_ex_ = 330 nm, *λ*_em_ = 452 nm). Fluorescence calibration of 6‐methoxy‐2‐naphthaldehyde **2** showed a linear increase for concentrations from 1 – 60 µm in the chosen PBS buffer (50 mm, pH 7.4, 5% DMSO; Figure S33B). Hence, a working concentration of 50 µm Methodol **1** was chosen as a standard for our analysis of catalytic activities.

The analyzed cSAPs (**3** – **9**, 100 µm) were incubated for 24 h in PBS (50 mm, pH 7.4, 5% DMSO) at room temperature to allow pre‐assembly of supramolecular structures for the self‐assembling peptides **3** – **7** and **9**. To these pre‐assembled structures, Methodol **1** (50 µm) and 6‐methoxy‐2‐naphthaldehyde **2** (5 µm) were added. Here, aldehyde **2** was used to establish a baseline fluorescence and the mixtures were incubated for another 24 h at 37 °C in the dark. Background subtraction was performed using samples containing peptide (100 µm) but without substrate **1**. Control samples without peptide, solely containing Methodol **1** and 6‐methoxy‐2‐naphthaldehyde **2** were also prepared to monitor the stability of substrate **1** under the chosen buffer conditions. The activities of the cSAPs **3** – **9** were tested under these conditions (Figure 3b).

The negative control shows no increase in fluorescence, supporting that Methodol **1** is stable under the buffer conditions. Catalytic activity is detected for Fmoc‐RQIKIWFQNR **3**, Fmoc‐RQFKFWFQNR **6**, and NBD‐RQIKIWFQNR **9**. The Lys→Arg‐variant, compound **4**, does not show any activity. The similarities to the active cSAP **3** in aggregation concentration (Figure 2b,c), secondary structure composition (Figure S24), and fibrillar morphology observed in TEM (Figure 2d) indicate that the loss of activity is caused by the missing amino‐group in the arginine side chain, which is required for the retro‐aldol reaction. Activity loss is also observed for the Fmoc‐RQI*Dap*IWFQNR **5** variant, which showed no differences in the assembly properties compared to cSAP **3** (Figures 2b–d and S24), indicating that a longer side chain is required to provide sufficient access of the nucleophilic amino‐group to substrate **1**. Furthermore, it is observed that cSAP's Fmoc‐RQKWFQNR **7** and RQIKIWFQNR **8** do not show any activity (Figure 3b). The deletion of hydrophobic amino acids in proximity to the active Lys‐side chain in **7** resulted in less β‐sheet content observed in FTIR (Figure S24) and showed amorphous aggregates in TEM (Figure 2d). Therefore, it is expected that catalytic activity is caused by supramolecular structures with a defined arrangement of Lys‐side chains, which is necessary to activate the amino‐group to establish catalytic conversion of Methodol **1**. The requirement of defined supramolecular structures is further supported by the inactiveness of RQIKIWFQNR **8**, which does not assemble under the buffer conditions as demonstrated by HPLC (Figure 2b,c), Proteostat^®^ Aggregation Assay (Figure S22) and TEM (Figure S28). Compared to cSAP **3**, a decrease in activity is observed for NBD‐RQIKIWFQNR **9**. Since analyses of the assembly characteristics exhibit similar conversion rates (analytical HPLC, Figure 2b,c), secondary structures (FTIR, Figure S24) and morphologies (TEM, Figure 2d), the decrease in activity is likely caused by the weaker π–π‐interactions of the NBD‐group.

In contrast, the Ile→Phe variant **6** in close proximity to the active Lys‐side chain shows increased catalytic activity of the fibrillar structures compared to those formed by cSAP **3** (Figure 3b). Increased π–π‐interactions of the Phe‐residues, which are stronger than the hydrophobic interaction of the Ile‐residues, cause the formation of more stable fibrils,^[^ ^47^ ^]^ which seem to be beneficial for the catalytic activity of the cSAP.

Kinetic measurements with varying Methodol **1** concentrations were performed to obtain the Michaelis–Menten constants *K_M_* and reaction rates *v_max_* of cSAPs **3** and **6** (Figure S37). The analysis revealed *K_M_*‐values of (253.5 ± 10.2) µm for Fmoc‐RQIKIWFQNR **3** and (208.0 ± 8.7) µm for the more active Fmoc‐RQFKFWFQNR **6** peptide variant. Therefore, these results further indicate that stable Phe‐fibrils allow for better substrate‐binding compared to the Ile‐containing fibrils. Additionally, when looking into the *v_max_*‐values we observe that the fastest reaction rate for cSAP **3** is (10.2 ± 0.5) nm min^−1^, whereas for variant **6** the *v_max_* is at (18.4 ± 0.9) nm min^−1^. From these kinetics, it is likely that the increased activity of cSAP **6** is caused by both, a better substrate binding and thus a more efficient active site. The *K_M_* and *v_max_*‐values of the active cSAPs are summarized in Table S2.

Kinetics of the variants Fmoc‐RQIKIWFQNR **3**, Fmoc‐RQIRIWFQNR **4**, and Fmoc‐RQFKFWFQNR **6** over 24 h at 50 µm Methodol **1** concentrations were also performed and used to determine initial rates *v_i_* of the catalyzed reaction (Figure 3c). Since the conversion rates are similar for the cSAPs **3** – **7** as well as **9**, showing that no peptide monomers are present, the availability and accessibility of deprotonated Lys amino‐groups is expected to be the main factor for differences in initial rates. For Fmoc‐RQIKIWFQNR **3**, *v_i_* has been determined to be at (1.59 ± 0.07) nm min^−1^ through linear fit of the first 10 measurement points and subsequent calculation using a product **2** calibration curve (Figure S33). For Fmoc‐RQFKFWFQNR **6** an initial rate of (3.34 ± 0.16) nm min^−1^ was observed, revealing that the *v_i_* of peptide **6** increases by (110 ± 0.06)% compared to cSAP **3**. For the control and Fmoc‐RQIRIWFQNR **4**, the initial rates are lower than 1% compared to **3**. The differences in initial rates further emphasize the improved catalytic activity of cSAP **6** compared to cSAP **3** through variation of the hydrophobic amino acids. When comparing the kinetic values to natural aldolases, it is interesting that the *K_M_* value of compound **3** is in a similar range as the *Ignicoccus hospitalis* fructose‐1,6‐bisphosphate aldolase/phosphatase.^[^ ^48^ ^]^ The reaction rates however, cannot be compared directly, as the exact number of active sites present in the peptide assemblies cannot be accurately known. Fmoc‐RQIKIWFQNR **3** exhibits a turnover number *k_cat_* of (1.02 ± 0.01) · 10^−4^ min^−1^, which places it between synthetic, nanotubular, and foldamer retro‐aldolases.^[^ ^18^, ^26^, ^49^ ^]^

To further analyze the substrate‐fibril interactions, molecular dynamics (MD) simulations were performed. First, *AlphaFold* 3 (AF3), which is based on deep learning,^[^ ^50^ ^]^ was used to predict arrangements of peptide fibrils consisting of 20 monomers (without Fmoc, Figure S41), since this approach has previously been shown to be a suitable way to achieve models of peptide oligomers and fibrils.^[^ ^51^ ^]^ MD simulations of the AF3 models, extended by the Fmoc‐modification, were performed to obtain a representative structure of the peptide fibrils (Figure S42). The fibril models were then used and five Methodol **1** molecules added to determine the most dominant arrangements for each system using clustering analysis (Figures S43–S45). Details on the simulation times and size of each cluster are summarized in Tables S1 and S4 respectively.

For cSAP **3**, Methodol **1** was mostly located close to the Lys residues, intercalated into the fibril dry interface, or in small aggregates at the fibril ends. Similar arrangements were observed for cSAP **4** and the Arg residues. For Fmoc‐RQFKFWFQNR **6**, a more crowded and hydrophobic inside of the fibril is observed. Substrate **1** was mostly close to the Lys residues, or at the fibril ends, which is also observed in lower average minimum distances between Methodol **1** and Lys in cSAP **6**, which were determined to obtain quantitative insight into the entire simulation trajectories. Therefore, the MD results indicate that the presence of phenylalanine inside of the fibrils in variant **6** increases the average proximity, and frequency, between Methodol **1** and the active lysine amino‐group of cSAP **6** compared to Fmoc‐RQIKIWFQNR **3**.

Next, the effect of Fmoc‐RQIKIWFQNR **3** concentration on catalytic activity was investigated. For this, the activity of cSAP **3** was tested at various peptide concentrations (500 – 5 µm) and constant Methodol **1** concentrations (50 µm, Figure S34). The analysis revealed an increase of activity with higher concentrations of **3**. At concentrations below 25 µm, no increase in fluorescence compared to the control is detected, correlating to the critical assembly concentration obtained from analytical HPLC (Figure S19).

To further analyze the stability of catalyst **3**, we assembled fibrillar structures and matured them for seven days at room temperature. Subsequent activity analysis, compared to fibrillar structures used after 24 h assembly, revealed no differences in activity. Furthermore, TEM analysis of the matured cSAP assembly showed no observable morphological differences compared to the 24 h sample (Figure S35). The catalytic activity of cSAP **3** is even maintained after ultra‐sonication of the fibrillar structures for 5 min at room temperature. TEM analysis of ultra‐sonicated Fmoc‐RQIKIWFQNR **3** assemblies show fibrillar structures with less entanglements compared to conventionally incubated assemblies of cSAP **3**, indicating the disassembly of super‐agglomerates through agitation (Figure S36). These results underline the robustness of the peptide catalyst over time and under harsh ultra‐sonication conditions, thus providing the possibility to use the catalytic fibrils under broader environmental conditions compared to enzymes.

### cSAP Co‐Assemblies Allow Regulation of Catalytic Activity

The activity of cSAPs is based on a microenvironment established through the arrangement of amino acid side chains in the fibrils. For instance, the density of Lys‐moieties and potential shielding by acidic side chains has an impact on catalytic activity. We added the sequences Fmoc‐RQIEIWFQNR **10**, Fmoc‐RQISIWFQNR **11**, Fmoc‐RQILIWFQNR **12**, and Fmoc‐RQIHIWFQNR **13** to analyze the impact of cSAP **3** co‐assemblies with positively charged side chains (**4**), negatively charged side chains (**10**), and neutral side chains (**9**, **12**, **13**) on activities. Peptides **10** – **13** were synthesized similarly to the previously introduced cSAP's in high purities (>95%) and sufficient yields (10% – 22%, Figures S12–S15) using Fmoc‐solid phase synthesis. Analyses of the self‐assembly behavior in PBS buffer (50 mm, pH 7.4, 5% DMSO) revealed complete assembly conversion at 100 µm using HPLC (Figure S21) and fibrillar structures using TEM (Figure S28) for cSAPs **10** – **13**.

Next, we prepared peptide co‐assemblies of Fmoc‐RQIKIWFQNR **3** separately with **4**, **10**, **11**, **12** and **13** respectively at 1:1 ratio. The co‐assemblies were prepared in the monomeric state within the DMSO stocks to ensure mixing of cSAP monomers prior to the assembly process. For all analyses at the chosen ratio, we kept the concentration of peptide **3** constant at 100 µm.

**Figure 4:**
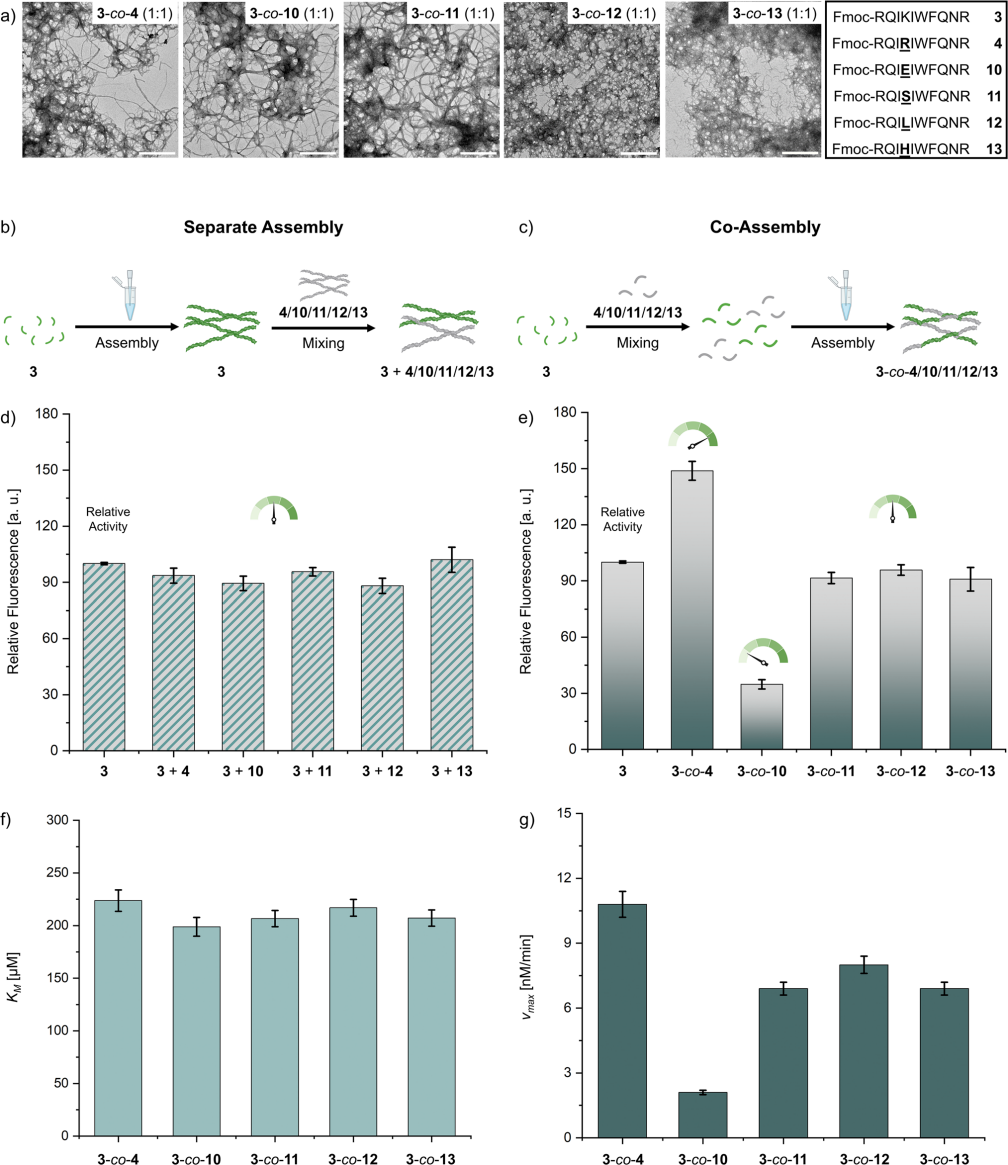
a) TEM micrographs of nanofibers formed by **3**‐*co*‐**4**, **3**‐*co*‐**10**, **3**‐*co*‐**11**, **3**‐*co*‐**12** and **3**‐*co*‐**13** (1:1, 100 µm each peptide) in PBS (50 mm, pH 7.4, 5% DMSO), stained with uranyl acetate (4%), scale bar = 500 nm. b) Schematic depiction for the preparation of separately pre‐assembled mixtures. c) Schematic depiction for the preparation of co‐assemblies. d) Fluorescence intensity bar chart of separately pre‐assembled peptide mixtures **3** + **4**/**10**/**11**/**12**/**13** (1:1; 100 µm each peptide) and homo‐cSAP Fmoc‐RQIKIWFQNR **3** (100 µm) at *λ*_ex_ = 330 nm and *λ*_ex_ = 452 nm after 24 h incubation with Methodol **1** in PBS (50 mm, pH 7.4, 5% DMSO). e) Fluorescence intensity bar chart of **3**‐*co*‐**4**/**10**/**11**/**12**/**13** (1:1; 100 µm each peptide) and homo‐cSAP Fmoc‐RQIKIWFQNR **3** (100 µm) at *λ*_ex_ = 330 nm and *λ*_ex_ = 452 nm after 24 h incubation with Methodol **1** in PBS (50 mm, pH 7.4, 5% DMSO). f) Bar chart containing the Michaelis‐constants *K*_M_ of **3**‐*co*‐**4**, **3**‐*co*‐**10**, **3**‐*co*‐**11**, **3**‐*co*‐**12** and **3**‐*co*‐**13** (1:1, 100 µm each peptide) in PBS (50 mm, pH 7.4, 5% DMSO). g) Bar chart containing the maximal reaction rates *v*_max_ of **3**‐*co*‐**4**, **3**‐*co*‐**10**, **3**‐*co*‐**11**, **3**‐*co*‐**12** and **3**‐*co*‐**13** (1:1, 100 µm each peptide) in PBS (50 mm, pH 7.4, 5% DMSO).

The assembly characteristics of the co‐assemblies were analyzed similarly to the homo‐assemblies presented previously. The assembly rate of the co‐assemblies was tested at 1:1 ratio, with 100 µm **3** and 100 µm **4**/**10**/**11**/**12**/**13** (**3**‐*co*‐**4**/**10**/**11**/**12**/**13**). The results revealed complete assembly (>95%) for all tested cSAP co‐assemblies (Figure S20) and indicate that no soluble peptide monomers are present at the chosen conditions. FTIR‐measurements were performed to analyze the secondary structures of the co‐assemblies and revealed β‐sheet structures for all co‐assemblies, with similar bands as for the homo‐assemblies of **3**, **10**, **11**, **12** and **13** (Figures S25 and S26). Besides β‐sheet structures, a band with less intensity in the random coil region is observed. Between co‐assemblies, no major differences are observed in the FTIR analyses. Subsequent TEM‐analysis of the cSAP co‐assemblies at 1:1 ratio revealed fibrillar structures for all cSAP combinations (Figure 4a). The morphologies of the formed structures resemble those observed for homo‐assemblies **3**, **4**, **10**, **11**, **12** and **13** (Figures 2d and S28). Cryo‐EM measurement of **3**‐*co*‐**4** (1:1) further confirmed fibrillar structures at the chosen buffer conditions and concentrations (Figure S30).

Self‐sorting of assembling peptide monomers describes the preference of monomers to form homo‐assemblies based on molecular recognition in form of hydrogen bonding and other intermolecular interactions. Various self‐sorting, peptide‐based supramolecular structures have been reported in the literature.^[^ ^52^, ^53^ ^]^ We analyzed the assembly properties of the cSAPs Fmoc‐RQIKWFQNR **3** and Fmoc‐RQIRIWFQNR **4** as well as the co‐assembly **3**‐*co*‐**4** (1:1) using the Nile Red Assay. Interactions of Nile Red with peptide assemblies are expected to increase fluorescence due to binding of the dye to hydrophobic microenvironments.^[^ ^54^ ^]^ Concentrations of the homo‐cSAPs were chosen to be below the critical assembly concentration (10 µm), where the co‐assembly **3**‐*co*‐**4** contains 10 µm peptide concentration each (1:1). Individually, cSAPs **3** and **4** reveal low fluorescence intensities, and therefore no assembly (Figure S31). For co‐assembly **3**‐*co*‐**4** however, a multiplicative increase in fluorescence is observed, demonstrating the cooperative interactions between them. TEM‐analysis at the respective concentrations revealed fibrillar structures for the co‐assembly, whereas the homo‐cSAPs showed no supramolecular structures at 10 µm (Figure S31).

Co‐assembly was further characterized using Förster Resonance Energy Transfer (FRET)^[^ ^55^ ^]^ with fluorophore NBD as the donor and 5‐(and 6)‐carboxytetramethylrhodamine (TAMRA) as the acceptor.^[^ ^56^ ^]^ TAMRA‐RQIKIWFQNR **14** was synthesized using Fmoc‐solid phase synthesis in high purities (>95%) and characterized similarly to the previously introduced peptides (Figure S16). Next, we analyzed the fluorescence intensities of NBD‐RQIKIWFQNR **9**, TAMRA‐RQIKIWFQNR **14** (both at 50 µm), and **9**‐*co*‐**14** (1:1, 50 µm each peptide) at *λ*_ex_ = 464 nm and *λ*_em_ = 500 – 700 nm. The results indicate the presence of co‐assemblies through donor‐quenching and an increase in acceptor fluorescence through FRET (Figure S32). Additionally, we tested cSAP mixtures of separately pre‐assembled fibrils, which were then combined, to act as controls, should the peptides exhibit self‐sorting behavior (Figure 4b,d). The pre‐assembled structures were mixed prior to addition of Methodol **1** and then analyzed according to the general procedure.

Catalysis measurements of the cSAP co‐assemblies were performed similar to the procedure described previously, through preparation of co‐assemblies in high concentrations in DMSO (20 mm) and incubation for 24 h in PBS (50 mm, pH 7.4, 5% DMSO) at room temperature to ensure pre‐assembly of the fibrillar structures (Figure 4c). For comparison of activities, cSAP's **3**, **4**, **10**, **11**, **12** and **13** were prepared at 100 µm and incubated in the same way (Figure S40).

For separate assembly and subsequent mixing (Figure 4b,d), no difference in catalytic activity is observed, indicating that the enhancement in activity is caused by co‐assembly and not by the additive presence of both peptide assemblies. These findings also suggest that the co‐assembled systems are not self‐sorting, since the catalytic activity of a self‐sorted system is expected to not change compared to the separately mixed peptide assembly. The homo‐cSAP's Fmoc‐RQIRIWFQNR **4**, Fmoc‐RQIEIWFQNR **10**, Fmoc‐RQISIWFQNR **11**, Fmoc‐RQILIWFQNR **12** and Fmoc‐RQIHIWFQNR **13** do not show any activity at the tested conditions (Figure S40).

The results obtained for the co‐assemblies, however, showed an enhancement of catalytic activity for **3**‐*co*‐**4** (1:1, 100 µm each peptide) compared to the cSAP **3** homo‐assembly (Figure 4c,e). Kinetic experiments revealed the *K_M_*‐value of **3**‐*co*‐**4** to be at (223.7 ± 10.2) µm, indicating an improved substrate affinity of (22 ± 5)% (Figures 4f and S38). Furthermore, an increase of the reaction rate *v*_max_ to (10.8 ± 0.6) nm min^−1^ is observed (Figure 4g) compared to the homo‐cSAP **3**. Increased activities were also observed for various concentrations of **3**‐*co*‐**4** (500 – 5 µm, Figure S34) compared to homo‐assembly **3**.

For **3**‐*co*‐**10** (1:1, 100 µm each) a decrease in activity was observed (Figure 4e). Even though the *K_M_*‐value is below the one for cSAP **3**, the *v_max_* was reduced by (79 ± 1)% for **3**‐*co*‐**10** (1:1). The other tested co‐assemblies, **3**‐*co*‐**11**, **3**‐*co*‐**12**, and **3**‐*co*‐**13** (1:1), did not show any differences in activity, with the *K_M_*‐values (206.6 – 216.9 µm) and *v_max_* (6.9 – 8.0 nm min^−1^) lower compared to homo‐cSAP **3**. The Michaelis‐parameters of the co‐assemblies are summarized in Table S3.

Based on the results obtained from the Michaelis–Menten kinetics, the increase in activity seems to be promoted by a better substrate affinity and a more reactive active site (Figure 4f,g). When comparing *K_M_*‐values, it is observed that all co‐assemblies exhibit lower *K_M_*‐values and therefore better substrate affinities than homo‐assembly **3**. In between the co‐assemblies however, no major differences in substrate affinity are observed. Nonetheless, the difference in catalytic rates between the co‐assemblies (Figure 4g) could suggest changes in substrate proximity to the active lysine side chain, depending on the nature of the co‐assembling partner.

**Figure 5:**
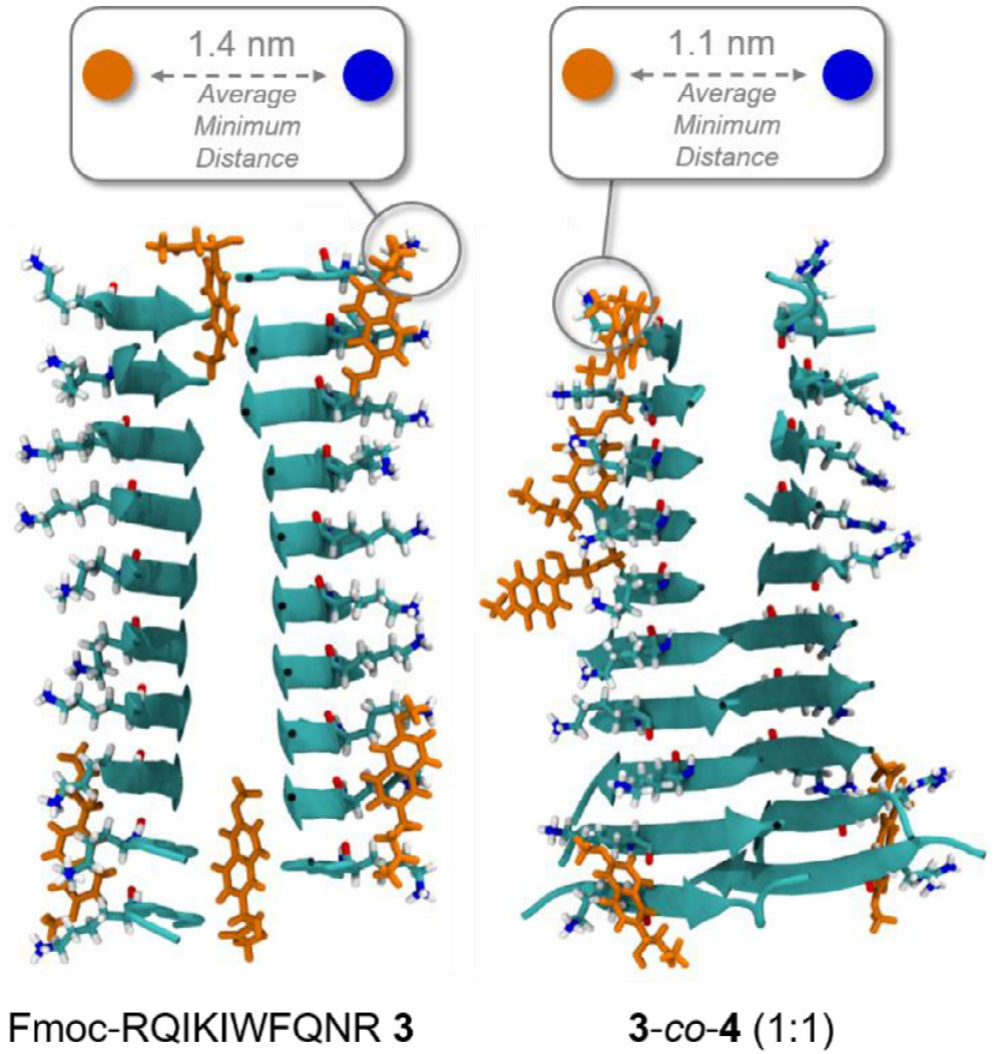
Representative structures of Methodol **1** interactions with Fmoc‐RQIKIWFQNR **3** and **3**‐*co*‐**4** fibrils (1:1). One of the most dominant binding motifs of five Methodol **1** molecules (in orange) to the peptide fibrils are shown. The representative structures were determined using MD simulations and clustering analysis. The fibrils are visualized as cartoons with Lys and Arg residues (position 4) highlighted. The average minimum distance of Methodol **1** to any of the Lys or Arg residues (position 4) is smaller for **3**‐*co*‐**4** (1.1 ± 0.1) nm compared to Fmoc‐RQIKIWFQNR **3** (1.4 ± 0.1) nm, indicating more frequent interactions of Lysine and Methodol **1** in **3**‐*co*‐**4** (1:1).

Cluster analysis of the MD simulations of the most active co‐assembly **3**‐*co*‐**4** revealed differences in average minimum distance between Methodol **1** and the active Lys side‐chain of the peptide fibril compared to the homo‐cSAP **3**. These results indicate that the increase in catalytic rates could be caused by the increased frequency at which substrate **1** is in close vicinity to the active amino‐group of the co‐assembly. For the homo‐cSAP **3** an average minimum distance of (1.4 ± 0.1) nm was obtained, whereas the distance decreased to (1.1 ± 0.1) nm for **3**‐*co*‐**4** (Figure 5).

It is expected that the positive guanidino‐group in Arg increases electrostatic attraction, whereas the negatively charged acidic group in Glu may reduce the frequency for interaction. For the co‐assemblies **3**‐*co*‐**11**, **3**‐*co*‐**12** and **3**‐*co*‐**13** it is observed that the lower *K_M_*‐value, obtained in the Michaelis‐Menten kinetics, is evened out by a slightly lower reaction rate, thus showing no activity differences after 24 h. The neutral side chains of serine (**11**) and leucine (**12**) seem to not have an impact on the substrate‐binding and positioning. Even the histidine side‐chain (**13**), which could be partly protonated under the chosen buffer conditions, does not affect the activity.

To further test the impact of positively charged side chains on the retro‐aldolase activity of Fmoc‐RQIKIWFQNR **3**, we tested the co‐assembly of our two active sequences **3**‐*co*‐**6** (1:1, 50 µm each peptide). To allow comparability with the co‐assembly systems of active and inactive sequences, we set the overall lysine concentration to 100 µm for all samples. We prepared the homo cSAPs **3** and **6** as well as the separately pre‐assembled mixture **3** + **6** identical to the previous samples and analyzed their activities after 24 h incubation (Figure S39). The separately assembled mixture **3** + **6** matches exactly with the calculated activity for the addition of 50 µm **3** and **6** (158.0 ± 7.3). Interestingly, the co‐assembly **3**‐*co*‐**6** shows an increased activity by (23 ± 7)% compared to homo‐assembly **3** but not exceeding homo‐assembly **6**. The small increment in activity with respect to the separately assembled mixture **3** + **6** suggests that structural defects in co‐assemblies can exert a positive influence on catalytic activity. However, in order to increase catalytic activities beyond its counterparts, one of the co‐assembling component should be intrinsically inactive while at the same time promote substrate interactions to the active site.

The presented results emphasize the potential of co‐assemblies with inactive sequences as a moderating tool to up‐ and down‐regulate the activity of peptide catalyst Fmoc‐RQIKIWFQNR **3** without changing the core nanostructure. This flexible tuning of activities mimics a core facet of natural enzyme modulation while keeping the critical advantages of synthetic peptide assemblies, such as their facile synthesis and environmental robustness. By simply adjusting the amount of co‐monomer on‐demand, substrate conversion speed can be engineered without changing global parameters such as temperature, pH, solvent or laborious (bio)chemical modifications. Hence, these assemblies can be reliably formulated into a broad range of materials to impart catalytic functions.

## Conclusion

In conclusion, twelve peptide sequences were synthesized, with their self‐assembly behavior and retro‐aldol catalytic activity characterized. Through synthetic variations of the lead peptide sequence, hydrophobic amino acids in position three and five have been identified to facilitate the formation of fibrillar structures and the Lys‐side chain has been identified as the catalytically active part of the sequence. Changes of the Lys side chain length have been observed to diminish activity, due to restricted access of the active amino‐group to Methodol **1**. However, exchange of the Ile‐side chains to Phe further increased activity of the peptide fibrils through the formation of stabilized fibrils. Co‐assemblies with Lys→Arg variant Fmoc‐RQIRIWFQNR **4** improved catalytic activities of cSAP **3**, whereas co‐assemblies with negatively charged Lys→Glu variant Fmoc‐RQIEIWFQNR **10** decreased activity. Kinetic analysis and MD simulations suggest that the regulation of catalytic activity is most likely achieved by interactions of the co‐monomers that increase the proximity and frequency at which the substrate accesses the active amino‐group. Here, the positively charged guanidino‐group increased the activity, whereas the negatively charged glutamic acid‐group decreased activity. These results offer insights into the formation of self‐assembled, supramolecular peptide structures and their functionalization to promote the turnover of catalytic reactions in aqueous media at neutral pH. The stability tests on the cSAP structures suggest their utilization under a broader range of conditions, thus offering an alternative, more robust, option to the narrow working conditions of natural enzymes, while preserving the potential of programmable activities.

## Supporting Information

The authors have cited additional references within the Supporting Information.^[^ ^36^, ^50^, ^51^, ^56^, ^57^, ^58^, ^59^, ^60^, ^61^, ^62^, ^63^, ^64^, ^65^, ^66^, ^67^, ^68^, ^69^, ^70^, ^71^, ^72^, ^73^, ^74^, ^75^, ^76^, ^77^, ^78^, ^79^, ^80^, ^81^, ^82^ ^]^

## Conflict of Interests

The authors declare no conflict of interest.

## Supporting information



Supporting Information

## Data Availability

The data that support the findings of this study are available in the Supporting Information of this article.
