# Supplementary material for: Co‐Assemblies Regulate the Catalytic Activity of Peptide Fibrils
Source: Angew Chem Int Ed Engl. 2025 Dec 4;65(2):e11165. doi: 10.1002/anie.202511165 (PMC12790359; doi:10.1002/anie.202511165)
Supplement: Supplementary file 1 — Supporting Information [file ANIE-65-e11165-s001.pdf]

Supporting Information

Co-Assemblies Regulate the Catalytic Activity of Peptide Fibrils

Albin Lahu[1], Shao-Lin Wu[1], Maximilian Schuler[1,2], Dr. Francesca Mazzotta[1], Ardit Ramadani[1], Emirhan Koca[1,2], Dr. Ingo Lieberwirth[1], Prof. Dr. Katharina Landfester[1], Prof. Dr. Torsten John[1,3], Dr. David Y. W. Ng*[1], Prof. Dr. Tanja Weil*[1].

[1] Max Planck Institute for Polymer Research, D-55128 Mainz, Germany.

[2] Max Planck School Matter to Life, D-69120 Heidelberg, Germany.

[3] School of Science, Constructor University, D-28759 Bremen, Germany.

E-Mail: david.ng@mpip-mainz.mpg.de, weil@mpip-mainz.mpg.de.

Table of Contents

1 Materials, Instruments and Methods 4
1.1 Materials 4
1.2 Instruments and Methods 4
1.2.1 Microwave Peptide Synthesizer 4
1.2.2 Thin-Layer Chromatography (TLC) 4
1.2.3 High-Performance Liquid Chromatography (HPLC) 4
1.2.4 Nuclear Magnetic Resonance Spectroscopy (NMR) 5
1.2.5 Liquid Chromatography-Mass Spectrometry (LC-MS) 5
1.2.6 Matrix-Assisted Laser Desorption/Ionization – Time of flight (MALDI-Tof) 5
1.2.7 High-Resolution Mass Spectrometry (High-Res ESI-MS) 5
1.2.8 Self-Assembly 6
1.2.9 Proteostat Aggregation Assay 6
1.2.10 Nile Red Assay 6
1.2.11 Catalytic Activity Measurements 7
1.2.12 Förster Resonance Energy Transfer (FRET) 7
1.2.13 Transmission Electron Microscopy (TEM) 7
1.2.14 Cryogenic Electron Microscopy (cryo-EM) 8
1.2.15 Fourier-Transform Infrared-Spectroscopy (FTIR) 8
1.2.16 Circular Dichroism-Spectroscopy (CD) 8
1.2.17 Assembly Conversion 8
1.2.18 Modelling and Molecular Dynamics Simulations 9
2 Synthesis 12
2.1 Synthesis of Small Molecules 12
2.1.1 4-Hydroxy-4-(6-methoxy-2-naphthalenyl)-2-butanone (*Methodol*) 12
2.2 Synthesis of Peptides 15
2.2.1 General Procedure for Automated Peptide Synthesis 15
2.2.2 General Procedure for Manual Peptide Synthesis 15
2.2.3 Purification of Peptides 16

2.2.4 Synthesis of Fmoc-RQIKIWFQNR 16
2.2.5 Synthesis of Fmoc-RQIRIWFQNR 17
2.2.6 Synthesis of Fmoc-RQI*Dap*IWFQNR 18
2.2.7 Synthesis of Fmoc-RQFKFWFQNR 19
2.2.8 Synthesis of Fmoc-RQKWFQNR 20
2.2.9 Synthesis of RQIKIWFQNR 21
2.2.10 Synthesis of NBD-RQIKIWFQNR 22
2.2.11 Synthesis of Fmoc-RQIEIWFQNR 24
2.2.12 Synthesis of Fmoc-RQISIWFQNR 25
2.2.13 Synthesis of Fmoc-RQILIWFQNR 26
2.2.14 Synthesis of Fmoc-RQIHIWFQNR 27
2.2.15 Synthesis of TAMRA-RQIKIWFQNR 28
3 Self-Assembly and Catalytic Activity 30
3.1 Self-Assembly 30
3.1.1 Assembly Conversion and Critical Aggregation Concentration 30
3.1.2 Proteostat Aggregation Assay 32
3.1.3 Fourier-Transform Infrared-Spectroscopy (FTIR) 33
3.1.4 Circular Dichroism-Spectroscopy (CD) 35
3.1.5 Transmission Electron Microscopy (TEM) 36
3.1.6 Cryogenic Electron Microscopy (cryo-EM) 37
3.1.7 Nile Red Assay 38
3.1.8 Förster Resonance Energy Transfer (FRET) 39
3.2 Catalytic Activity 40
3.2.1 Product Calibration and Fluorescence Intensity 40
3.2.2 Catalytic Activity – Peptide Concentrations 41
3.2.3 Stability Test Fmoc-RQIKIWFQNR 42
3.2.4 Michaelis-Menten Kinetics 44
3.2.5 Co-Assembly of Active Peptides 46
3.2.6 Catalytic Activity – inactive homo-cSAP's 47
3.2.7 Modelling and MD Simulations 47

1 Materials, Instruments and Methods

1.1 Materials

Reagents and solvents were purchased from commercial vendors and used without further purification. For the synthesis of peptides, peptide synthesis grade reagents were used. High performance liquid chromatography (HPLC) was performed using acetonitrile (ACN) in HPLC grade. MilliQ-H_2O for HPLC and reactions was obtained from a Millipore purification system by *Merck Millipore*.

1.2 Instruments and Methods

1.2.1 Microwave Peptide Synthesizer

A *LibertyBlue* automated microwave synthesizer from *CEM Corporation* was used to perform solid-phase peptide synthesis (SPPS). The system was purged with dimethylformamide (DMF) prior and during usage. Piperidine was used for deprotection of the peptide protecting groups and the used amino acids were dissolved in DMF. *N*, *N'*-Diisopropylcarbodiimide (DIC) and ethyl cyanohydroxyiminoacetate (Oxyma Pure) were employed as coupling agents and dissolved in DMF as well.

1.2.2 Thin-Layer Chromatography (TLC)

The analysis of compounds by means of thin layer chromatography (TLC) was performed using *Alugram Sil G/UV*$_{254}$ plates, by *Macherey-Nagel*. The analytes were detected by extinction of ultraviolet (UV) absorption at a wavelength of 254 nm and 365 nm or by staining of the TLC-plate using potassium permanganate solution.

1.2.3 High-Performance Liquid Chromatography (HPLC)

Peptides were purified by preparative HPLC using a setup from *Shimadzu*. For purification, a *Phenomenex Gemini* 5 µm *NX-C18* 110 Å 150 × 30 mm column was used at a flowrate of 25 mL/min. All measurements and purification steps were performed using gradients of ACN and MilliQ-H_2O, each acidified with 0.1 % trifluoroacetic acid (TFA). The absorbance was recorded at wavelengths of 214 and 254 nm.

Analytical measurements were performed on an *Atlantis T3* column (4.6 × 100 mm, 5 µm) at a flowrate of 1 mL/min. All measurements and purification steps were performed using gradients of ACN and MilliQ-H_2O, each acidified with 0.1 % TFA. Absorbance was recorded at 214 and 254 nm wavelengths. For processing of the HPLC spectra, the software *LabSolutions* by *Shimadzu* and *PowerPoint* by *Microsoft Corporation* were used.

1.2.4 Nuclear Magnetic Resonance Spectroscopy (NMR)

^{1}H- and ^{13}C-NMR-measurements were conducted in an *AVANCE 400* spectrometer by *Bruker* at 278 K in deuterated solvents. All deuterated solvents were used without further purification and purchased from *Sigma Aldrich*. The internal standards were set depending on the solvent used.

$CDCl_3$: ^{1}H: δ = 7.26 ppm, ^{13}C: δ = 77.16 ppm, DMSO-d_6: ^{1}H: δ = 2.50 ppm, ^{13}C: δ = 39.52 ppm.

The acquired data was processed using the software *MestReNova 14.2.0* by *Mestrelab Research*. The chemical shifts δ were indicated in parts per million (ppm). Multiplets were abbreviated as following: s = singlet, d = doublet, t = triplet, q = quartet, p = quintet, m = multiplet.

1.2.5 Liquid Chromatography-Mass Spectrometry (LC-MS)

Compounds were analyzed by HPLC-ESI-MS on a LC-MS 2020 from *Shimadzu* using a *Kinetex* 2.6 µm EVO C_{18} 100 Å LC 50 × 2.1 mm column. MilliQ-H_2O, acidified with 0.1 % formic acid, and ACN, acidified with 0.1 % formic acid, were used as solvents for all measurements. The solvent gradient started with 5 % ACN and 95 % MilliQ-H_2O. This solvent ratio was kept constant for 2 min, then the ACN content was linearly increased to 95 % in 14 min. Generated data was processed with *LabSolutions* by *Shimadzu* and *Origin Pro* by *OriginLab*®.

1.2.6 Matrix-Assisted Laser Desorption/Ionization – Time of flight (MALDI-Tof)

For further characterization of peptides, MALDI-Tof spectra were recorded on a *rapifleX* MALDI-Tof/Tof by *Bruker*. The samples were dissolved in ACN, MeOH and/or MilliQ-H_2O and mixed with a saturated solution of the matrix, α-cyano-4-hydroxycinnamic acid (CHCA), in H_2O/ACN (1:1, *v/v*, 0.1 % TFA) prior to the measurement. The software *mMass* and *Origin Pro* by *OriginLab*® were used to process the collected data.

1.2.7 High-Resolution Mass Spectrometry (High-Res ESI-MS)

High-Res-ESI-MS was recorded using a *Synapt* G2-Si mass spectrometer from *Waters Corporation via* electro spray ionization (ESI). The samples were infused at a flow rate of 5 µL/min by a syringe pump (*Legato* 180 from *Kd Scientific*) into the mass spectrometer or using a *Acquity Premier* UPLC system coupled to the mass spectrometer using an *Acquity*™ *Premier* BEH C_{18} column (1.7 µm, 2.1 × 50 mm). MilliQ-H_2O with 0.1 % formic acid (solvent A) and ACN 0.1 % formic acid (solvent B), were used as the mobile phase at a flow rate of 0.6 mL/min. The gradient was t = 0 min, 1 % B; t = 0.2 min, 1 % B; t = 1.5 min, 70 % B; t = 1.8 min, 70 % B; t = 2.0 min, 1 % B, t = 2.5 min, 1 % B, stop, detection UV/Vis detection with Diode Array (200 – 800 nm). Mass calibration was done on the day of sample measurement utilizing

Glu-fibrinopeptide B MS/MS fragmentation from 50 – 2000 *m/z* or based on clusters of sodium iodide in the range of 100 – 5000 *m/z*. The measurements were carried out at capillary voltage of 3 kV, sampling cone 40 V, source offset 80 V, and a source temperature of 100 °C. Nitrogen was used as the desolvation gas at a total flow of 600 – 800 L/h. The data was processed with *MassLynx* software V4.2 (*Waters GmbH*) and with *Origin Pro* by *OriginLab®*.

1.2.8 Self-Assembly

The monomeric peptides were dissolved in DMSO at high concentrations (10 mM) before dilution using phosphate-buffered saline (PBS) buffer (50 mM, pH 7.4). The final peptide concentrations were 100 µM in PBS (50 mM, pH 7.4, 5 % DMSO) unless stated otherwise. For co-assembly, the monomeric peptides were dissolved in DMSO at high concentrations (20 mM), mixed at 1:1 ratio (*v/v*) and ultra-sonicated for 5 min at room temperature before dilution of the mixed peptide stock with PBS buffer (50 mM, pH 7.4). The final peptide concentrations were 200 µM (1:1) in PBS (50 mM, pH 7.4, 5 % DMSO) unless stated otherwise. The samples were incubated at room temperature for 24 h.

1.2.9 Proteostat Aggregation Assay

The Proteostat Protein Aggregation Assay Kit was purchased from *Enzo Life Sciences, Inc.* 0.52 µL of the Proteostat stock solution was diluted with 98.48 µL MilliQ-H_2O and 1.0 µL assay buffer. 5 µL of this solution was added to 45 µL peptide solution in a *Greiner* 96 black well plate. The solutions were incubated in the dark at room temperature for 15 min while shaking at 500 rpm. The fluorescence intensity was measured with bandwidths of 20 nm and an excitation wavelength of λ_{ex} = 550 nm as well as an emission wavelength of λ_{em} = 600 nm. The experiment was repeated four times. Control measurements were performed by incubating Proteostat solution similarly in PBS buffer (50 mM, pH 7.4, 5 % DMSO).

1.2.10 Nile Red Assay

For homo-assemblies, SAPs were prepared as 1 mM stock solutions in DMSO, diluted in PBS buffer (50 mM, pH 7.4) to afford final peptide concentrations of 10 µM (5 % DMSO). For co-assemblies, individual SAPs were prepared as 2 mM stock solutions in DMSO, combined, sonicated for 5 min and then diluted in PBS buffer (50 mM, pH 7.4) to obtain final peptide concentrations of 20 µM (5 % DMSO). All samples were incubated for 24 h in the dark prior addition of Nile Red (from 1.2 mM stock solution in DMSO) with a final Nile Red concentration of 30 µM. The fluorescence intensity was analyzed in a 96-well plate black (*Greiner*) on a *TECAN* Spark 20M microplate reader. A fluorescence intensity scan was measured (λ_{ex} = 550 nm, λ_{em} = 620 – 700 nm) and λ_{max} determined. The data was processed using *Excel* by *Microsoft Corporation* and plotted using *Origin Pro* by *OriginLab®*.

1.2.11 Catalytic Activity Measurements

Samples with pre-assembled peptide fibers (100 µM) in PBS (50 mM, pH 7.4, 5 % DMSO) were prepared through addition of Methodol **1** (50 µM) and 6-methoxy-2-naphthaldehyde **2** (5 µM). Another set of peptide samples without Methodol **1** was prepared in the same way and later used for background subtraction. The peptide samples were incubated in the dark at 37 °C for 24 h. Control samples without peptide in PBS (50 mM, pH 7.4, 5 % DMSO) were also prepared and incubated in the dark at 37 °C for 24 h to analyze the stability of Methodol **1** in the chosen buffer system. After incubation, all samples were analyzed in triplicates regarding their fluorescence emission intensity on a *Greiner* 96 black well plate at λ_{ex} = 330 nm and λ_{em} = 452 nm on a *TECAN* Spark 20M microplate reader. The data was processed using *Excel* by *Microsoft Corporation* and plotted using *Origin Pro* by *OriginLab*®.

1.2.12 Förster Resonance Energy Transfer (FRET)

The monomeric peptides were dissolved in DMSO at high concentrations (10 mM), mixed at 1:1 ratio (*v/v*) and ultra-sonicated for 5 min at room temperature before dilution of the mixed peptide stock with PBS buffer (50 mM, pH 7.4). The final peptide concentrations were 50 µM for the homo-assemblies and 100 µM (1:1) for the co-assemblies in PBS (50 mM, pH 7.4, 5 % DMSO). The samples were incubated for 24 h at room temperature and analyzed regarding their fluorescence in a 96-well plate black (*Greiner*) at λ_{ex} = 464 nm and λ_{em} = 500 – 700 nm on a *TECAN* Spark 20M microplate reader. The data was processed using *Excel* by *Microsoft Corporation* and plotted using *Origin Pro* by *OriginLab*®.

1.2.13 Transmission Electron Microscopy (TEM)

TEM images were recorded on a *JEOL 1400* transmission electron microscope at a voltage of 120 kV. The images were visualized using the software *ImageJ*. Unless stated otherwise, all samples were prepared in PBS (50 mM, pH 7.4, 5 % DMSO) and incubated for 24 h at room temperature. The samples were applied to plasma-cleaned Formvar/carbon-film coated copper grids (300 mesh) by *Plano GmbH*. The TEM grids were prepared by covering the grid with a 3 µL droplet of the sample solution and incubation for 5 minutes. The excess solution was removed and the grids were stained with 7 µL uranyl (IV) acetate solution (4 %) for 2.5 min. The TEM grids were washed three times with MilliQ-H_2O and dried before being used for microscopy.

1.2.14 Cryogenic Electron Microscopy (cryo-EM)

The pre-assembled sample (3 µL) in PBS (50 mM, pH 7.4, 5 % DMSO) was placed onto a Quantifoil 1.2/1.3 Cu 400 mesh grid (previously glow discharged). The grid was blotted and plunged into liquid ethane with an automated plunging system (*Vitrobot* from *Thermo-Fisher Scientific*) and transferred in liquid nitrogen to the TEM (*Titan Krios G4* from *Thermo-Fisher Scientific*). The TEM was operated at 300 kV. Micrographs were acquired using a *Gatan K3* camera with 20 eV energy.

1.2.15 Fourier-Transform Infrared-Spectroscopy (FTIR)

Fourier-Transform Infrared Spectroscopy (FTIR) spectra were measured on a *Bruker* TENSOR II spectrometer equipped with a PLATINUM ATR single reflection diamond accessory. Acquired data was processed using *Opus* from *Bruker* and *Excel* from *Microsoft Corporation*.

1.2.16 Circular Dichroism-Spectroscopy (CD)

CD spectra were recorded on a *JASCO* J-1500 spectrometer in a 1 mm High Precision Cell by *Hellma Analytics*. The collected data was processed using *Spectra Analysis* by *JASCO* and *Origin Pro* by *OriginLab*®. Samples were prepared in 50 mM PBS buffer (pH 7.4) with peptide concentrations of 100 µM and 5 % 2,2,2-trifluoroethanol (TFE). The samples were incubated for 24 h at room temperature and the spectra were recorded at wavelengths from 185 nm to 300 nm with a bandwidth of 1 nm, data pitch of 0.2 nm and scanning speed at 5 nm/min, while using 300 µL of the prepared sample solution. The samples were measured three times and the collected spectra were averaged. Background measurements were performed using PBS buffer (50 mM, pH 7.4, 5 % TFE, 300 µL) and subtracted from the peptide spectra.

1.2.17 Assembly Conversion

The monomeric peptides were dissolved in DMSO at high concentrations (10 mM) before dilution to different concentrations using PBS (50 mM, pH 7.4) and methanol. The final peptide concentrations were 100, 75, 50, 25 µM in PBS (50 mM, pH 7.4, 5 % DMSO) and 100, 75, 50, 25 µM in MeOH (5 % DMSO). The samples were incubated at room temperature for 24 h. Then, the samples were filtered through a syringe filter (0.2 µm) and diluted with an internal standard, Fmoc-Trp(Boc)-OH (100 µM in MeOH, 1:1, *v/v*). Assembly conversion rate measurements were performed on an *Atlantis T3* column (4.6 × 100 mm, 5 µm) at a flowrate of 1 mL/min. All measurement steps were performed using gradients of ACN and MilliQ-H_2O, each acidified with 0.1 % TFA. The gradient started at 0 % ACN content and was linearly increased to 100 % over 24 min. Absorbance was recorded at 214 and 254 nm wavelengths. The software *LabSolutions* by *Shimadzu* and *Origin Pro* by *OriginLab*® were used to process the generated HPLC spectra.

1.2.18 Modelling and Molecular Dynamics Simulations

Prediction of Fibril Structures using *AlphaFold* 3

The *AlphaFold* 3 (AF3) program, which is based on deep learning, was used to obtain an initial model of the peptide structures without Fmoc.[1] The AF3 server (https://alphafoldserver.com) was used to predict structural models consisting of 20 peptide monomers each. The AF3 results are subject to the *AlphaFold* Server Output Terms of Use found at https://alphafoldserver.com/output-terms. The five most likely models with confidentiality scores were carefully assessed and a representative structure was used before addition of the Fmoc-group. AF3 suggested in most cases that a 10-strand long double β-sheet structure (10 x 2) represents a stable fibril model. By default, the top structural model (model 0) was chosen, except for RQIRIWFQNR sequence where model 1 was used as it provided a 10 x 2 fibril, consistent with the other peptides. Structural models were visualized with *VMD* 1.9.3.[2] Fmoc-residues were added to the AF3 fibril models using *BIOVIA Discovery Studio Visualizer* v21.1.0 (*Dassault Systèmes*, Vélizy-Villacoublay, France).

Parametrization of Force Field Parameters for Fmoc and Methodol

Since the Fmoc-modification of the peptides and Methodol **1** are not by default parametrized in the used *CHARMM36m* force field[3-4], these needed to be specifically prepared. The chemical structures of Fmoc and Methodol **1** were first drawn in *ChemDraw* 22.2.0 (*PerkinElmer*, Waltham, MA) and exported as mol file, followed by conversion into a mol2 file using *Avogadro* 1.2.0.[5] The *CGenFF* web app (app.cgenff.com) was used to generate *CHARMM36M* compatible parameters for usage with *GROMACS*. It applies the *CHARMM General Force Field* (*CGenFF*).[6-8] Fmoc parameters were incorporated into the force field as a new protein residue typ.

Molecular Dynamics (MD) Simulations

The Fmoc-fibril models were placed in a cubic box with at least 1 nm distance between fibril and simulation box. The peptide fibrils were solvated in explicit water using the *TIP3P* water model[9] and sodium chloride (140 mM) was added as physiological salt and to neutralize the peptide charges. Each fibril system was energy minimized using a steepest-descent algorithm, followed by two equilibration steps with position restrained peptides (k = 1000 kJ mol^{-1} nm^{-2}): 100 ps in a NVT ensemble and 100 ps in a NPT ensemble. Each fibril system was then studied by MD simulation for 100 ns in triplicate with random starting velocities following a Boltzmann distribution at 295 K. The final 10 ns of above simulations (triplicate; 3 x 10 ns) for each peptide fibril were used to determine the most representative structure using clustering analysis (*gmx cluster*, gromos method, RMSD cutoff 0.35 – 0.44 nm).[10]

The RMSF cutoff has been chosen so that almost all frames become part of one cluster and the central structure of all frames was used as representative structure. The now equilibrated peptide fibril has been placed in a cubic box with at least 1 nm distance between fibril and simulation box, followed by addition of five Methodol **1** molecules. This was followed by solvation in explicit water using the *TIP3P* water model and addition of sodium chloride (140 mM). Each system was again energy minimized using a steepest-descent algorithm, followed by two equilibration steps with position restrained peptides and substrate **1** molecules (k = 1000 kJ mol^{-1} nm^{-2}): 100 ps in a NVT ensemble and 100 ps in a NPT ensemble. Each system was then studied by MD simulation for 300 ns in triplicate with random starting velocities following a Boltzmann distribution at 295 K. An overview of simulation times is included in Table S1.

Table S1: Overview of MD simulations with simulation times and number of replicates for Fmoc-RQIKIWFQNR **3**, Fmoc-RQIRIWFQNR **4**, Fmoc-RQFKFWFQNR **6** and **3**-*co*-**4** (1:1).

Peptide	Fibril Stability (20mer) [ns]	Fibril (20mer) & 5 Methodol [ns]
Fmoc-RQIKIWFQNR **3**	3 x 100	3 x 300
Fmoc-RQIRIWFQNR **4**	3 x 100	3 x 300
Fmoc-RQFKFWFQNR **6**	3 x 100	3 x 300
**3**-*co*-**4**	3 x 100	3 x 300

MD simulations were performed in *GROMACS* version 2021.7 (preparation and analysis in version 2024.4)[11-18] using published parameters.[19] In short, a time step of 2 fs was used for all simulations and the all-atom additive *CHARMM36m* force field was applied to describe peptides, solvent and ions.[3-4] Periodic boundary conditions were applied. The Verlet cutoff scheme was used for neighbor search. A single cutoff of 1.2 nm was set to calculate electrostatic and van der Waals interactions. A force switch was applied to smoothly switch the van der Waals forces to zero between 1.0 nm and 1.2 nm. Long-range electrostatic interactions beyond the cutoff were described using the Particle-Mesh Ewald (PME) method.[20] All bonds with H-atoms were constrained to their equilibrium values using the *LINCS* algorithm[21-22]; water molecules were constrained using the *SETTLE* algorithm.[23] The center of mass translational velocity of the system was removed. The temperatures of “peptides”, and “Methodol **1**, water and ions”, were independently coupled to an external bath at 295 K using the v-rescale thermostat with a relaxation time of 0.1 ps.[24]

Isotropic pressure coupling to maintain a pressure of 1 bar was achieved using the *Berendsen* barostat during equilibration[25] and the *Parrinello-Rahman* barostat during production MD (relaxation time 2 ps, compressibility of 0.000045 bar^{-1}).[26]

MD simulations were analyzed using *GROMACS* tools. To determine characteristic stable Methodol **1**-fibril binding motifs, clustering analysis was used for the entire 300 ns simulations (triplicates) (*gmx cluster*, gromos method, RMSD cutoff 0.5 nm).[10] The central structures of the largest clusters were visualized using *VMD* 1.9.3.[2] To determine how close substrate **1** was located near the Lys or Arg residues (position 4) in the peptide fibrils, the shortest distance between any Methodol **1** molecule and any of the Lys or Arg residues (position 4) was determined over time (*gmx mindist*). *Origin Pro* by *OriginLab*® was used to process and plot the data.

2 Synthesis

2.1 Synthesis of Small Molecules

2.1.1 4-Hydroxy-4-(6-methoxy-2-naphthalenyl)-2-butanone (*Methodol*)

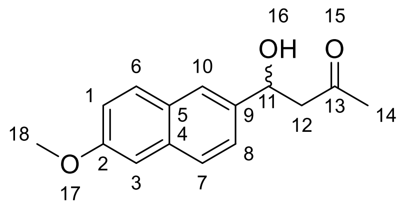

1

The synthesis was performed according to a general procedure from Schmidt *et al.*[27] Lithium diisopropylamide (LDA, 1.6 mL; 12 mmol; 2.0 eq.) was dissolved in dry THF (20 mL) under nitrogen atmosphere at -78 °C. After 20 min, a solution of freshly distilled acetone (0.89 mL; 12 mmol; 2.0 eq.) was added dropwise. The mixture was stirred for 30 min at -78 °C. A solution of 6-methoxy-2-naphthaldehyde **2** (1.00 g; 6.0 mmol; 1.0 eq.) in dry THF (10 mL) was added dropwise while stirring. After 15 min, the reaction was quenched by adding a solution of saturated NH_4Cl (5 mL) dropwise. The reaction mixture was warmed to room temperature and diluted with diethyl ether (150 mL). The organic layer was washed with H_2O (2 x 50 mL), dried over $MgSO_4$, filtered and concentrated under reduced pressure. A yellow crude was obtained, which was purified using column chromatography on silica gel with EtAc/^{C}Hex (1:2, *v/v*). 4-Hydroxy-4-(6-methoxy-2-naphthalenyl)-2-butanone **1** (963 mg; 3.9 mmol; 73 %) was obtained as a colorless solid.

LC-MS: (pos.): calculated for $[M + H - OH]^+$: 227.1, measured: 227.1.

High-Res-ESI: (pos.): calculated for $C_{15}H_{16}NaO_3^+$ $[M + Na]^+$: 267.0992, measured: 267.1404; calculated for $C_{15}H_{15}O_2^+$ $[M + H - OH]^+$: 227.1067, measured: 227.1111.

R_f: 0.31 (EtAc/^{C}Hex = 1:2, *v/v*).

^{1}H-NMR, COSY (400 MHz, $CDCl_3$, 298 K): δ [ppm] = 7.73 (dd, 3H, J = 11.1 Hz, 2.7 Hz, $H_{6,7,10}$); 7.46 – 7.39 (m, 1H, H_8); 7.19 – 7.09 (m, 2H, $H_{1,3}$); 5.33 – 5.26 (m, 1H, H_{11}); 3.92 (s, 3H, H_{18}); 3.32 (d, 1H, J = 3.0 Hz, H_{16}); 3.03 – 2.84 (m, 2H, H_{12}); 2.21 (s, 3H, H_{14}).

^{13}C-NMR, HSQC, HMBC (101 MHz, $CDCl_3$, 298 K): δ [ppm] = 209.28, 157.89, 137.95, 134.26, 129.59, 128.88, 127.35, 124.43, 119.20, 105.81, 70.13, 55.45, 52.09, 30.97.

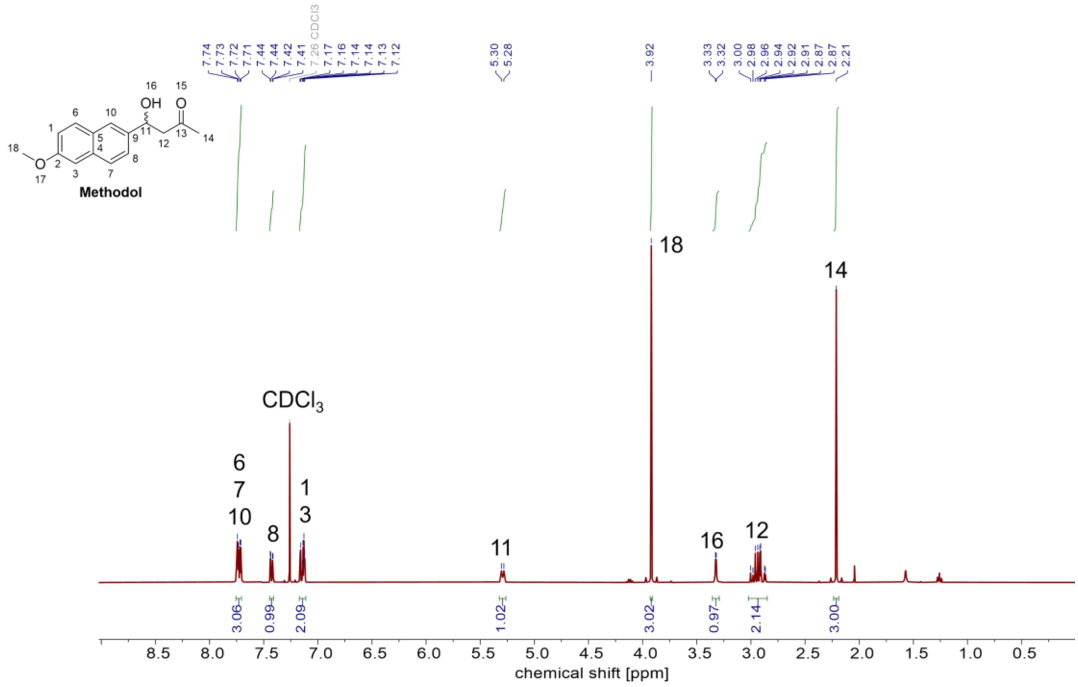

Figure S1: ^{1}H-NMR spectrum ($CDCl_3$, 400 MHz, 298 K) of Methodol **1**.

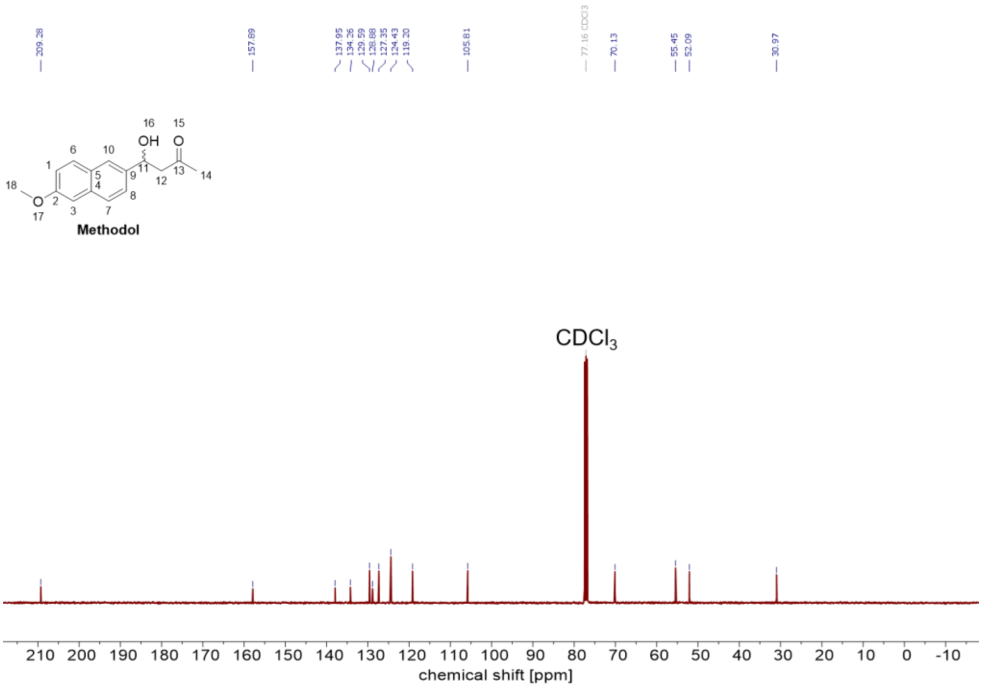

Figure S2: ^{13}C-NMR spectrum ($CDCl_3$, 101 MHz, 298 K) of Methodol **1**.

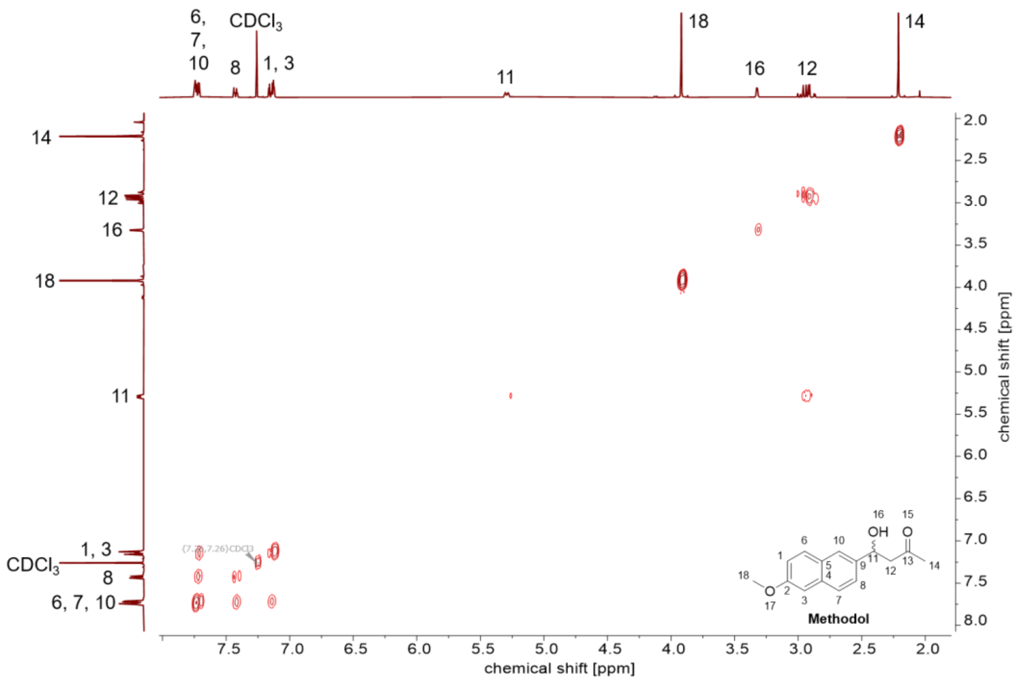

Figure S3: ${}^{1}H$–${}^{1}H$-COSY-NMR spectrum ($CDCl_3$, 400 MHz, 298 K) of Methodol **1**.

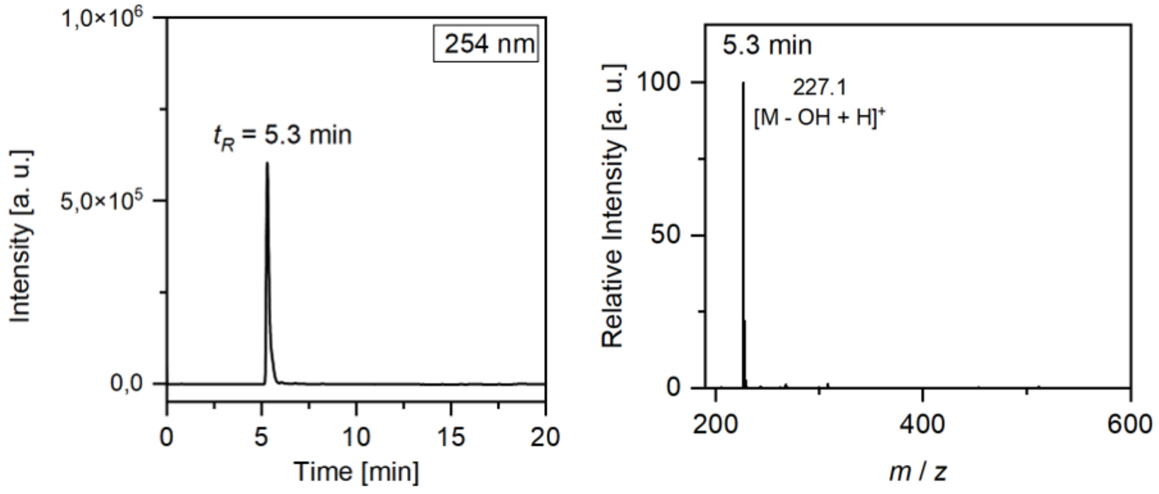

Figure S4: LC elugram (left, detection at 254 nm) and corresponding mass data (right) of Methodol **1**.

2.2 Synthesis of Peptides

2.2.1 General Procedure for Automated Peptide Synthesis

Peptides were synthesized using the Fmoc-SPPS strategy[28], synthesizing the peptide from *C*- to *N*-terminus in a microwave-assisted peptide synthesizer on a Fmoc-L-Arg(Pbf) *Wang*-resin at scales of 0.10 mmol. Fmoc-L-Arg(Pbf) *Wang*-resin (0.10 mmol) was swollen in DMF (5 mL) at room temperature for one hour before transferring it into the peptide synthesizer. DMF was removed *via* a draining process and the resin was swollen with DMF (20 mL) for 20 s. DMF was removed again through the draining process. Prior to every coupling, the Fmoc-protecting group was cleaved by two deprotection steps using a solution of 20 % piperidine (DMF, 3 mL) for two and five minutes at 75 °C. The solvent was drained and the resin was washed three times with DMF. The amino acids (0.2 M in DMF), DIC (0.5 M in DMF) and Oxyma Pure (1.0 M in DMF) were added to the resin and the solution was heated to 70 °C for 30 s followed by a heating cycle to 90 °C for 120 s. The reaction mixture was drained, the Fmoc-group was removed as described before and washed three times with DMF. The following coupling steps were performed in the same way.

2.2.2 General Procedure for Manual Peptide Synthesis

Peptides were synthesized using the Fmoc-SPPS strategy[28], synthesizing the peptide from *C*- to *N*-terminus in a heat-assisted set-up with nitrogen-flow on a Fmoc-L-Arg(Pbf) *Wang*-resin at scales of 0.10 and 0.25 mmol. Fmoc-L-Arg(Pbf) *Wang*-resin (0.10/0.25 mmol) was swollen in DMF (5 mL) at room temperature for one hour before transferring it into the synthesis set-up. DMF was removed through vacuum-assisted filtering. Prior to every coupling, the Fmoc-protecting group was cleaved by two deprotection steps using a solution of 5 % piperazine (DMF, 5 mL), which contained 1-hydroxybenzotriazole (HOBt, 0.2 M), for five minutes at 68 °C. The solvent was filtered and the resin was washed four times with DMF. The amino acids (0.2 M in DMF) were coupled twice to the resin using *O*-(1*H*-6-chlorobenzotriazole-1-yl)-1,1,3,3-tetramethyluronium hexafluorophosphate (HCTU, 0.4 M in DMF) and *N, N'*-diisopropylethylamine (*N*, *N'*-DIPEA, 0.4 M in DMF) under heating of the solution to 68 °C for 5 min. The reaction mixture was filtered, the Fmoc-group was removed as described before and the resin was washed four times with DMF. The following coupling steps were performed in the same way.

2.2.3 Purification of Peptides

Crude peptide products were purified *via* HPLC. The crudes were dissolved in 20 mL of ACN:MilliQ-H_2O (9:1, *v/v*, 5 % TFA) and filtered through a syringe filter (0.2 µm). For purification, a *Phenomenex Gemini* 5 µm NX-C_{18} 110 Å 150 × 30 mm column was used at a flowrate of 25 mL/min. The gradient started at 0 % ACN (0.1 % TFA) and was linearly increased to 100 % ACN (0.1 % TFA) through the span of 20 min. Fractions were collected and detected by measurements of the absorbance at wavelengths of 214 and 254 nm. The compounds were then identified using LC-MS and MALDI-Tof. The product fractions were collected in a round-bottom flask (RBF) and narrowed down to 40 mL under reduced pressure. The residual solvents were removed *via* lyophilization overnight.

2.2.4 Synthesis of Fmoc-RQIKIWFQNR

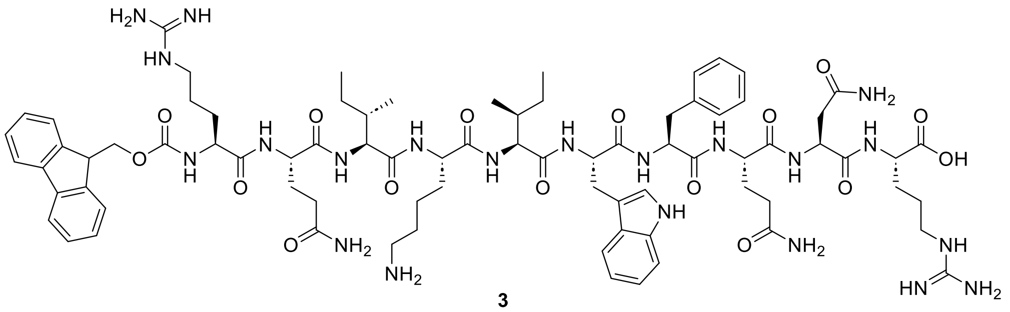

3

Fmoc-RQIKIWFQNR **3** was synthesized using SPPS at a scale of 0.10 mmol on Fmoc-Arg(Pbf)-*Wang*-resin by coupling of Fmoc-Arg(Pbf)-OH (0.2 M in 3 mL DMF), Fmoc-Gln(Trt)-OH (0.2 M in 6 mL DMF), Fmoc-Ile-OH (0.2 M in 6 mL DMF), Fmoc-Lys(Boc)-OH (0.2 M in 3 mL DMF), Fmoc-Trp(Boc)-OH (0.2 M in 3 mL DMF), Fmoc-Phe-OH (0.2 M in 3 mL DMF) and Fmoc-Asn(Trt)-OH (0.2 M in 3 mL DMF) according to the general procedure for automated peptide synthesis. After the final coupling, the resin was washed with DMF (3 x 5 mL) and DCM (3 x 5 mL) and treated with a cleavage cocktail consisting of 95 % TFA, 2.5 % TIPS and 2.5 % H_2O for three hours. The resin was filtered and washed with TFA (1 mL). The crude product was precipitated from cold ether (60 mL), centrifuged (4000 rpm, 0 °C, 20 min) and the supernatant was decanted. Upon drying, a colorless solid crude was obtained. The crude was purified using HPLC according to the general procedure. The product eluted after 8.50 min. Fmoc-RQIKIWFQNR **3** (80 mg; 0.047 mmol; 47 %) was obtained as a colorless solid (three attached TFA ions estimated).

LC-MS: (pos.): calculated for $[M + 2 H]^{2+}$: 806.5, measured: 806.5; calculated for $[M + H]^{+}$: 1611.9, measured: 1612.1.

High-Res-ESI: (pos.): calculated for $C_{79}H_{114}N_{21}O_{16}^{3+}$ [M + 3 H]$^{3+}$: 537.6245, measured: 537.6240; calculated for $C_{79}H_{113}N_{21}O_{16}^{2+}$ [M + 2 H]$^{2+}$: 805.9332, measured: 805.9406.

MALDI-Tof: calculated for [M + H]$^{+}$: 1611.9, measured: 1610.7.

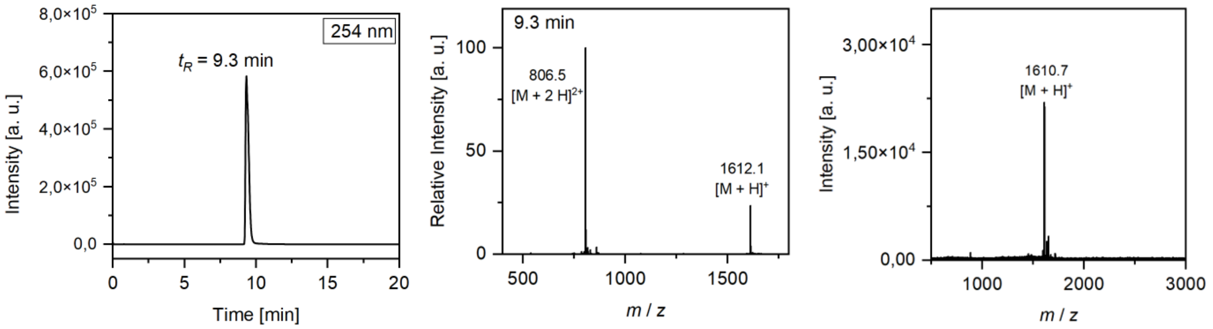

Figure S5: LC elugram (left, detection at 254 nm), corresponding mass data (middle) and MALDI-Tof-MS spectrum (right) of Fmoc-RQIKIWFQNR **3**.

2.2.5 Synthesis of Fmoc-RQIRIWFQNR

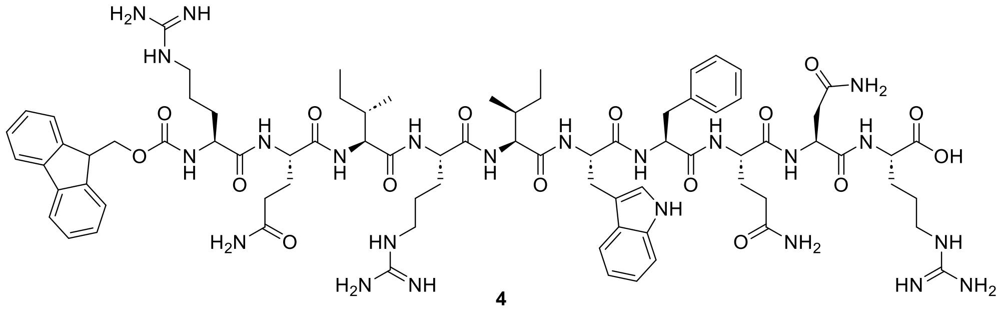

4

Fmoc-RQIRIWFQNR **4** was synthesized using SPPS at a scale of 0.10 mmol on Fmoc-Arg(Pbf)-*Wang*-resin by coupling of Fmoc-Arg(Pbf)-OH (0.2 M in 6 mL DMF), Fmoc-Gln(Trt)-OH (0.2 M in 6 mL DMF), Fmoc-Ile-OH (0.2 M in 3 mL DMF), Fmoc-Trp(Boc)-OH (0.2 M in 3 mL DMF), Fmoc-Phe-OH (0.2 M in 3 mL DMF) and Fmoc-Asn(Trt)-OH (0.2 M in 3 mL DMF) according to the general procedure for automated peptide synthesis. After the final coupling step, the resin was washed with DMF (3 x 5 mL) and DCM (3 x 5 mL). The resin was treated with a cleavage cocktail consisting of 95 % TFA, 2.5 % TIPS and 2.5 % H_2O for three hours. Then, the resin was washed with TFA (1 mL). A crude product was precipitated from cold ether (60 mL), centrifuged (4000 rpm, 0 °C, 20 min) and the supernatant was decanted. Upon drying, a colorless solid crude was obtained. The crude was purified using HPLC according to the general procedure. The product eluted after 8.50 min. Fmoc-RQIRIWFQNR **4** (86 mg; 0.043 mmol; 43 %) was obtained as a colorless solid (three attached TFA ions estimated).

LC-MS: (pos.): calculated for [M + 2 H]$^{2+}$: 820.5, measured: 820.6; calculated for [M + H]$^{+}$: 1639.9, measured: 1639.9.

High-Res-ESI: (pos.): calculated for $C_{79}H_{114}N_{23}O_{16}^{3+}$ [M + 3 H]$^{3+}$: 546.9599, measured: 546.9622; calculated for $C_{79}H_{114}N_{23}O_{16}^{2+}$ [M + 2 H]$^{2+}$: 819.9363, measured: 819.9478.

MALDI-Tof: calculated for [M + H]$^{+}$: 1639.9, measured: 1639.9.

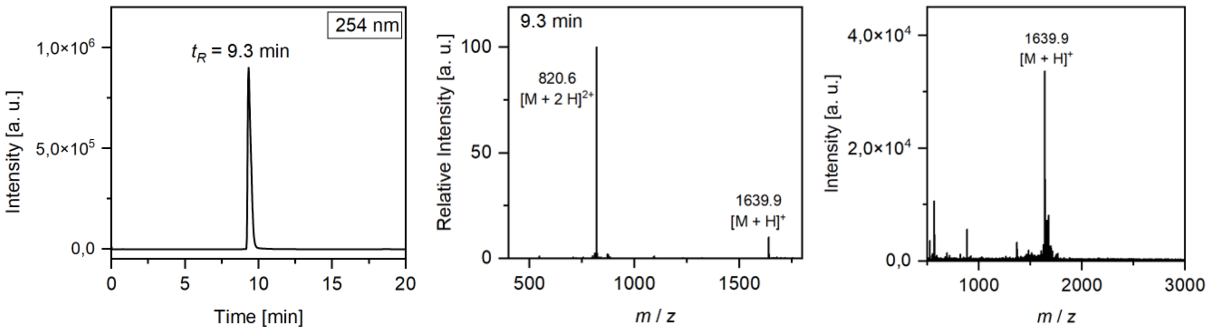

Figure S6: LC elugram (left, detection at 254 nm), corresponding mass data (middle) and MALDI-Tof-MS spectrum (right) of Fmoc-RQIRIWFQNR **4**.

2.2.6 Synthesis of Fmoc-RQI*Dap*IWFQNR

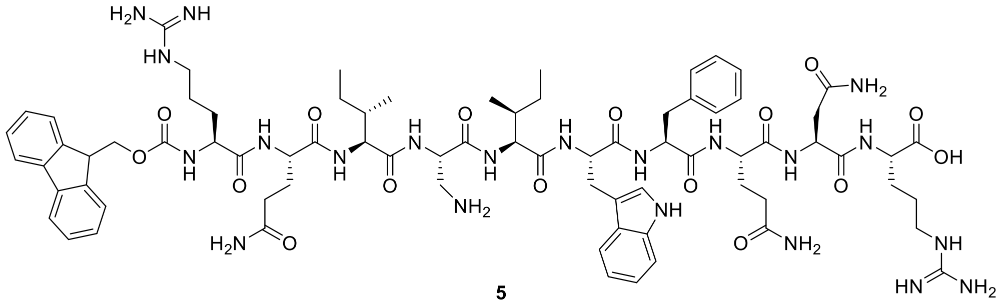

5

Fmoc-RQI*Dap*IWFQNR **5** was synthesized using SPPS at a scale of 0.10 mmol on Fmoc-Arg(Pbf)-*Wang*-resin by coupling of Fmoc-Arg(Pbf)-OH (0.2 M in 3 mL DMF), Fmoc-Gln(Trt)-OH (0.2 M in 6 mL DMF), Fmoc-Ile-OH (0.2 M in 3 mL DMF), Fmoc-Dap(Boc)-OH (0.2 M in 3 mL DMF), Fmoc-Trp(Boc)-OH (0.2 M in 3 mL DMF), Fmoc-Phe-OH (0.2 M in 3 mL DMF) and Fmoc-Asn(Trt)-OH (0.2 M in 3 mL DMF) according to the general procedure for automated peptide synthesis. After the final coupling step, the resin was washed with DMF (3 x 5 mL) and DCM (3 x 5 mL). The resin was treated with a cleavage cocktail consisting of 95 % TFA, 2.5 % TIPS and 2.5 % H_2O for three hours. Then, the resin was washed with TFA (1 mL). A crude product was precipitated from cold ether (60 mL), centrifuged (4000 rpm, 0 °C, 20 min) and the supernatant was decanted. Upon drying, a colorless solid crude was obtained. The crude was purified using HPLC according to the general procedure. The product eluted after 8.50 min. Fmoc-RQI*Dap*IWFQNR **5** (56 mg; 0.031 mmol; 31 %) was obtained as a colorless solid (three attached TFA ions estimated).

LC-MS: (pos.): calculated for $[M + 3\ H]^{3+}$: 523.6, measured: 523.9; calculated for $[M + 2\ H]^{2+}$: 784.9, measured: 785.4; calculated for $[M + H]^{+}$: 1568.8, measured: 1569.9.

High-Res-ESI: (pos.): calculated for $C_{76}H_{108}N_{21}O_{16}^{3+}$ $[M + 3\ H]^{3+}$: 523.6089, measured: 523.9456; calculated for $C_{76}H_{107}N_{21}O_{16}^{2+}$ $[M + 2\ H]^{2+}$: 784.9097, measured: 784.9134.

MALDI-Tof: calculated for $[M + H]^{+}$: 1568.8, measured: 1568.7.

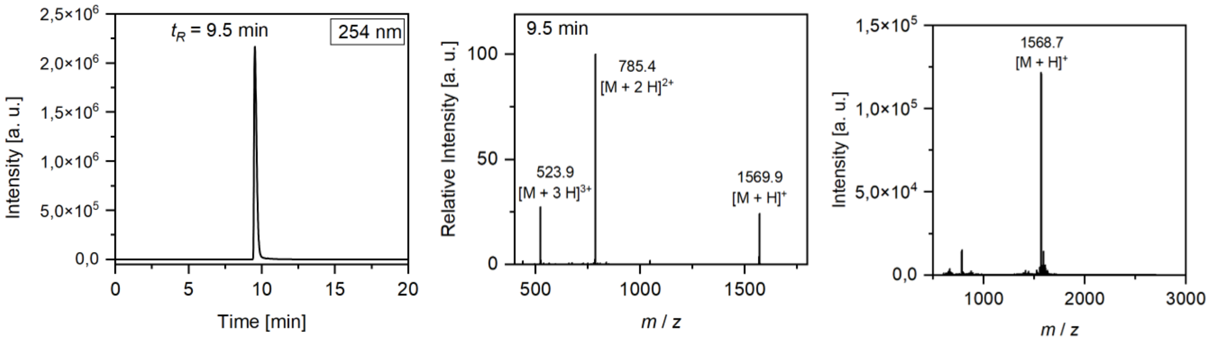

Figure S7: LC elugram (left, detection at 254 nm), corresponding mass data (middle) and MALDI-Tof-MS spectrum (right) of Fmoc-RQI*Dap*IWFQNR **5**.

2.2.7 Synthesis of Fmoc-RQFKFWFQNR

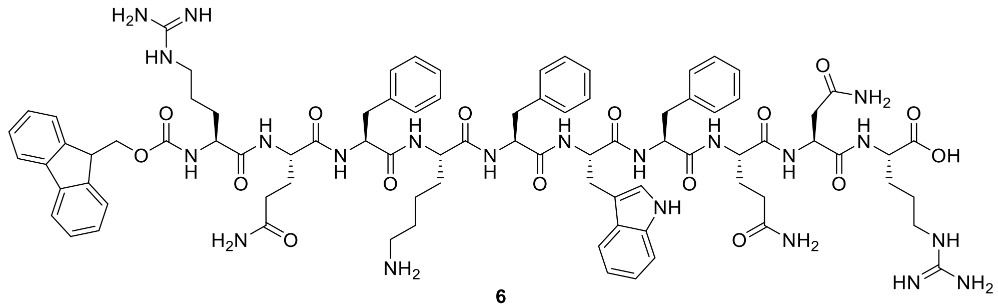

6

Fmoc-RQFKFWFQNR **6** was synthesized using SPPS at a scale of 0.10 mmol on Fmoc-Arg(Pbf)-*Wang*-resin by coupling of Fmoc-Arg(Pbf)-OH (0.2 M in 6 mL DMF), Fmoc-Gln(Trt)-OH (0.2 M in 6 mL DMF), Fmoc-Lys(Boc)-OH (0.2 M in 3 mL DMF), Fmoc-Trp(Boc)-OH (0.2 M in 3 mL DMF), Fmoc-Phe-OH (0.2 M in 12 mL DMF) and Fmoc-Asn(Trt)-OH (0.2 M in 3 mL DMF) according to the general procedure for automated peptide synthesis. After the final coupling, the resin was washed with DMF (3 x 5 mL) and DCM (3 x 5 mL). The resin was treated with a cleavage cocktail consisting of 95 % TFA, 2.5 % TIPS and 2.5 % H_2O for three hours. The mixture was filtered and washed with TFA (1 mL). A crude product was precipitated from cold diethyl ether (60 mL), centrifuged (4000 rpm, 0 °C, 20 min) and the supernatant was decanted. Upon drying, a colorless solid crude was obtained. The crude was purified using HPLC according to the general procedure. The product eluted after 8.30 min.

Fmoc-RQFKFWFQNR **6** (80 mg; 0.042 mmol; 42 %) was obtained as a colorless solid (three attached TFA ions estimated).

LC-MS: (pos.): calculated for $[M + 2 H]^{2+}$: 840.5, measured: 840.6; calculated for 1679.9 $[M + H]^{+}$: 1679.9, measured: 1679.9.

High-Res-ESI: (pos.): calculated for $C_{85}H_{110}N_{21}O_{16}^{3+}$ $[M + 3 H]^{3+}$: 560.2808, measured: 560.2907; calculated for $C_{85}H_{109}N_{21}O_{16}^{2+}$ $[M + 2 H]^{2+}$: 839.9175, measured: 839.9190.

MALDI-Tof: calculated: 1679.9 $[M + H]^{+}$, measured: 1679.8 $[M + H]^{+}$.

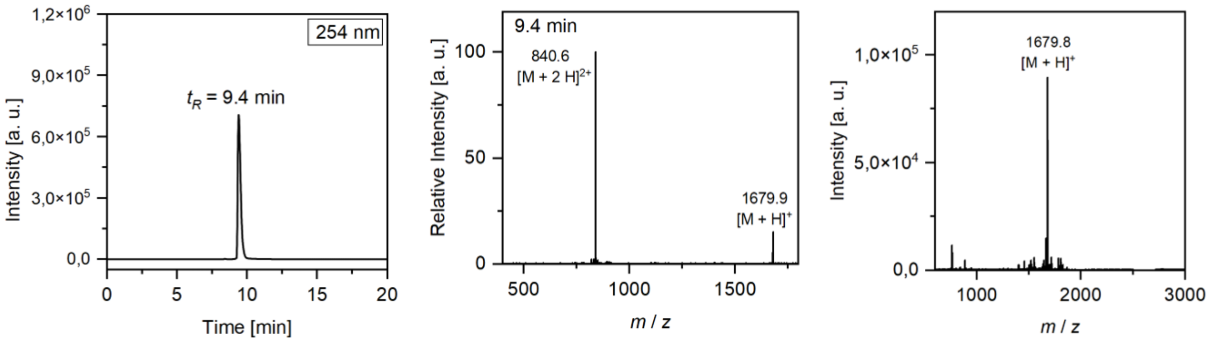

Figure S8: LC elugram (left, detection at 254 nm), corresponding mass data (middle) and MALDI-Tof-MS spectrum (right) of Fmoc-RQFKFWFQNR **6**.

2.2.8 Synthesis of Fmoc-RQKWFQNR

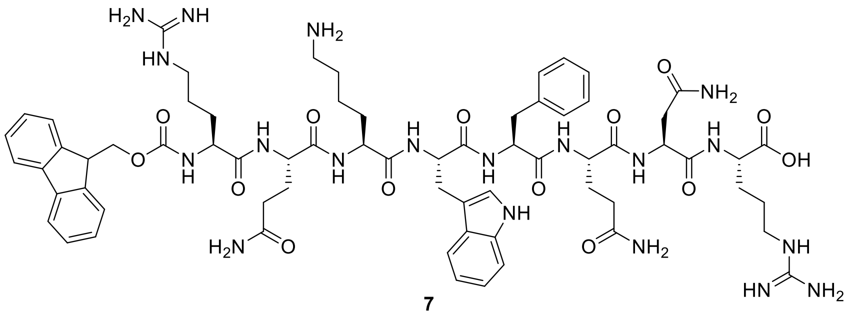

7

Fmoc-RQKWFQNR **7** was synthesized using SPPS at a scale of 0.10 mmol on Fmoc-Arg(Pbf)-*Wang*-resin by coupling of Fmoc-Arg(Pbf)-OH (0.2 M in 6 mL DMF), Fmoc-Gln(Trt)-OH (0.2 M in 6 mL DMF), Fmoc-Lys(Boc)-OH (0.2 M in 3 mL DMF), Fmoc-Trp(Boc)-OH (0.2 M in 3 mL DMF), Fmoc-Phe-OH (0.2 M in 3 mL DMF) and Fmoc-Asn(Trt)-OH (0.2 M in 3 mL DMF) according to the general procedure for automated peptide synthesis. After final coupling, the resin was washed with DMF (3 x 5 mL), DCM (3 x 5 mL) and treated with a cleavage cocktail consisting of 95 % TFA, 2.5 % TIPS and 2.5 % H_2O for three hours. The mixture was filtered and washed with TFA (1 mL). The crude product was precipitated from cold ether (60 mL), centrifuged (4000 rpm, 0 °C, 20 min) and the supernatant was decanted. Upon drying, a

colorless solid crude was obtained. The crude was purified using HPLC according to the general procedure. The product eluted after 12.0 min. Fmoc-RQKWFQNR **7** (52 mg; 0.032 mmol; 32 %) was obtained as a colorless solid (three attached TFA ions estimated).

LC-MS: (pos.): calculated for $[M + 2 H]^{2+}$: 693.2, measured 693.4; calculated for $[M + H]^{+}$: 1385.6, measured 1385.9.

High-Res-ESI: (pos.): calculated for $C_{67}H_{91}N_{19}O_{14}^{2+}$ $[M + 2 H]^{2+}$: 692.8491, measured: 692.8545.

MALDI-Tof: calculated for $[M + H]^{+}$: 1385.6, measured: 1384.8 $[M + H]^{+}$.

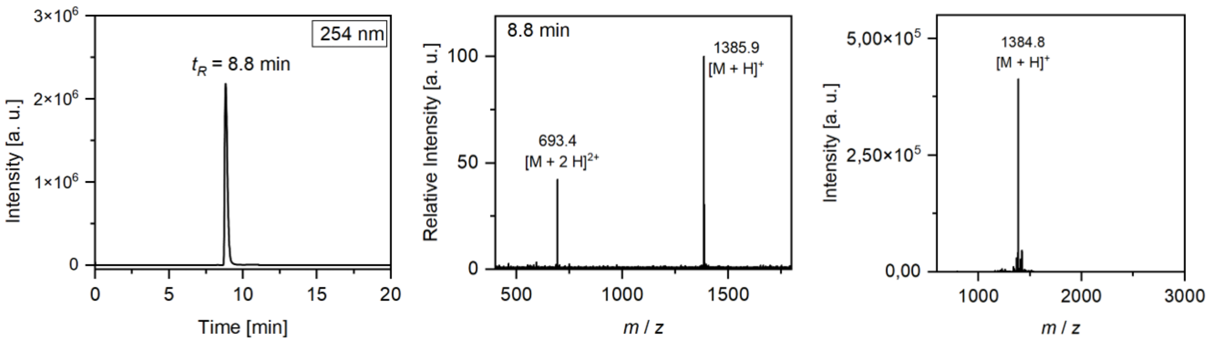

Figure S9: LC elugram (left, detection at 254 nm), corresponding mass data (middle) and MALDI-Tof-MS spectrum (right) of Fmoc-RQKWFQNR **7**.

2.2.9 Synthesis of RQIKIWFQNR

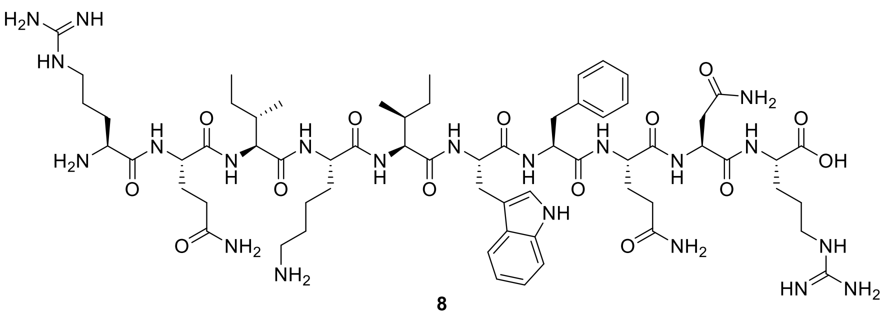

8

RQIKIWFQNR **8** was synthesized using SPPS at a scale of 0.10 mmol on Fmoc-Arg(Pbf)-*Wang*-resin by coupling of Fmoc-Arg(Pbf)-OH (0.2 M in 6 mL DMF), Fmoc-Gln(Trt)-OH (0.2 M in 6 mL DMF), Fmoc-Ile-OH (0.2 M in 6 mL DMF), Fmoc-Lys(Boc)-OH (0.2 M in 3 mL DMF), Fmoc-Trp(Boc)-OH (0.2 M in 3 mL DMF), Fmoc-Phe-OH (0.2 M in 3 mL DMF) and Fmoc-Asn(Trt)-OH (0.2 M in 3 mL DMF) according to the general procedure for automated peptide synthesis. After final deprotection, the resin was washed with DMF (3 x 5 mL) and DCM (3 x 5 mL). The resin was treated with a cleavage cocktail consisting of 95 % TFA, 2.5 % TIPS and 2.5 % H_2O for three hours. The resin was filtered and washed with TFA (1 mL).

The crude product was precipitated from cold ether (60 mL), centrifuged (4000 rpm, 0 °C, 20 min) and the supernatant was decanted. Upon drying, a colorless solid crude was obtained. The crude was purified using HPLC according to the general procedure. The product eluted after 7.00 min and RQIKIWFQNR **8** (27 mg; 0.019 mmol; 19 %) was obtained as a colorless solid (three attached TFA ions estimated).

LC-MS: (pos.): calculated for $[M + 2 H]^{2+}$: 694.8, measured: 695.4; calculated for $[M + H]^{+}$: 1389.7, measured: 1390.0.

High-Res-ESI: (pos.): calculated for $C_{64}H_{104}N_{21}O_{14}^{3+}$ $[M + 3 H]^{3+}$: 463.6019, measured: 463.6028; calculated for $C_{64}H_{103}N_{21}O_{14}^{2+}$ $[M + 2 H]^{2+}$: 694.8991, measured: 694.8974.

MALDI-Tof: calculated for $[M + H]^{+}$: 1389.7, measured: 1388.9.

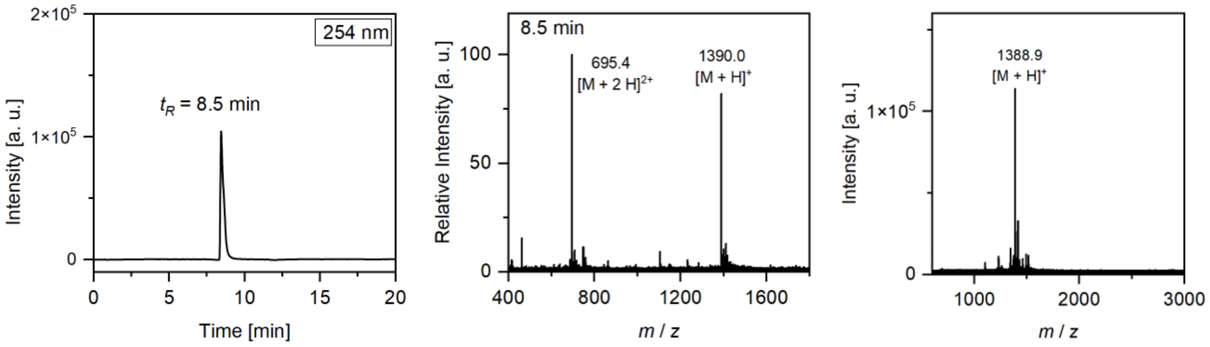

Figure S10: LC elugram (left, detection at 254 nm), corresponding mass data (middle) and MALDI-Tof-MS spectrum (right) of RQIKIWFQNR **8**.

2.2.10 Synthesis of NBD-RQIKIWFQNR

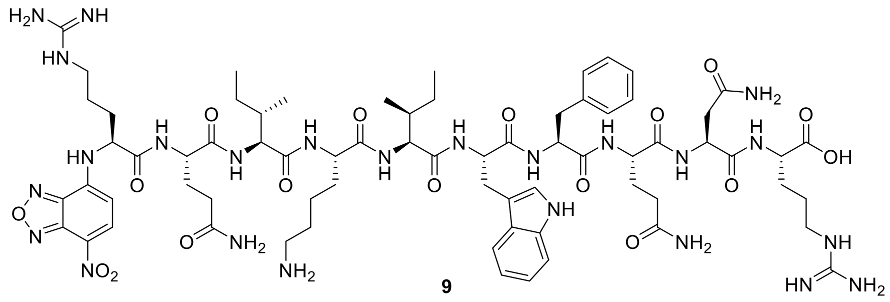

9

NBD-RQIKIWFQNR **9** was synthesized using SPPS at a scale of 0.10 mmol on Fmoc-Arg(Pbf)-*Wang*-resin by coupling of Fmoc-Arg(Pbf)-OH (0.2 M in 6 mL DMF), Fmoc-Gln(Trt)-OH (0.2 M in 6 mL DMF), Fmoc-Ile-OH (0.2 M in 6 mL DMF), Fmoc-Lys(Boc)-OH (0.2 M in 3 mL DMF), Fmoc-Trp(Boc)-OH (0.2 M in 3 mL DMF), Fmoc-Phe-OH (0.2 M in 3 mL DMF) and Fmoc-Asn(Trt)-OH (0.2 M in 3 mL DMF) according to the general procedure for automated peptide synthesis.

After final deprotection, the resin was washed with DMF (3 x 5 mL). 4-Chloro-7-nitrobenzofurazan (60 mg, 0.30 mmol, 3.0 eq.) and *N, N'*-DIPEA (880 µL, 0.50 mmol, 5.0 eq.) were added to the resin in DMF (5 mL) and stirred overnight in the dark at room temperature. The resin was washed with DMF (3 x 5 mL) and DCM (3 x 5 mL) and treated with a cleavage cocktail consisting of 95 % TFA, 2.5 % TIPS and 2.5 % H_2O for three hours. The resin was filtered and washed with TFA (1 mL). The crude product was precipitated from cold ether (60 mL), centrifuged (4000 rpm, 0 °C, 20 min) and the supernatant was decanted. Upon drying, an orange solid crude was obtained. The crude was purified using HPLC according to the general procedure. The product eluted after 7.50 min and NBD-RQIKIWFQNR **9** (25 mg; 0.016 mmol; 16 %) was obtained as an orange solid (three attached TFA ions estimated).

LC-MS: (pos.): calculated for $[M + 2 H]^{2+}$: 776.4, measured: 776.8; calculated for $[M + H]^{+}$: 1551.8, measured: 1552.8.

High-Res-ESI: (pos.): calculated for $C_{70}H_{105}N_{24}O_{17}^{3+}$ $[M + 3 H]^{3+}$: 517.9358, measured: 517.9580; calculated for $C_{70}H_{104}N_{24}O_{17}^{2+}$ $[M + 2 H]^{2+}$: 776.4000, measured: 776.9350; $C_{70}H_{103}N_{24}O_{17}^{+}$ $[M + H]^{+}$: 1551.7928, measured: 1551.8630.

MALDI-Tof: calculated for $[M + H]^{+}$: 1551.8, measured: 1551.9.

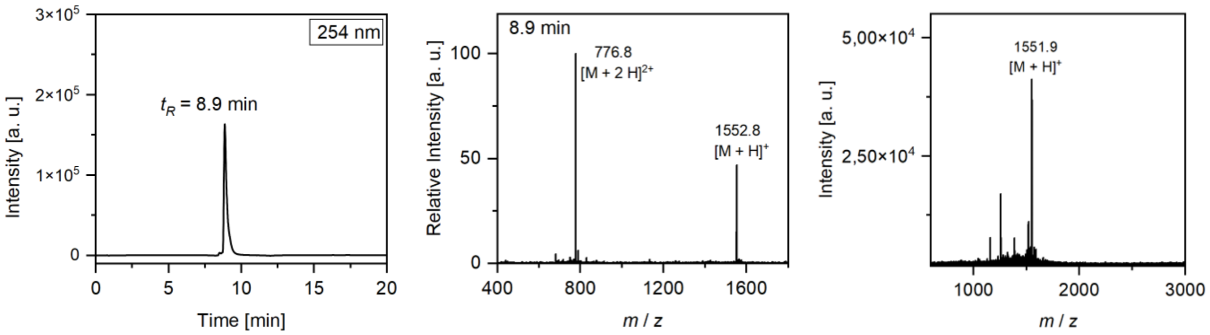

Figure S11: LC elugram (left, detection at 254 nm), corresponding mass data (middle) and MALDI-Tof-MS spectrum (right) of NBD-RQIKIWFQNR **9**.

2.2.11 Synthesis of Fmoc-RQIEIWFQNR

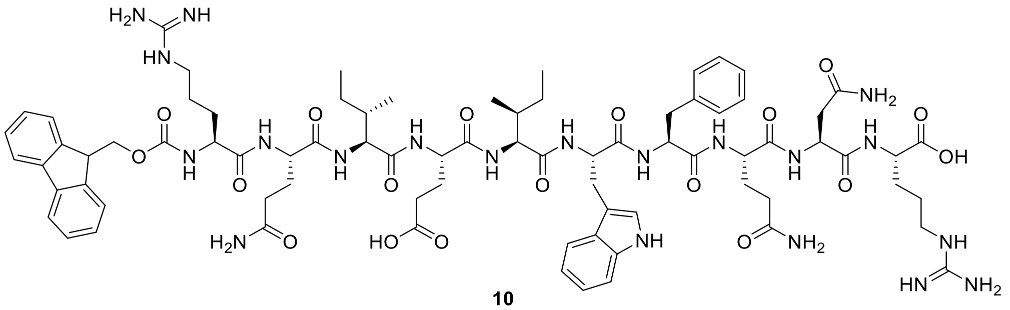

10

Fmoc-RQIEIWFQNR **10** was synthesized using SPPS at a scale of 0.10 mmol on Fmoc-Arg(Pbf)-*Wang*-resin by coupling of Fmoc-Arg(Pbf)-OH (0.2 M in 6 mL DMF), Fmoc-Gln(Trt)-OH (0.2 M in 6 mL DMF), Fmoc-Ile-OH (0.2 M in 6 mL DMF), Fmoc-Glu(O^{t}Bu)-OH (0.2 M in 3 mL DMF), Fmoc-Trp(Boc)-OH (0.2 M in 3 mL DMF), Fmoc-Phe-OH (0.2 M in 3 mL DMF) and Fmoc-Asn(Trt)-OH (0.2 M in 3 mL DMF) according to the general procedure for automated peptide synthesis. The resin was washed with DMF (3 x 5 mL) and DCM (3 x 5 mL). The resin was treated with a cleavage cocktail consisting of 95 % TFA, 2.5 % TIPS and 2.5 % H_2O for three hours. The mixture was filtered and washed with TFA (1 mL). Then, crude product was precipitated from cold ether (60 mL), centrifuged (4000 rpm, 0 °C, 20 min) and the supernatant was decanted. Upon drying, a colorless solid crude was obtained. The crude was purified using HPLC according to the general procedure. The product eluted after 9.50 min. Fmoc-RQIEIWFQNR **10** (40 mg; 0.022 mmol; 22 %) was obtained as a colorless solid (two attached TFA ions estimated).

LC-MS: (pos.): calculated for $[M + 2 H]^{2+}$: 806.7, measured: 807.0; calculated for $[M + H]^{+}$: 1612.8, measured: 1613.1.

High-Res-ESI: (pos.): calculated for $C_{78}H_{109}N_{20}O_{18}^{3+}$ $[M + 3 H]^{3+}$: 537.9404, measured: 537.9457; calculated for $C_{78}H_{108}N_{20}O_{18}^{2+}$ $[M + 2 H]^{2+}$: 806.4070, measured: 806.4130.

MALDI-Tof: calculated for $[M + H]^{+}$: 1612.8, measured: 1611.9.

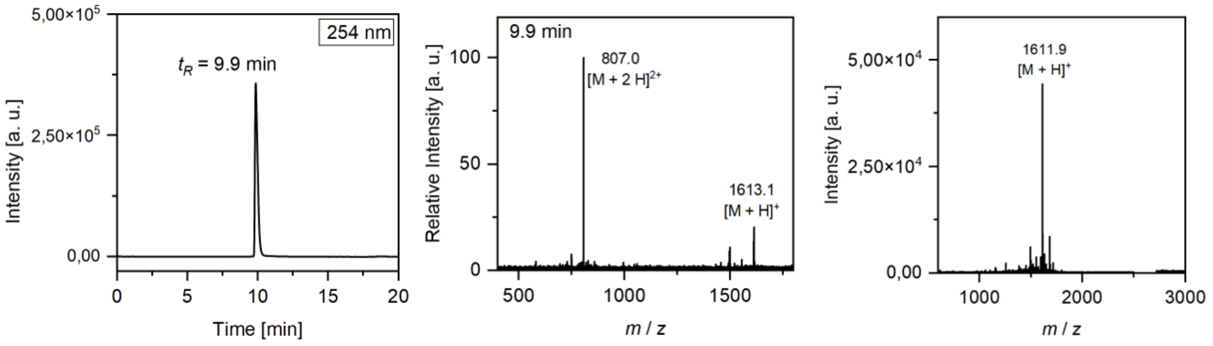

Figure S12: LC elugram (left, detection at 254 nm), corresponding mass data (middle) and MALDI-Tof-MS spectrum (right) of Fmoc-RQIEIWFQNR **10**.

2.2.12 Synthesis of Fmoc-RQISIWFQNR

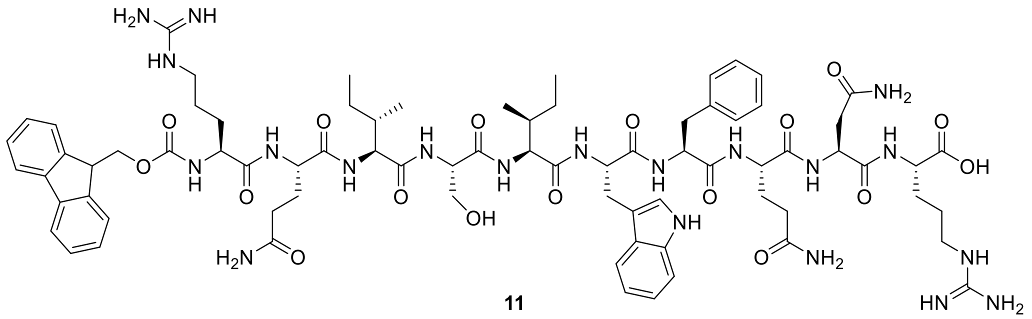

11

Fmoc-RQISIWFQNR **11** was synthesized using SPPS at a scale of 0.10 mmol on Fmoc-Arg(Pbf)-*Wang*-resin by coupling of Fmoc-Arg(Pbf)-OH (0.2 M in 6 mL DMF), Fmoc-Gln(Trt)-OH (0.2 M in 6 mL DMF), Fmoc-Ile-OH (0.2 M in 3 mL DMF), Fmoc-Ser(^{t}Bu)-OH (0.2 M in 3 mL DMF), Fmoc-Trp(Boc)-OH (0.2 M in 3 mL DMF), Fmoc-Phe-OH (0.2 M in 3 mL DMF) and Fmoc-Asn(Trt)-OH (0.2 M in 3 mL DMF) according to the general procedure for automated peptide synthesis. After the final coupling, the resin was washed with DMF (3 x 5 mL) and DCM (3 x 5 mL). The resin was treated with a cleavage cocktail consisting of 95 % TFA, 2.5 % TIPS and 2.5 % H_2O for three hours. The resin was filtered and washed with TFA (1 mL). The crude product was precipitated from cold ether (60 mL), centrifuged (4000 rpm, 0 °C, 20 min) and the supernatant was decanted. Upon drying, a colorless solid crude was obtained. The crude was purified using HPLC according to the general procedure and the product eluted after 9.00 min. Fmoc-RQISIWFQNR **11** (17 mg; 0.010 mmol; 10 %) was obtained as a colorless solid (two attached TFA ions estimated).

LC-MS: (pos.): calculated for $[M + 2 H]^{2+}$: 785.9, measured: 785.9; calculated for $[M + H]^+$: 1570.8, measured: 1570.7.

High-Res-ESI: (pos.): calculated for $C_{76}H_{106}N_{20}O_{17}^{2+}$ $[M + 2 H]^{2+}$: 785.4017, measured: 785.6332.

MALDI-Tof: calculated for $[M + H]^+$: 1570.8, measured: 1570.7 $[M + H]^+$.

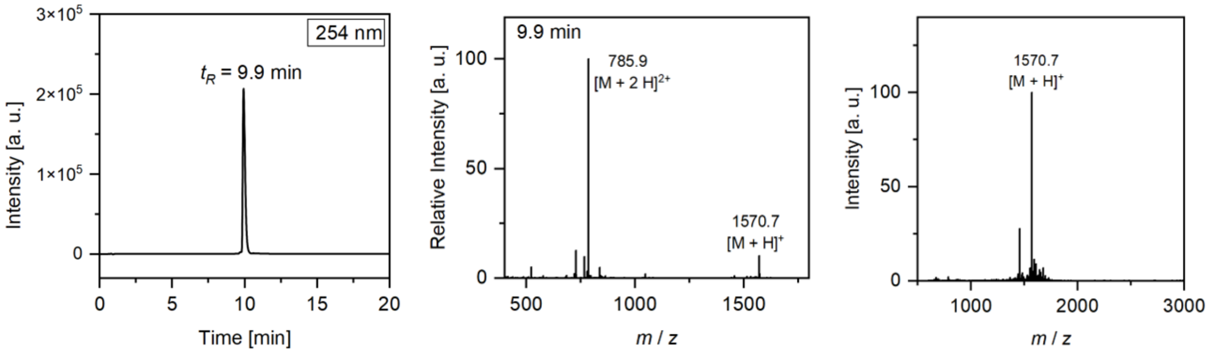

Figure S13: LC elugram (left, detection at 254 nm), corresponding mass data (middle) and MALDI-Tof-MS spectrum (right) of Fmoc-RQISIWFQNR **11**.

2.2.13 Synthesis of Fmoc-RQILIWFQNR

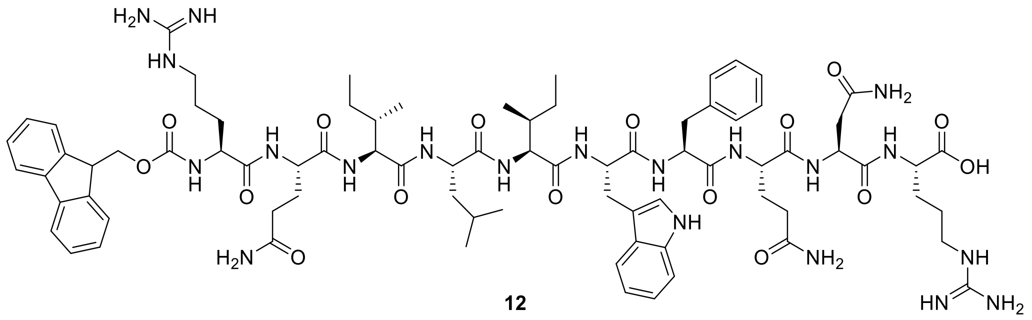

Fmoc-RQILIWFQNR **12** was synthesized using SPPS at a scale of 0.10 mmol on Fmoc-Arg(Pbf)-*Wang*-resin by coupling of Fmoc-Arg(Pbf)-OH (0.2 M in 6 mL DMF), Fmoc-Gln(Trt)-OH (0.2 M in 6 mL DMF), Fmoc-Ile-OH (0.2 M in 3 mL DMF), Fmoc-Leu-OH (0.2 M in 3 mL DMF), Fmoc-Trp(Boc)-OH (0.2 M in 3 mL DMF), Fmoc-Phe-OH (0.2 M in 3 mL DMF) and Fmoc-Asn(Trt)-OH (0.2 M in 3 mL DMF) according to the general procedure for automated peptide synthesis. After the final coupling, the resin was washed with DMF (3 x 5 mL) and DCM (3 x 5 mL). The resin was treated with a cleavage cocktail consisting of 95 % TFA, 2.5 % TIPS and 2.5 % H_2O for three hours. The resin was filtered and washed with TFA (1 mL). The crude product was precipitated from cold ether (60 mL), centrifuged (4000 rpm, 0 °C, 20 min) and the supernatant was decanted. Upon drying, a colorless solid crude was obtained. The crude was purified using HPLC according to the general procedure and the product eluted after 9.60 min. Fmoc-RQILIWFQNR **12** (34 mg; 0.019 mmol; 19 %) was obtained as a colorless solid (two attached TFA ions estimated).

LC-MS: (pos.): calculated for $[M + 2 H]^{2+}$: 798.4, measured: 798.9; calculated for $[M + H]^{+}$: 1595.8, measured: 1597.0.

High-Res-ESI: (pos.): calculated for $C_{79}H_{112}N_{20}O_{16}^{2+}$ $[M + 2 H]^{2+}$: 798.4277, measured: 798.9369.

MALDI-Tof: calculated for $[M + H]^{+}$: 1595.8, measured: 1595.7 $[M + H]^{+}$.

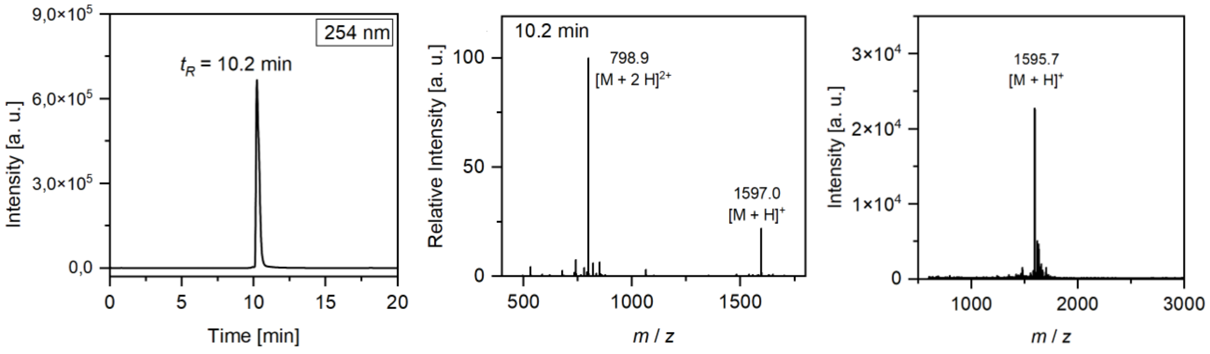

Figure S14: LC elugram (left, detection at 254 nm), corresponding mass data (middle) and MALDI-Tof-MS spectrum (right) of Fmoc-RQILIWFQNR **12**.

2.2.14 Synthesis of Fmoc-RQIHIWFQNR

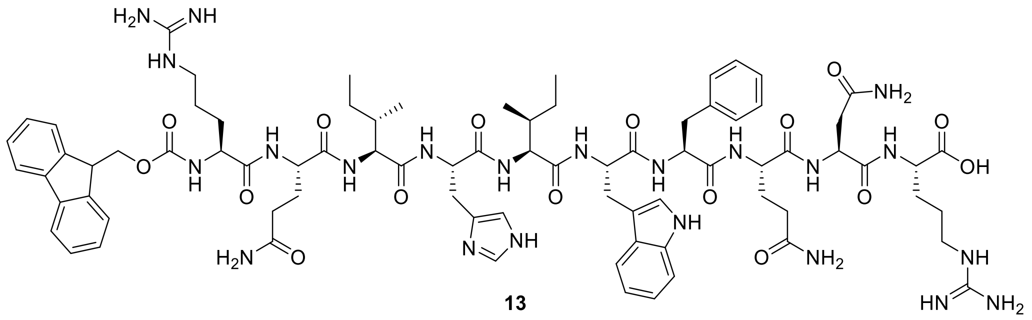

13

Fmoc-RQIHIWFQNR **13** was synthesized using SPPS at a scale of 0.25 mmol on Fmoc-Arg(Pbf)-*Wang*-resin by coupling of Fmoc-Arg(Pbf)-OH (0.2 M in 4 mL DMF), Fmoc-Gln(Trt)-OH (0.2 M in 8 mL DMF), Fmoc-Ile-OH (0.2 M in 8 mL DMF), Fmoc-His(Trt)-OH (0.2 M in 4 mL DMF), Fmoc-Trp(Boc)-OH (0.2 M in 4 mL DMF), Fmoc-Phe-OH (0.2 M in 4 mL DMF) and Fmoc-Asn(Trt)-OH (0.2 M in 8 mL DMF) according to the general procedure for manual peptide synthesis. After the final coupling, the resin was washed with DMF (3 x 5 mL) and DCM (3 x 5 mL). The resin was treated with a cleavage cocktail consisting of 95 % TFA, 2.5 % TIPS and 2.5 % H_2O for three hours. The resin was filtered and washed with TFA (1 mL). The crude product was precipitated from cold ether (60 mL), centrifuged (4000 rpm, 0 °C, 20 min) and the supernatant was decanted. Upon drying, a colorless solid crude was obtained. The crude was purified using HPLC according to the general procedure. The product eluted after 8.50 min.

Fmoc-RQIHIWFQNR **13** (115 mg; 0.059 mmol; 24 %) was obtained as a colorless solid (three attached TFA ions estimated).

LC-MS: (pos.): calculated for $[M + 3 H]^{3+}$: 540.6, measured: 540.9; calculated for $[M + 2 H]^{2+}$: 810.4, measured: 810.9; calculated for $[M + H]^{+}$: 1619.8, measured: 1621.0.

High-Res-ESI: (pos.): calculated for $C_{79}H_{109}N_{22}O_{16}^{3+}$ $[M + 3 H]^{3+}$: 540.6125, measured: 540.9665; calculated for $C_{79}H_{108}N_{22}O_{16}^{2+}$ $[M + 2 H]^{2+}$: 810.4152, measured: 810.9453; calculated for $C_{79}H_{107}N_{22}O_{16}^{+}$ $[M + H]^{+}$: 1619.8230, measured: 1619.8910.

MALDI-Tof: calculated for [M + H]$^+$: 1619.8, measured: 1620.9 [M + H]$^+$.

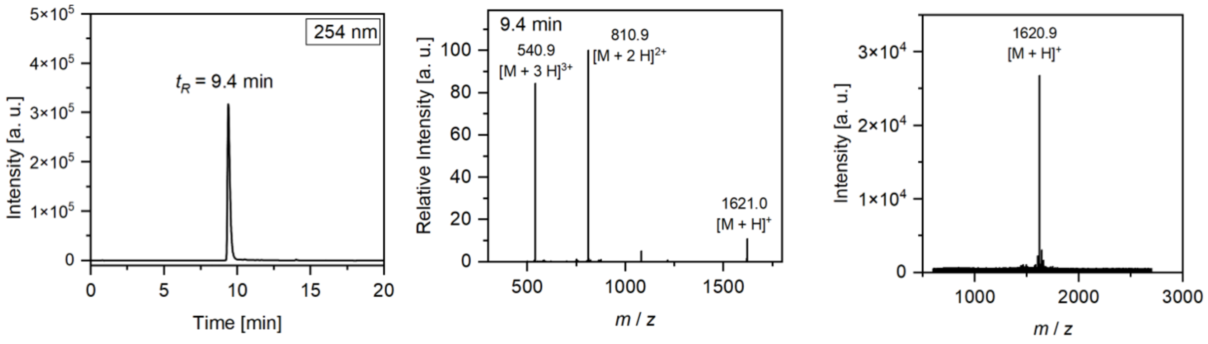

Figure S15: LC elugram (left, detection at 254 nm), corresponding mass data (middle) and MALDI-Tof-MS spectrum (right) of Fmoc-RQIHIWFQNR **13**.

2.2.15 Synthesis of TAMRA-RQIKIWFQNR

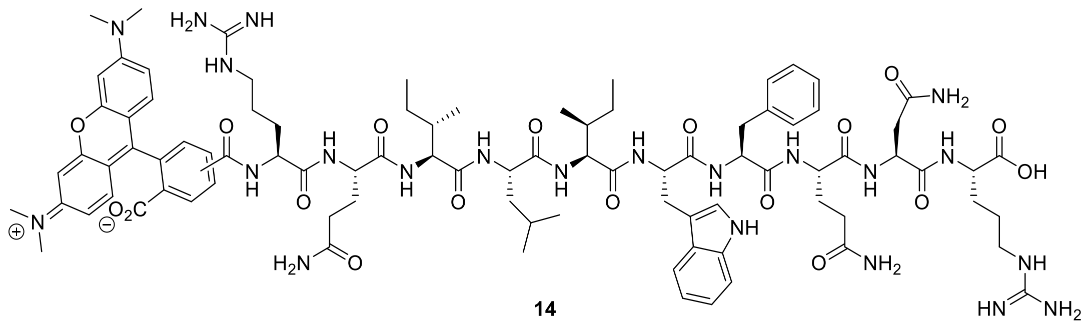

14

TAMRA-RQIKIWFQNR **14** was synthesized using SPPS at a scale of 0.10 mmol on Fmoc-Arg(Pbf)-*Wang*-resin by coupling of Fmoc-Arg(Pbf)-OH (0.2 M in 1 mL DMF), Fmoc-Gln(Trt)-OH (0.2 M in 2 mL DMF), Fmoc-Ile-OH (0.2 M in 2 mL DMF), Fmoc-Lys(Boc)-OH (0.2 M in 1 mL DMF), Fmoc-Trp(Boc)-OH (0.2 M in 1 mL DMF), Fmoc-Phe-OH (0.2 M in 1 mL DMF) and Fmoc-Asn(Trt)-OH (0.2 M in 1 mL DMF) according to the general procedure for manual peptide synthesis. After final deprotection, the resin was washed with DMF (3 x 5 mL). 5-(and 6)-Carboxytetramethylhodamine (TAMRA) *N*-hydroxysuccinimidyl ester (60 mg, 0.11 mmol, 1.1 eq.) and *N, N'*-DIPEA (800 µL, 0.50 mmol, 5.0 eq.) were added to the resin in DMF (5 mL) and stirred overnight in the dark at room temperature. The resin was washed with DMF (3 x 5 mL) and DCM (3 x 5 mL) and treated with a cleavage cocktail consisting of 95 % TFA, 2.5 % TIPS and 2.5 % H_2O for three hours. The resin was filtered and washed with TFA (1 mL). The crude product was precipitated from cold ether (60 mL), centrifuged (4000 rpm, 0 °C, 20 min) and the supernatant was decanted. Upon drying, a purple solid crude was obtained. The crude was purified using HPLC according to the general procedure. The product eluted after 6.70 min and TAMRA-RQIKIWFQNR **14** (100 mg; 0.044 mmol; 44 %) was obtained as a purple solid (three attached TFA ions estimated).

LC-MS: (pos.): calculated for $[M + 4 H]^{4+}$: 451.0, measured: 451.3; calculated for $[M + 3 H]^{3+}$: 601.0, measured: 600.9; calculated for $[M + 2 H]^{2+}$: 901.0, measured: 901.1.

High-Res-ESI: (pos.): calculated for $C_{89}H_{125}N_{23}O_{18}^{4+}$ $[M + 4 H]^{4+}$: 450.9888, measured: 451.2504; calculated for $C_{89}H_{124}N_{23}O_{18}^{3+}$ $[M + 3 H]^{3+}$: 600.9826, measured: 601.3415; calculated for $C_{89}H_{123}N_{23}O_{18}^{2+}$ $[M + 2 H]^{2+}$: 900.9703, measured: 901.5018.

MALDI-Tof: calculated for $[M + H]^{+}$: 1800.9, measured: 1801.9 $[M + H]^{+}$.

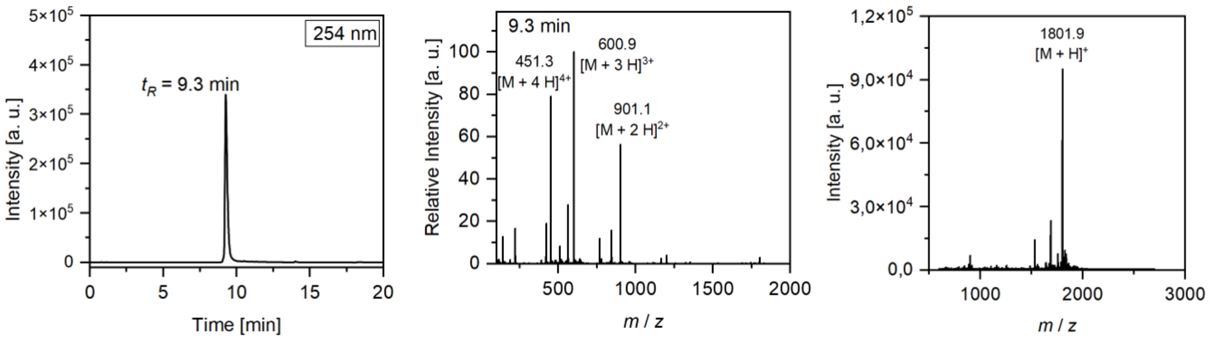

Figure S16: LC elugram (left, detection at 254 nm), corresponding mass data (middle) and MALDI-Tof-MS spectrum (right) of TAMRA-RQIKIWFQNR **14**.

3 Self-Assembly and Catalytic Activity

3.1 Self-Assembly

3.1.1 Assembly Conversion and Critical Aggregation Concentration

The peptides were analyzed according to the general procedure for Assembly Conversion determination. The elugrams were referenced to the standard and plotted using *Origin Pro* by *OriginLab*®. The Area under Curve (AUC) of the peptide signals in PBS (AUC_{PBS}) and in MeOH (AUC_{MeOH}) were calculated and used to determine the conversion rate (CR) using the following equation:

$$CR\ [\%] = (1 - \frac{AUC_{PBS}}{AUC_{MeOH}}) \cdot 100\ \%$$

For peptides with complete assembly conversion rate, CR > 95 % was chosen due to the detection limit of the analytical HPLC.

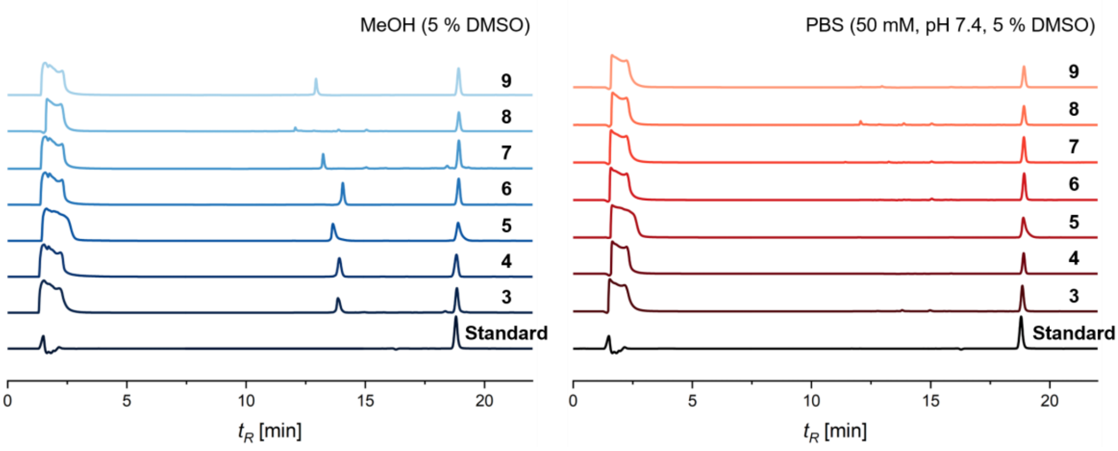

Figure S17: Elugrams of Fmoc-RQIKIWFQNR **3**, Fmoc-RQIRIWFQNR **4**, Fmoc-RQI*Dap*IWFQNR **5**, Fmoc-RQFKFWFQNR **6**, Fmoc-RQKWFQNR **7**, RQIKIWFQNR **8** and NBD-RQIKIWFQNR **9** (all 75 µM) in MeOH (5 % DMSO; *left*) and PBS (50 mM, pH 7.4, 5 % DMSO; *right*) and the internal standard Fmoc-Trp(Boc)-OH (100 µM in MeOH, **Standard**). The elugrams are shown as a plot of absorbance intensity at 214 nm against retention time t_R.

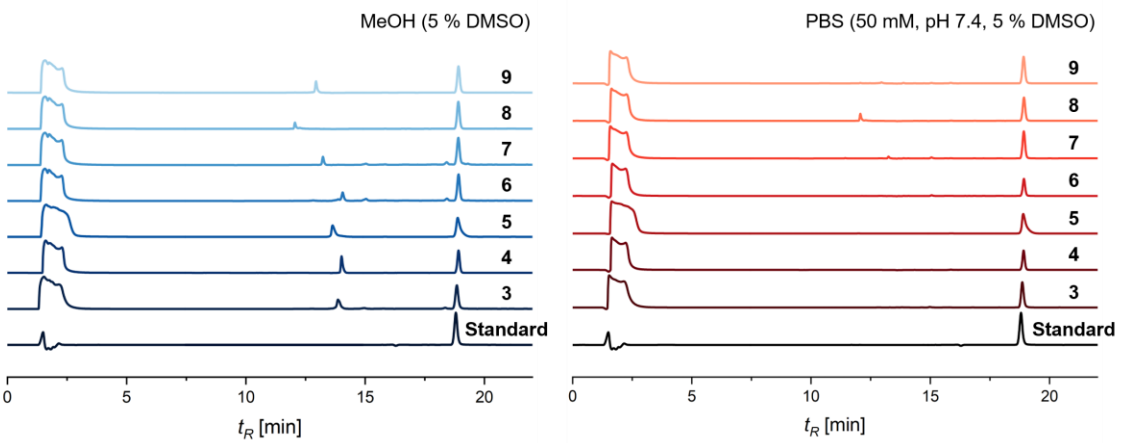

Figure S18: Elugrams of Fmoc-RQIKIWFQNR **3**, Fmoc-RQIRIWFQNR **4**, Fmoc-RQI*Dap*IWFQNR **5**, Fmoc-RQFKFWFQNR **6**, Fmoc-RQKWFQNR **7**, RQIKIWFQNR **8** and NBD-RQIKIWFQNR **9** (all 50 µM) in MeOH (5 % DMSO; *left*) and PBS (50 mM, pH 7.4, 5 % DMSO; *right*) and the internal standard Fmoc-Trp(Boc)-OH (100 µM in MeOH, **Standard**). The elugrams are shown as a plot of absorbance intensity at 214 nm against retention time t_R.

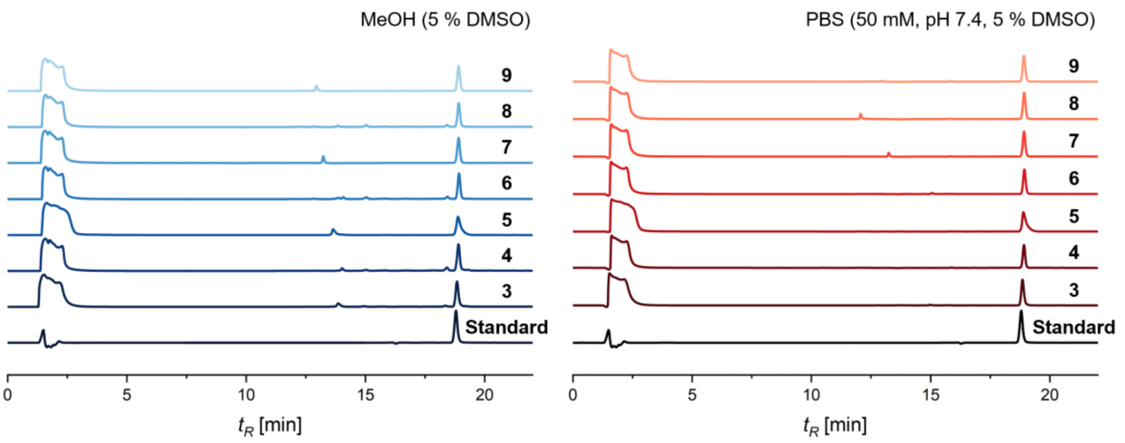

Figure S19: Elugrams of Fmoc-RQIKIWFQNR **3**, Fmoc-RQIRIWFQNR **4**, Fmoc-RQI*Dap*IWFQNR **5**, Fmoc-RQFKFWFQNR **6**, Fmoc-RQKWFQNR **7**, RQIKIWFQNR **8** and NBD-RQIKIWFQNR **9** (all 25 µM) in MeOH (5 % DMSO; *left*) and PBS (50 mM, pH 7.4, 5 % DMSO; *right*) and the internal standard Fmoc-Trp(Boc)-OH (100 µM in MeOH, **Standard**). The elugrams are shown as a plot of absorbance intensity at 214 nm against retention time t_R.

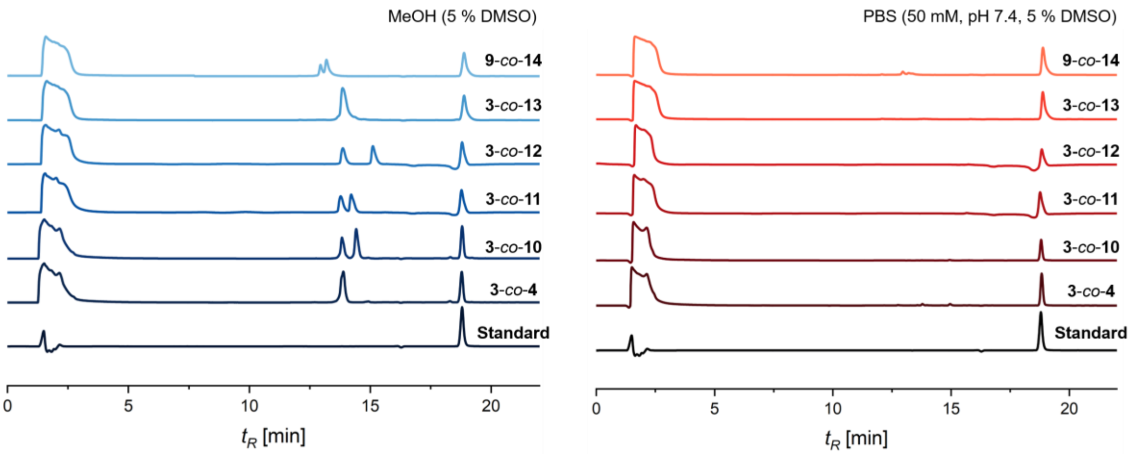

Figure S20: Elugrams of the co-assemblies **3**-*co*-**4**, **3**-*co*-**10**, **3**-*co*-**11**, **3**-*co*-**12**, **3**-*co*-**13** and **9**-*co*-**14** (1:1; 100 µM each component) in MeOH (5 % DMSO; *left*) and PBS (50 mM, pH 7.4, 5 % DMSO; *right*) as well as the internal standard Fmoc-Trp(Boc)-OH (100 µM in MeOH, **Standard**). The elugrams are shown as a plot of absorbance intensity at 214 nm against retention time t_R.

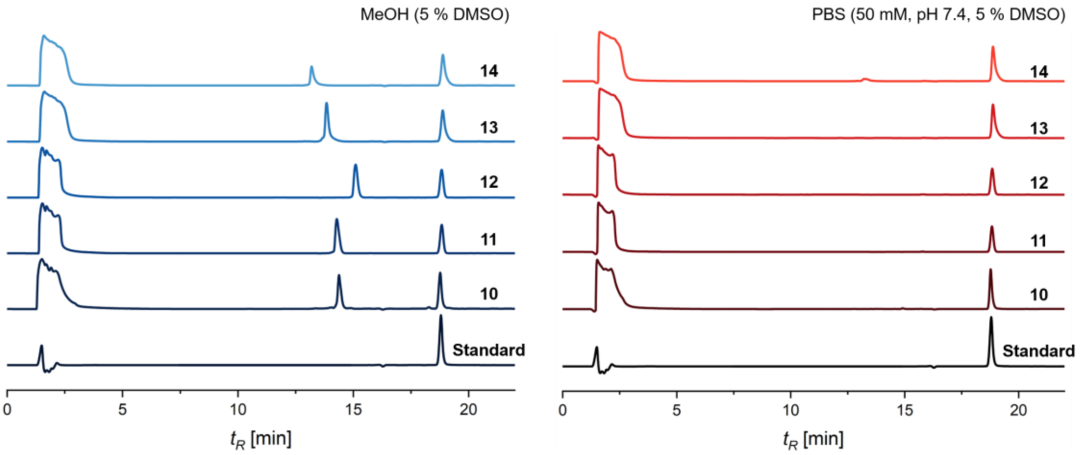

Figure S21: Elugrams of Fmoc-RQIEIWFQNR **10**, Fmoc-RQISIWFQNR **11**, Fmoc-RQILIWFQNR **12**, Fmoc-RQIHIWFQNR **13** (all 100 µM) and TAMRA-RQIKIWFQNR **14** (50 µM) in MeOH (5 % DMSO; *left*) and PBS (50 mM, pH 7.4, 5 % DMSO; *right*) as well as the internal standard Fmoc-Trp(Boc)-OH (100 µM in MeOH, **Standard**). The elugrams are shown as a plot of absorbance intensity at 214 nm against retention time t_R.

3.1.2 Proteostat Aggregation Assay

The experiments were performed according to the general procedure for Proteostat® Aggregation Assay. The fluorescence intensity was measured at λ_{ex} = 550 nm and λ_{em} = 600 nm.

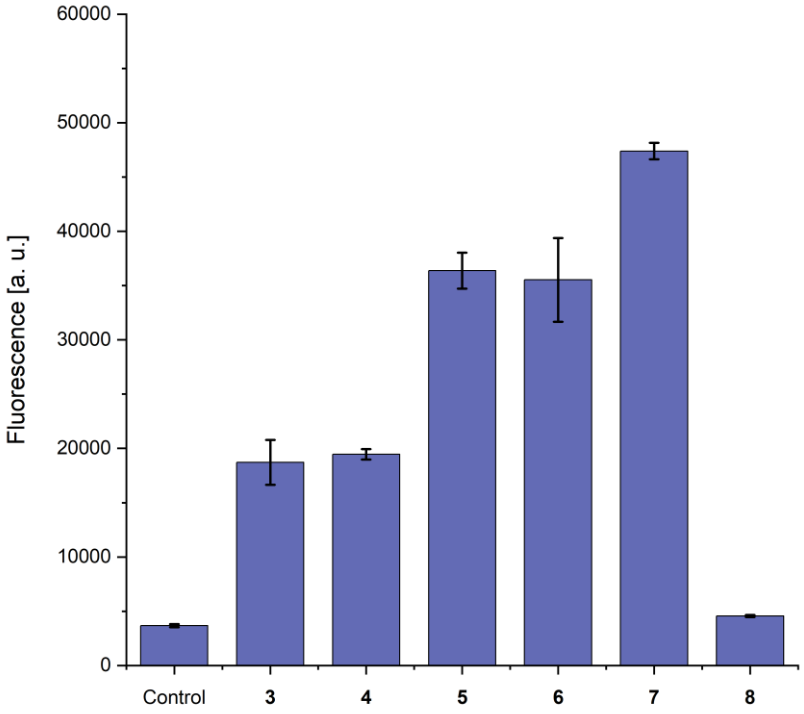

Figure S22: Proteostat® Aggregation Assay fluorescence bar chart of the peptides Fmoc-RQIKIWFQNR **3**, Fmoc-RQIRIWFQNR **4**, Fmoc-RQI*Dap*IWFQNR **5**, Fmoc-RQFKFWFQNR **6**, Fmoc-RQKWFQNR **7** and RQIKIWFQNR **8** (all 100 µM) in PBS (50 mM, pH 7.4, 5 % DMSO). The samples were incubated at room temperature for 24 h prior to analysis. The fluorescence intensity at λ_{ex} = 550 nm and λ_{em} = 600 nm is shown. NBD-RQIKIWFQNR **9** was not analyzed due to interfering fluorescence spectra.

3.1.3 Fourier-Transform Infrared-Spectroscopy (FTIR)

The peptides were analyzed according to the general procedure for FTIR-measurements. The FTIR-spectrum of PBS (50 mM, pH 7.4, 5 % DMSO) was taken as a reference spectrum and subtracted from subsequently acquired peptide-spectra. The signals were processed following a general procedure presented by Dong *et al*.[29] The 2nd derivative of absorbance was calculated and the signals were smoothened using the Savizky-Golay filter (20 point). The baseline was corrected in the region of 1600 – 1700 cm^{-1} and the data was fitted using Gaussian fits.

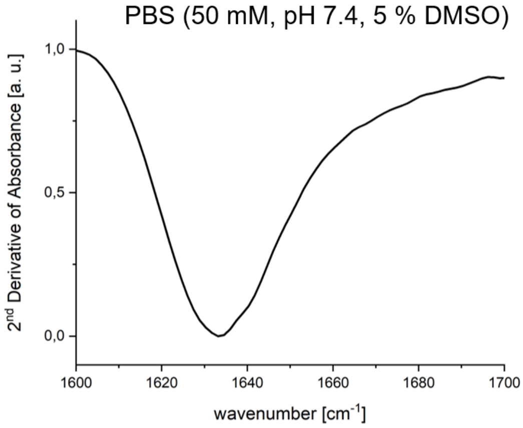

Figure S23: FTIR-spectrum of PBS (50 mM, pH 7.4, 5 % DMSO) in the range of 1600 – 1700 cm^{-1} after processing. The 2nd derivative of absorbance is plotted against the wavenumber.

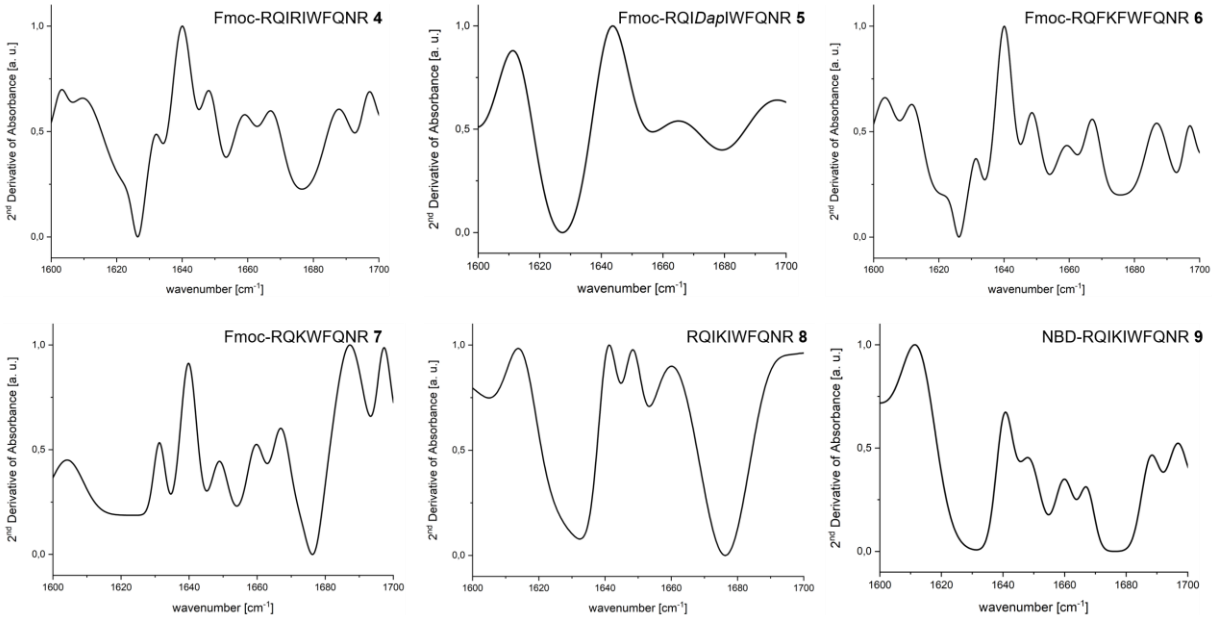

Figure S24: FTIR-spectra of Fmoc-RQIRIWFQNR **4**, Fmoc-RQI*Dap*IWFQNR **5**, Fmoc-RQFKFWFQNR **6**, Fmoc-RQKWFQNR **7**, RQIKIWFQNR **8** and NBD-RQIKIWFQNR **9** (all 1 mM) in PBS (50 mM, pH 7.4, 5 % DMSO) in the range of 1600 – 1700 cm^{-1} after reference spectrum subtraction and processing. The 2nd derivative of absorbance is plotted against the wavenumber.

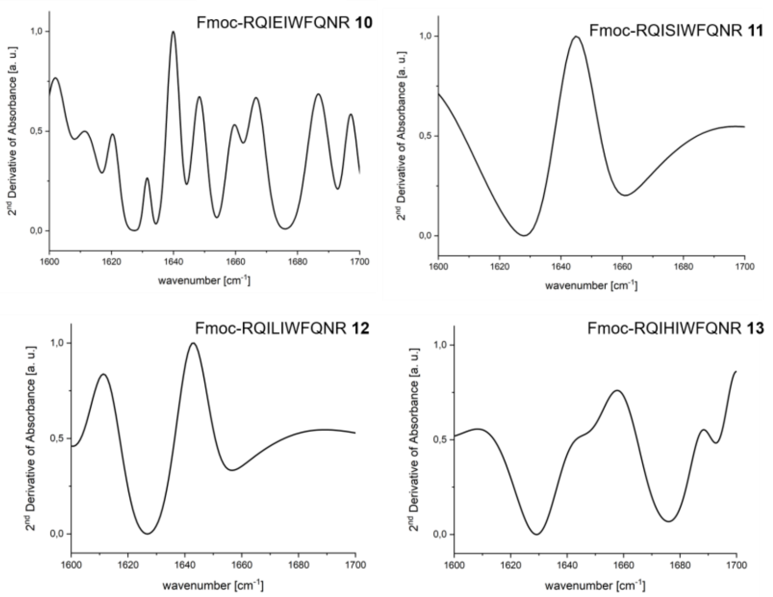

Figure S25: FTIR-spectra of Fmoc-RQIEIWFQNR **10**, Fmoc-RQISIWFQNR **11**, Fmoc-RQILIWFQNR **12** and Fmoc-RQIHIWFQNR **13** (all 1 mM) in PBS (50 mM, pH 7.4, 5 % DMSO) in the range of 1600 – 1700 cm^{-1} after reference spectrum subtraction and processing. The 2nd derivative of absorbance is plotted against the wavenumber.

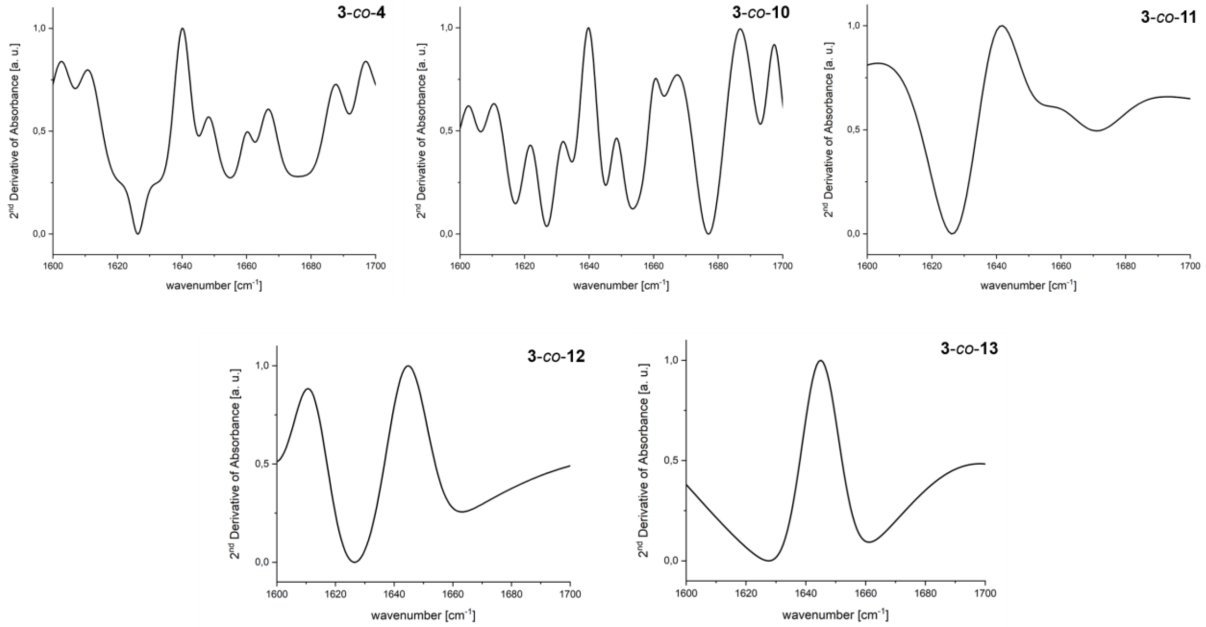

Figure S26: FTIR-spectra of co-assemblies **3**-*co*-**4**, **3**-*co*-**10**, **3**-*co*-**11**, **3**-*co*-**12** and **3**-*co*-**13** (all 2 mM, 1:1) in PBS buffer (50 mM, pH 7.4, 5 % DMSO) in the range of 1600 – 1700 cm^{-1} after reference spectrum subtraction and processing. The 2nd derivative of absorbance is plotted against the wavenumber.

3.1.4 Circular Dichroism-Spectroscopy (CD)

The CD-spectrum was acquired according to the general procedure on a *JASCO* J-1500 spectrometer in a 1 mm High Precision Cell by *Hellma Analytics*. Fmoc-RQIKIWFQNR **3** was prepared in PBS buffer (50 mM, pH 7.4) with a concentration of 100 µM and 5 % 2,2,2-trifluoroethanol (TFE). The peptide was incubated for 24 h at room temperature and the spectra were recorded at wavelengths from 185 nm to 300 nm with a bandwidth of 1 nm, data pitch of 0.2 nm and scanning speed at 5 nm/min. The samples were measured three times and the collected spectra were averaged. Background measurements were performed using PBS buffer (50 mM, pH 7.4, 5 % TFE) and subtracted from the peptide spectra. The collected data was processed using *Spectra Analysis* by *JASCO* and *Origin Pro* by *OriginLab*®. TEM analysis of Fmoc-RQIKIWFQNR **3** (100 µM) in PBS (50 mM, pH 7.4, 5 % TFE) were performed according to the general procedure.

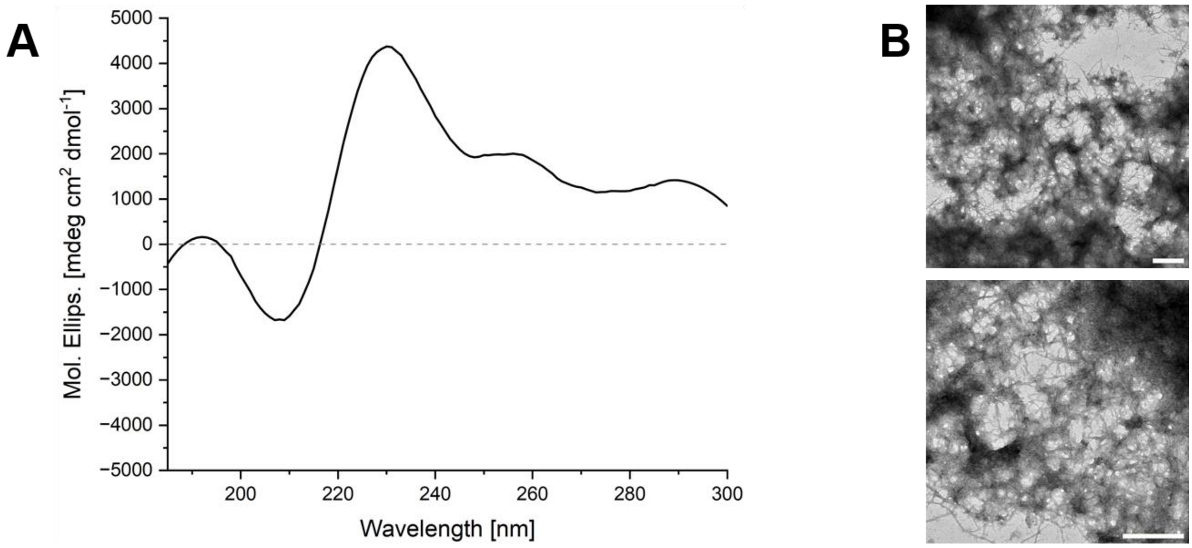

Figure S27: A CD-spectrum of Fmoc-RQIKIWFQNR **3** (100 µM) in PBS (50 mM, pH 7.4, 5 % TFE). The molar ellipticity is plotted against the wavelength. **B** TEM micrographs of Fmoc-RQIKIWFQNR **3** (100 µM) in PBS (50 mM, pH 7.4, 5 % TFE). The samples were stained with uranyl acetate (4 %), scale bar = 500 nm.

3.1.5 Transmission Electron Microscopy (TEM)

Peptides were dissolved in DMSO (10 mM) and used to prepare samples in PBS (50 mM, pH 7.4, 5 % DMSO) with 100 µM peptide concentrations. The samples were incubated for 24 h at room temperature and TEM grids were prepared by pipetting peptide solution (3 µL) onto a Formvar-coated copper grid and incubated for 5 min. After the incubation, the solutions were removed with filter paper and the grids were stained with uranyl acetate solution (4 %, 7 µL) for 2.5 min. The grids were washed three times with MilliQ-H_2O and dried before being measured by TEM.

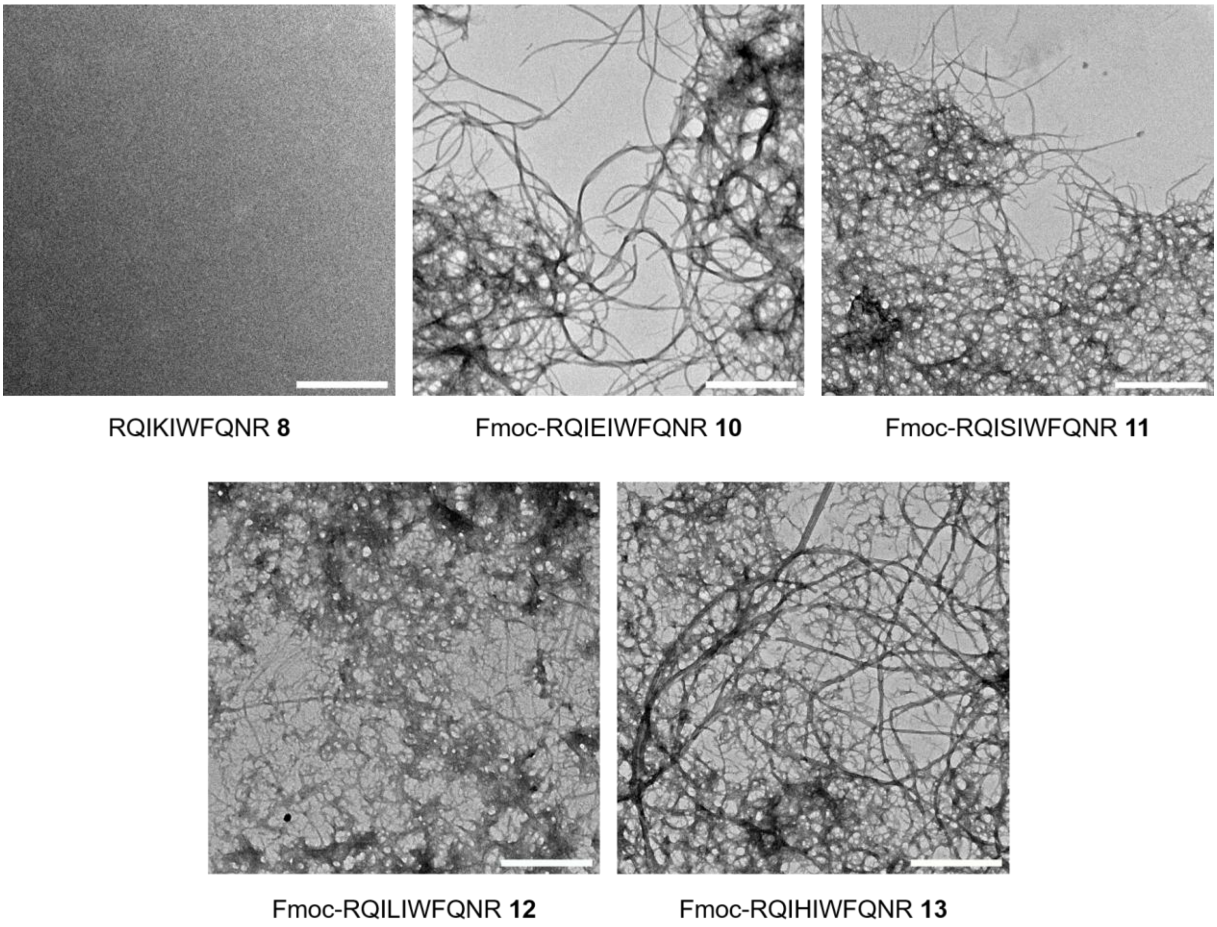

Figure S28: TEM micrographs of RQIKIWFQNR **8**, Fmoc-RQIEIWFQNR **10**, Fmoc-RQISIWFQNR **11**, Fmoc-RQILIWFQNR **12** and Fmoc-RQIHIWFQNR **13** (all 100 µM) in PBS (50 mM, pH 7.4, 5 % DMSO). TEM-grids were stained with uranyl acetate (4 %), scale bar = 500 nm.

3.1.6 Cryogenic Electron Microscopy (cryo-EM)

Fmoc-RQIKIWFQNR **3** (100 µM) and **3**-*co*-**4** (1:1, 200 µM) were pre-assembled for 24 h in PBS (50 mM, pH 7.4, 5 % DMSO) at room temperature. The sample (3 µL) was placed onto a Quantifoil 1.2/1.3 Cu 400 mesh grid, which was previously glow discharged. The grid was then blotted and plunged into liquid ethane with an automated plunging system (*Vitrobot* from *Thermo-Fisher Scientific*) and transferred in liquid nitrogen to the TEM (*Titan Krios G4*, *Thermo-Fisher Scientific*).

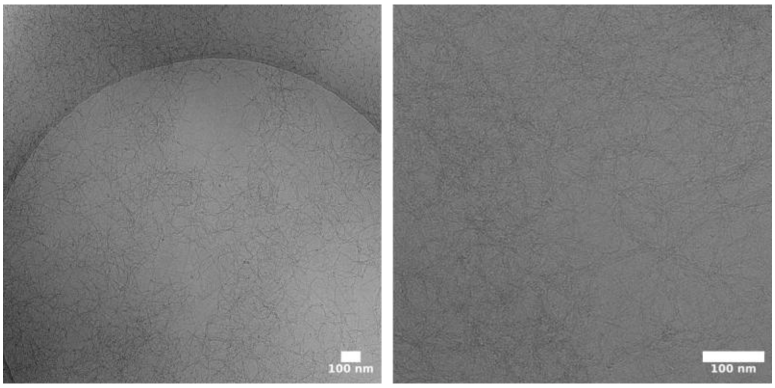

Figure S29: Cryo-EM pictures of Fmoc-RQIKIWFQNR **3** (100 µM) in PBS buffer (50 mM, pH 7.4, 5 % DMSO), scale bar = 100 nm.

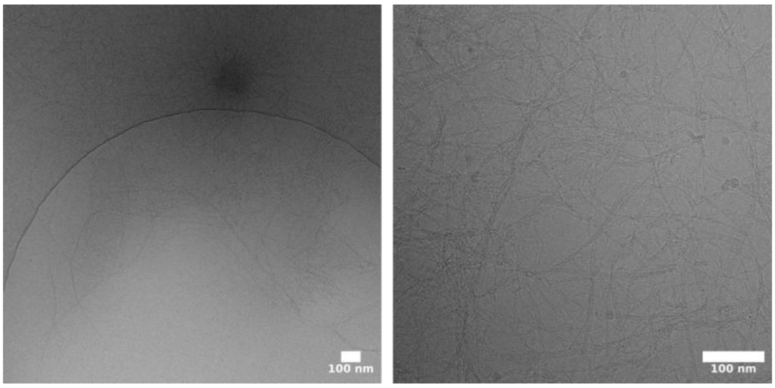

Figure S30: Cryo-EM pictures of co-assembly **3**-*co*-**4** (1:1, 100 µM each component) in PBS buffer (50 mM, pH 7.4, 5 % DMSO), scale bar = 100 nm.

3.1.7 Nile Red Assay

The homo-assemblies Fmoc-RQIKIWFQNR **3** and Fmoc-RQIRIWFQNR **4** were dissolved in the monomeric state in DMSO (1 mM) and diluted with PBS buffer (50 mM, pH 7.4) to obtain final peptide concentrations of 10 µM (5 % DMSO). Stock solutions of the peptides at 2 mM concentrations were used to prepare **3**-*co*-**4** in the monomeric state (1:1, 2 mM). The stock solution was sonicated for 5 min at room temperature and diluted with PBS buffer (50 mM, pH 7.4) to obtain peptide samples with 20 µM concentrations (5 % DMSO). Nile Red was added from a 1.2 mM DMSO stock to obtain final concentrations of 30 µM and the samples were analyzed regarding their fluorescence in a 96-well plate black (*Greiner*). The fluorescence intensity was measured at λ_{ex} = 550 nm and λ_{em} = 620 – 700 nm. The emission maximum was determined and plotted.

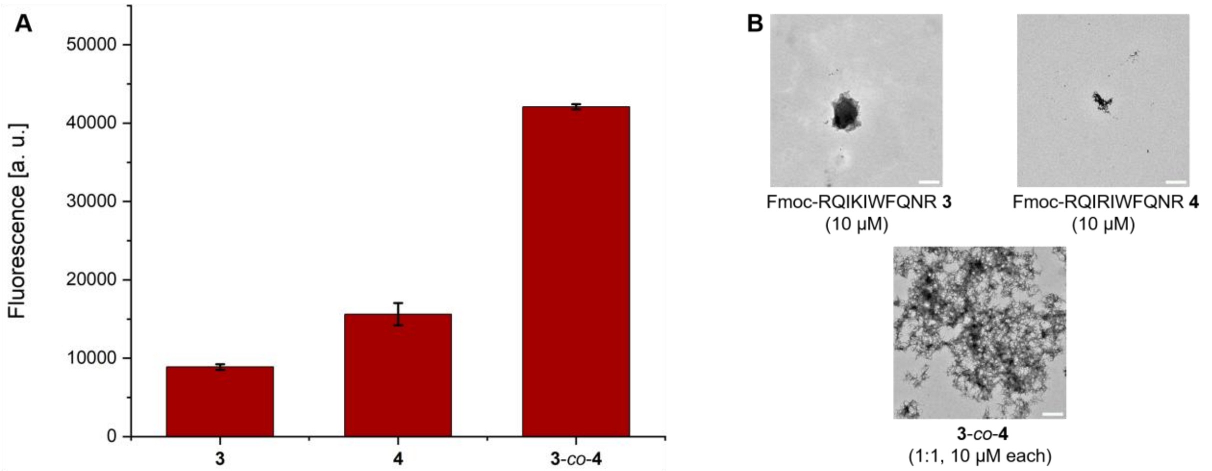

Figure S31: A Nile Red Assay fluorescence bar chart of Fmoc-RQIKIWFQNR **3**, Fmoc-RQIRIWFQNR **4** (both 10 µM) and the co-assembly **3**-*co*-**4** (1:1, 10 µM each component) in PBS (50 mM, pH 7.4, 5 % DMSO). The samples were incubated at room temperature for 24 h prior to analysis. The fluorescence intensity at λ_{ex} = 550 nm and λ_{em} = 660 nm is shown. **B** TEM micrographs of nanostructures formed by Fmoc-RQIKIWFQNR **3**, Fmoc-RQIRIWFQNR **4** (both 10 µM) and **3**-*co*-**4** (1:1, 10 µM each component) in PBS (50 mM, pH 7.4, 5 % DMSO), stained with uranyl acetate (4 %), scale bar = 500 nm.

3.1.8 Förster Resonance Energy Transfer (FRET)

The experiments were performed according to a procedure reported by Rost *et al.*[30] The analyzed peptides were dissolved in DMSO (10 mM) and used to prepare samples in PBS buffer (50 mM, pH 7.4, 5 % DMSO) with 50 µM peptide concentrations for the homo-assemblies and 100 µM for the co-assembly (1:1) and incubated for 24 h at room temperature. 100 µL of the peptide solutions were analyzed regarding their fluorescence in a *Greiner* 96 flat black well plate. The fluorescence intensity was measured at λ_{ex} = 464 nm and λ_{em} = 500 – 700 nm. The TAMRA-RQIKIWFQNR **14** spectrum was subtracted from the **9**-*co*-**14** spectrum.

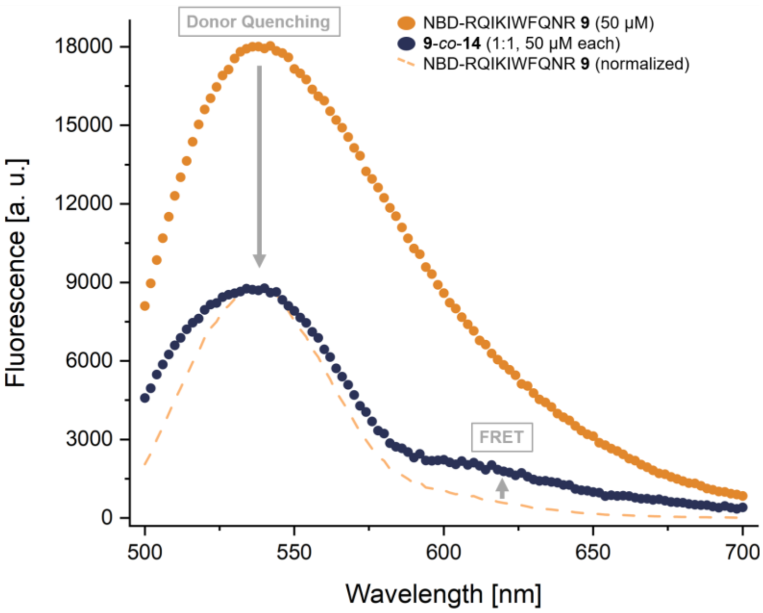

Figure S32: Fluorescence intensity of NBD-RQIKIWFQNR **9** (50 µM), **9**-*co*-**14** (1:1, 50 µM each component) and normalized NBD-RQIKIWFQNR **9** (50 µM) as a reference in PBS (50 mM, pH 7.4, 5 % DMSO) at λ_{ex} = 464 nm for λ_{em} = 500 – 700 nm. The **9**-*co*-**14** spectrum shows the results after subtraction from TAMRA-RQIKIWFQNR **14** fluorescence. The fluorescence intensity is plotted against the wavelength.

3.2 Catalytic Activity

3.2.1 Product Calibration and Fluorescence Intensity

Methodol **1** (5 mM) and 6-methoxy-2-naphthaldehyde **2** (500 µM) were dissolved in DMSO. The samples were diluted in PBS (50 mM, pH 7.4) to obtain final concentrations of **1** (50 µM) and **2** (5 µM) with 5 % DMSO. The samples were incubated for 24 h at room temperature in the dark. After incubation, the samples were analyzed in triplicates regarding their fluorescence emission intensity in a *Greiner* 384 flat back well plate at λ_{ex} = 330 nm and λ_{em} = 400 – 500 nm on a *TECAN* Spark 20M microplate reader (Figure S33 A).

6-Methoxy-2-naphthaldehyde **2** was dissolved in DMSO (10 mM). The stock was diluted with PBS (50 mM, pH 7.4) to obtain final concentrations of **2** (1 – 60 µM) with 5 % DMSO. The samples were analyzed in triplicates regarding their fluorescence emission intensity in a *Greiner* 96 flat back well plate at λ_{ex} = 330 nm and λ_{em} = 452 nm on a *TECAN* Spark 20M microplate reader (Figure S33 B).

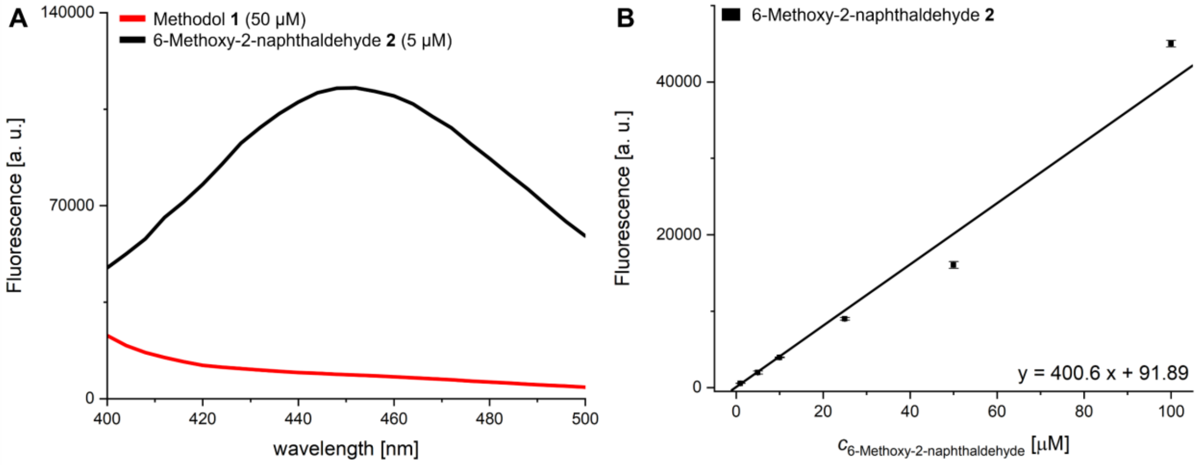

Figure S33: A Fluorescence intensity of Methodol **1** (50 µM) and 6-Methoxy-2-naphthaldehyde **2** (5 µM) at λ_{ex} = 330 nm in PBS (50 mM, pH 7.4, 5 % DMSO) for λ_{em} = 400 – 500 nm. The fluorescence intensity is plotted against the wavelength. **B** Fluorescence intensity of 6-methoxy-2-naphthaldehyde **2** at in PBS (50 mM, pH 7.4, 5 % DMSO). The fluorescence intensity is plotted against concentration of compound **2**. For the measured signals, a linear fit was calculated and plotted using *Origin Pro* by *OriginLab*®.

3.2.2 Catalytic Activity – Peptide Concentrations

Fmoc-RQIKIWFQNR **3**, Fmoc-RQIRIWFQNR **4** and **3**-*co*-**4** (1:1) were dissolved in DMSO at high concentrations (50 mM) and diluted in PBS (50 mM, pH 7.4) to obtain the peptides at concentrations of 5 – 500 µM and 5 % DMSO content. The samples were incubated for 24 h at room temperature. Methodol **1** (50 µM) and 6-methoxy-2-naphthaldehyde **2** (5 µM) were added and the samples were incubated for 24 h at 37 °C in the dark. After incubation, the samples were analyzed in triplicates regarding their fluorescence emission intensity in a *Greiner* 96 flat back well plate at λ_{ex} = 330 nm and λ_{em} = 452 nm on a *TECAN* Spark 20M microplate reader.

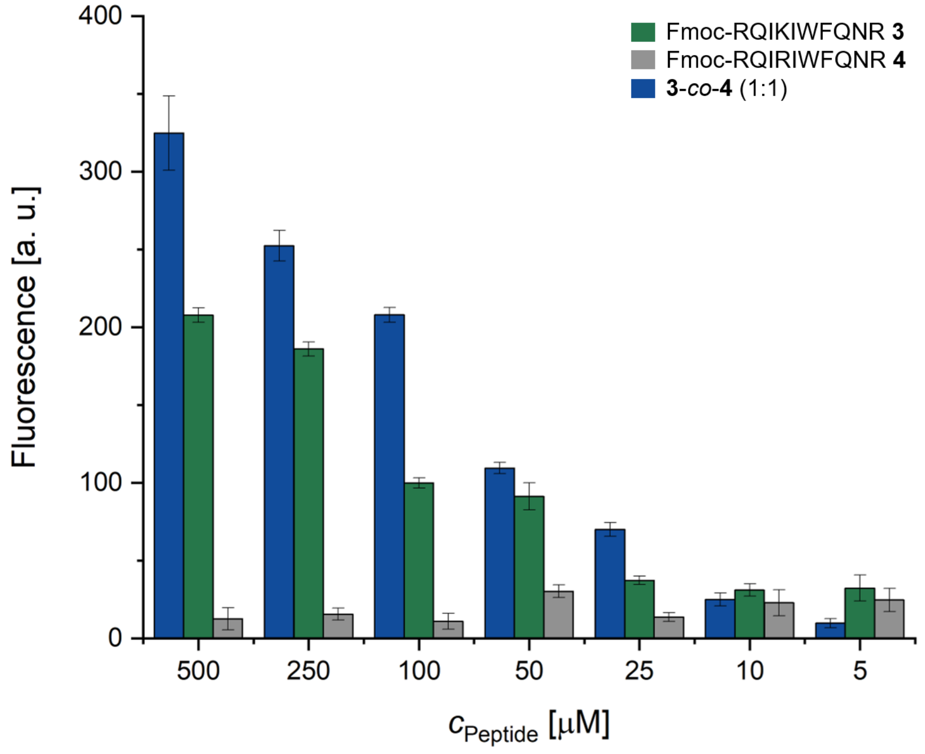

Figure S34: Fluorescence intensity bar chart of the Fmoc-RQIKIWFQNR **3**, Fmoc-RQIRIWFQNR **4** and co-assembly **3**-*co*-**4** (1:1) at 5 – 500 µM peptide concentrations in PBS (50 mM, pH 7.4, 5 % DMSO) at λ_{ex} = 330 nm and λ_{em} = 452 nm.

3.2.3 Stability Test Fmoc-RQIKIWFQNR

Fmoc-RQIKIWFQNR **3** was dissolved in high concentration in DMSO (10 mM). The DMSO stock was used to prepare two Fmoc-RQIKIWFQNR **3** samples (100 µM) in PBS (50 mM, pH 7.4, 5 % DMSO). One sample was incubated at room temperature for 24 h, whereas the second sample was incubated at room temperature for seven days. Methodol **1** (50 µM) and 6-methoxy-2-naphthaldehyde **2** (5 µM) were added to peptide samples and the reaction mixtures were incubated for 24 h at 37 °C. The samples were analyzed in triplicates regarding their fluorescence emission intensity in a *Greiner* 96 flat back well plate at λ_{ex} = 330 nm and λ_{em} = 452 nm on a *TECAN* Spark 20M microplate reader.

For TEM, Fmoc-RQIKIWFQNR **3** (100 µM) was pre-assembled in PBS (50 mM, pH 7.4, 5 % DMSO) at room temperature for seven days. TEM grids were prepared by pipetting peptide solution (3 µL) onto a Formvar-coated copper grid and incubated for 5 min. After the incubation, the solutions were removed with filter paper and the grids were stained with uranyl acetate solution (4 %, 7 µL) for 2.5 min. The grids were washed three times with MilliQ-H_2O and dried before being measured.

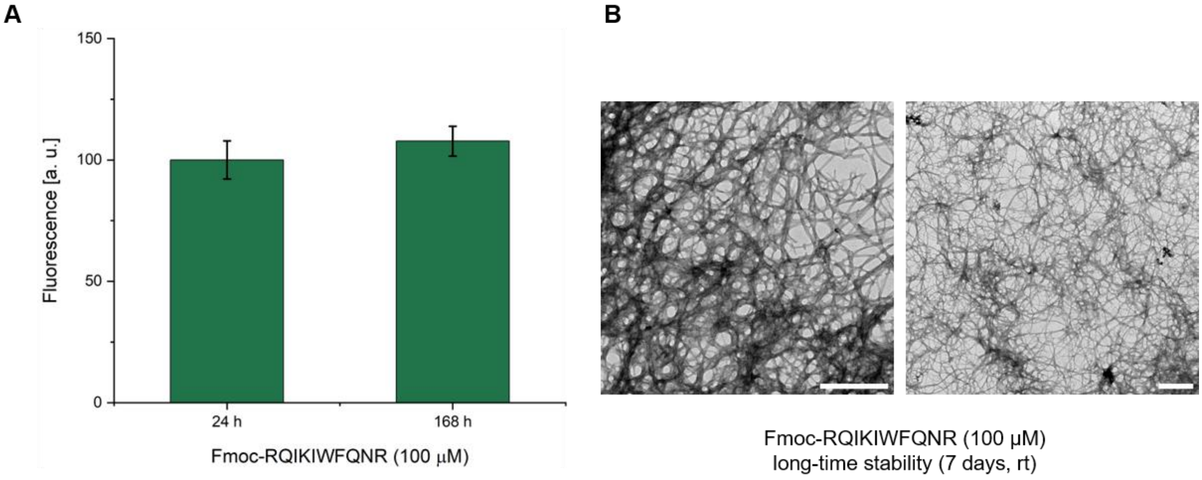

Figure S35: A Fluorescence intensity bar chart of Fmoc-RQIKIWFQNR **3** (100 µM) at λ_{ex} = 330 nm and λ_{em} = 452 nm. One peptide sample (24 h) was pre-assembled for 24 h at room temperature in PBS (50 mM, pH 7.4, 5 % DMSO). The second sample (168 h) was incubated in PBS (50 mM, pH 7.4, 5 % DMSO) for seven days at room temperature. **B** TEM micrographs of Fmoc-RQIKIWFQNR **3** (100 µM) in PBS (50 mM, pH 7.4, 5 % DMSO) after incubation for seven days at room temperature. TEM-grids were stained with uranyl acetate (4 %), scale bar = 500 nm.

Fmoc-RQIKIWFQNR **3** was dissolved in high concentration in DMSO (10 mM). The DMSO stock was used to prepare two Fmoc-RQIKIWFQNR **3** samples (100 µM) in PBS (50 mM, pH 7.4, 5 % DMSO) and incubated at room temperature for 24 h. One sample was ultra-sonicated at room temperature for 5 min. Methodol **1** (50 µM) and 6-methoxy-2-naphthaldehyde **2** (5 µM) were added to peptide samples and the reaction mixtures were incubated for 2 h at 37 °C. The samples were analyzed in triplicates regarding their fluorescence emission intensity in a *Greiner* 96 flat back well plate at λ_{ex} = 330 nm and λ_{em} = 452 nm on a *TECAN* Spark 20M microplate reader.

For TEM, Fmoc-RQIKIWFQNR **3** (100 µM) was pre-assembled in PBS (50 mM, pH 7.4, 5 % DMSO) at room temperature for 24 h. The peptide solution was ultra-sonicated for 5 min at room temperature and incubated for 2 h at 37 °C. TEM grids were prepared by pipetting peptide solution (3 µL) onto a Formvar-coated copper grid and incubated for 5 min. After incubation, the solutions were removed with filter paper and the grids were stained with uranyl acetate solution (4 %, 7 µL) for 2.5 min. The grids were washed three times with MilliQ-H_2O and dried prior to measurement.

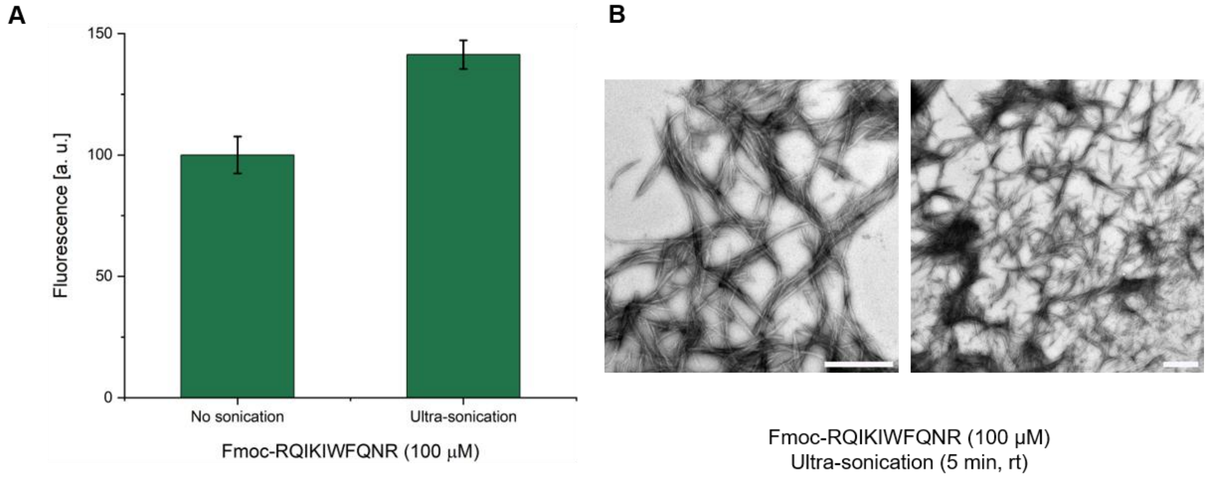

Figure S36: A Fluorescence intensity bar chart of Fmoc-RQIKIWFQNR **3** (100 µM) at λ_{ex} = 330 nm and λ_{em} = 452 nm. The peptides were pre-assembled for 24 h at room temperature in PBS (50 mM, pH 7.4, 5 % DMSO). The ultra-sonication sample was ultra-sonicated for 5 min at room temperature after incubation. **B** TEM micrographs of ultra-sonicated Fmoc-RQIKIWFQNR **3** (100 µM) in PBS (50 mM, pH 7.4, 5 % DMSO). TEM-grids were stained with uranyl acetate (4 %), scale bar = 500 nm.

3.2.4 Michaelis-Menten Kinetics

The analyzed peptides were dissolved in high concentrations in DMSO (10 mM) and used to prepare Fmoc-RQIKIWFQNR **3** (100 µM) and Fmoc-RQFKFWFQNR **6** (100 µM) samples in PBS (50 mM, pH 7.4, 5 % DMSO). The samples were incubated for 2 h at room temperature. Methodol **1** at different concentrations (500 / 400 / 250 / 200 / 150 / 100 / 50 / 25 µM) and 6-methoxy-2-naphthaldehyde **2** (5 µM) were added to the peptide and analyzed regarding their fluorescence intensity in triplicates at λ_{ex} = 330 nm and λ_{em} = 452 nm after every 30 min over 24 h. The catalytic rate *v* was determined through linear fitting of the first ten measurement points.

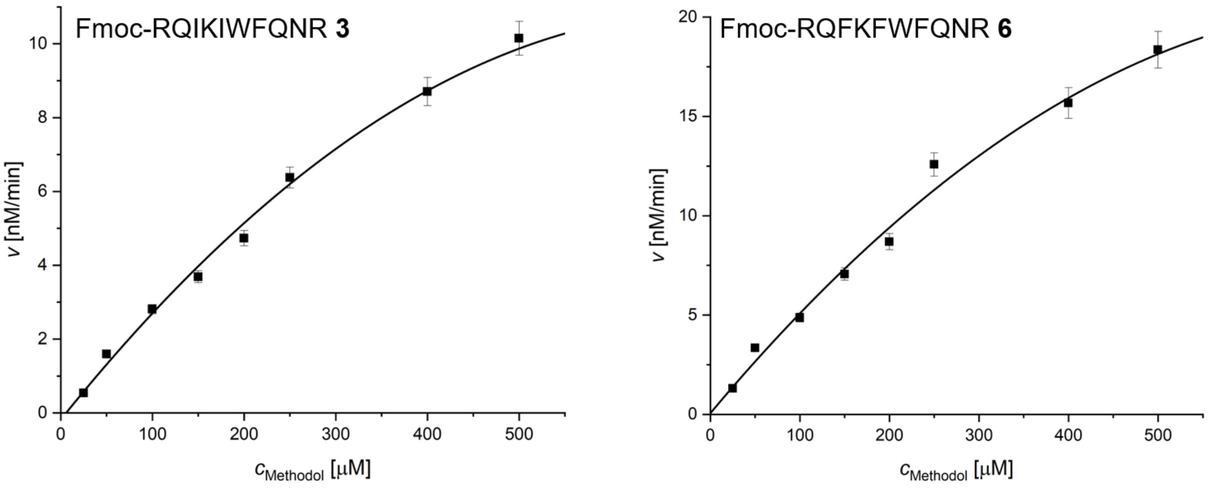

Figure S37: Scatter diagram depicting the reaction rate *v* of Fmoc-RQIKIWFQNR **3** (100 µM) and Fmoc-RQFKFWFQNR **6** (100 µM) in PBS (50 mM, pH 7.4, 5 % DMSO) at different Methodol **1** concentrations including a polynomial fit of first order calculated using *Origin Pro* by *OriginLab*®.

The Michaelis-parameters were calculated and summarized in Table S2.

Table S2: Summary of the Michaelis-Menten constants K_M and catalytic rates v_{max} at the highest Methodol **1** concentrations of Fmoc-RQIKIWFQNR **3** and Fmoc-RQFKFWFQNR **6** (both 100 µM) in PBS (50 mM, pH 7.4, 5 % DMSO).

Peptide	K_M [µM]	v_{max} [nM/min]
Fmoc-RQIKIWFQNR **3**	253.5 ± 10.2	10.2 ± 0.5
Fmoc-RQFKFWFQNR **6**	208.0 ± 8.7	18.4 ± 0.9

For the co-assemblies, the analyzed peptides were dissolved in DMSO (20 mM) and used to prepare co-assembly samples **3**-*co*-**4**, **3**-*co*-**10**, **3**-*co*-**11**, **3**-*co*-**12** and **3**-*co*-**13** (1:1, 200 µM) in PBS (50 mM, pH 7.4, 5 % DMSO). The samples were incubated for 2 h at room temperature.

Methodol **1** at different concentrations (500 / 400 / 250 / 200 / 150 / 100 / 50 / 25 µM) and 6-methoxy-2-naphthaldehyde **2** (5 µM) were added to the peptide and analyzed regarding their fluorescence intensity in triplicates at λ_{ex} = 330 nm and λ_{em} = 452 nm after every 30 min over 24 h. The catalytic rate *v* was determined through linear fitting of the first ten measurement points.

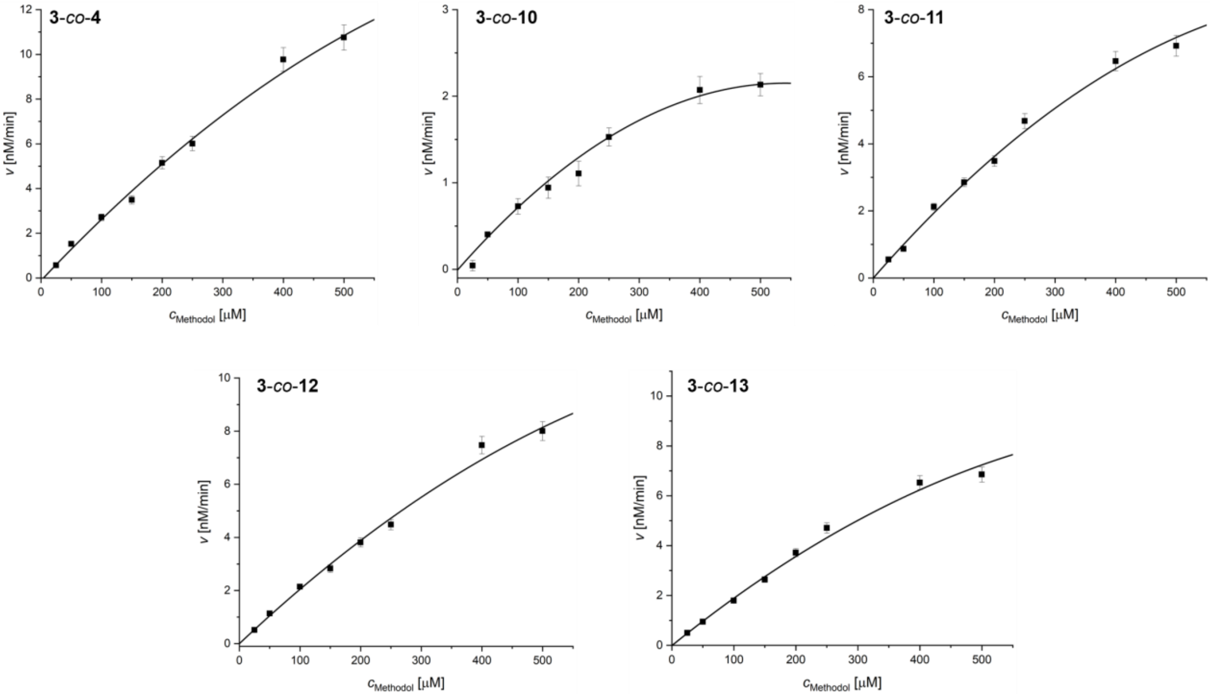

Figure S38: Scatter diagram depicting the reaction rate *v* of **3**-*co*-**4**, **3**-*co*-**10**, **3**-*co*-**11**, **3**-*co*-**12** and **3**-*co*-**13** (all 1:1, 100 µM each component) in PBS (50 mM, pH 7.4, 5 % DMSO) at different Methodol **1** concentrations including a polynomial fit of first order calculated using *Origin Pro* by *OriginLab*®.

The Michaelis-parameters were calculated and summarized in Table S3.

Table S3: Summary of the Michaelis-Menten constants K_M and catalytic rates v_{max} at the highest Methodol **1** concentrations of **3**-*co*-**4**, **3**-*co*-**10**, **3**-*co*-**11**, **3**-*co*-**12** and **3**-*co*-**13** (all 1:1, 200 µM) in PBS (50 mM, pH 7.4, 5 % DMSO).

**Peptide**	**K_M [µM]**	**v_{max} [nM/min]**
**3**-*co*-**4**	223.7 ± 10.2	10.8 ± 0.6
**3**-*co*-**10**	198.9 ± 8.9	2.1 ± 0.1
**3**-*co*-**11**	206.6 ± 7.7	6.9 ± 0.3
**3**-*co*-**12**	216.9 ± 8.0	8.0 ± 0.4
**3**-*co*-**13**	207.2 ± 7.7	6.9 ± 0.3

3.2.5 Co-Assembly of Active Peptides

The analyzed peptides Fmoc-RQIKIWFQNR **3** and Fmoc-RQFKFWFQNR **6** were dissolved in high concentrations in DMSO (10 mM) and used to prepare a co-assembly stock **3**-*co*-**6** (1:1, 10 mM), which was sonicated for 5 min at room temperature. Samples of Fmoc-RQIKIWFQNR **3** (100 µM), Fmoc-RQFKFWFQNR **6** (100 µM), **3**-*co*-**6** (1:1, 100 µM) and separately assembled **3** + **6** (1:1, 100 µM) were prepared in PBS (50 mM, pH 7.4, 5 % DMSO). The samples were incubated for 24 h at room temperature. Methodol **1** (50 µM) and 6-methoxy-2-naphthaldehyde **2** (5 µM) were added to the peptides. The samples were incubated for 24 h at 37 °C in the dark and analyzed regarding their fluorescence intensity in triplicates at λ_{ex} = 330 nm and λ_{em} = 452 nm in a 96-well plate black (*Greiner*). The measurements were performed on a *TECAN* Spark 20M.

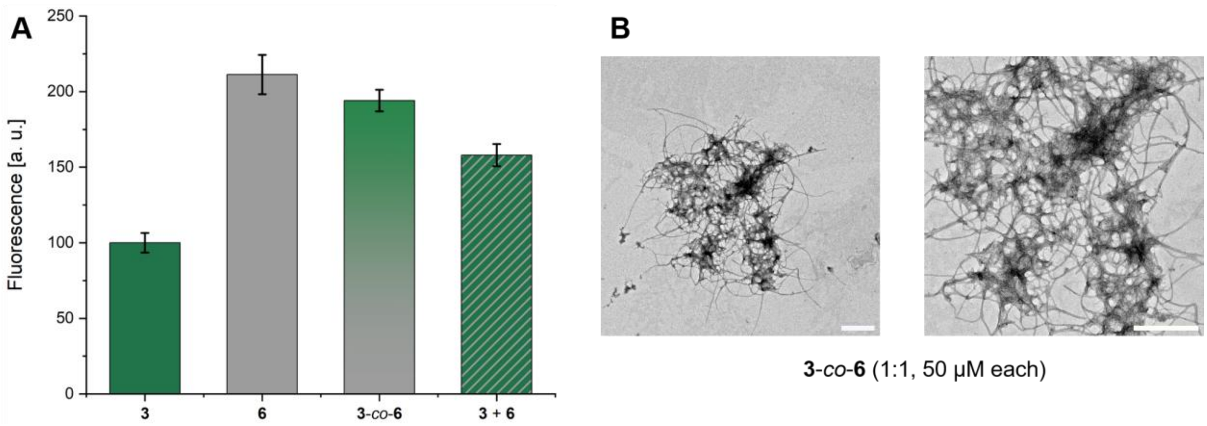

Figure S39: Fluorescence intensity plot of Fmoc-RQIKIWFQNR **3** (100 µM), Fmoc-RQFKFWFQNR **6** (100 µM), **3**-*co*-**6** (1:1, 50 µM each component) and **3** + **6** (1:1, 50 µM each component) in PBS (50 mM, pH 7.4, 5 % DMSO) with Methodol **1** (50 µM) and 6-methoxy-2-naphthaldehyde **2** (5 µM) at λ_{ex} = 330 nm and λ_{em} = 452 nm. The samples were incubated for 24 h at room temperature prior to addition of Methodol **1** and 6-methoxy-2-naphthaldehyde **2**.

3.2.6 Catalytic Activity – inactive homo-cSAP's

Fmoc-RQIKIWFQNR **3**, Fmoc-RQIRIWFQNR **4**, Fmoc-RQIEIWFQNR **10**, Fmoc-RQISIWFQNR **11**, Fmoc-RQILIWFQNR **12** and Fmoc-RQIHIWFQNR **13** were dissolved in DMSO at high concentrations (10 mM) and diluted in PBS (50 mM, pH 7.4) to obtain the peptides at 100 µM and 5 % DMSO content. The samples were incubated for 24 h at room temperature. Methodol **1** (50 µM) and 6-methoxy-2-naphthaldehyde **2** (5 µM) were added and the samples were incubated for 24 h at 37 °C in the dark. After incubation, the samples were analyzed in triplicates regarding their fluorescence emission intensity in a *Greiner* 96 flat back well plate at λ_{ex} = 330 nm and λ_{em} = 452 nm on a *TECAN* Spark 20M microplate reader.

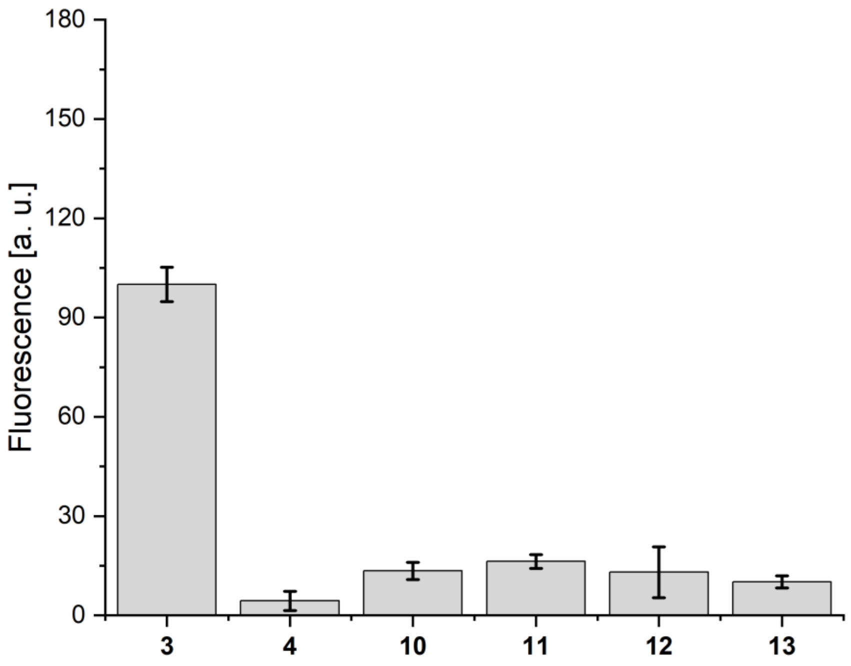

Figure S40: Fluorescence intensity bar chart of Fmoc-RQIKIWFQNR **3**, Fmoc-RQIRIWFQNR **4**, Fmoc-RQIEIWFQNR **10**, Fmoc-RQISIWFQNR **11**, Fmoc-RQILIWFQNR **12** and Fmoc-RQIHIWFQNR **13** (all 100 µM) at λ_{ex} = 330 nm and λ_{em} = 452 nm after 24 h incubation with Methodol **1**.

3.2.7 Modelling and MD Simulations

AF3 was used to predict arrangements of peptide fibrils consisting of 20 monomers (without Fmoc). For each peptide, the top five models were evaluated. AF3 predicted similar arrangements with minor differences, such as in the distribution of peptides as 10 x 2 double sheet or variations of that. The arrangement of peptide side chains towards the inner dry interface and the outer interface was similar in the models. For RQIKIWFQNR, the top model (rank 0 out of 0 – 4) was used. The predicted structure has all peptides aligned in parallel within each sheet and antiparallel between the two sheets. Hydrophilic groups, including Lys, are exposed to the outer interface while hydrophobic groups (Ile, Phe) are oriented towards the

inside. A similar arrangement has been observed for RQIRIWFQNR. The 2nd top model (rank 1) has been used because it represented a 10 x 2 fibril (model 0 had one sheet with 11 and one with 9 peptides). For RQFKFWFQNR model 0 was used. The replacement of Ile with Phe led to a more crowded dry interface, and the model presented antiparallel arrangements within each sheet. AF3 predicted for the four top ranked models of the RQIKIWFQNR and RQIRIWFQNR co-assembly (1:1), that all Lys and Arg residues are each positioned within one of the sheets, i.e. one sheet had all ten Lys residues and the other sheet all ten Arg residues. Only the fifth model showed some mixing. Antiparallel arrangements within each sheet were observed as well. Whether the proposed AF3 models are true requires further experimental verification. These models were used as starting models and are not yet considering the *N*-terminal Fmoc-modification which was manually added prior to MD simulation.

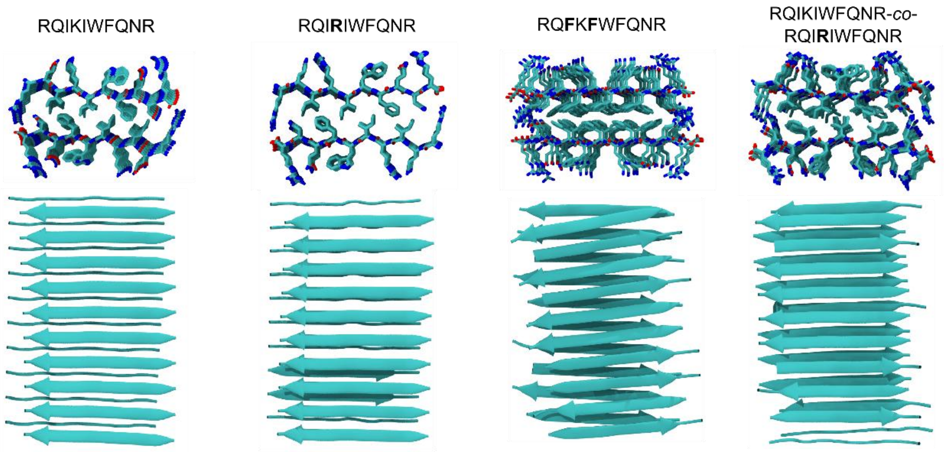

Figure S41: Structural models for peptide fibrils without Fmoc (20mers) using AF3. Top and side views of fibril models are shown. RQIKIWFQNR (global pLDDT 49.03, pTM 0.62), RQIRIWFQNR (global pLDDT 46.96, pTM 0.62), RQFKFWFQNR (global pLDDT 35.41, pTM 0.24), RQIKIWFQNR-*co*-RQIRIWFQNR (global pLDDT 40.83, pTM 0.36). pLDDT (predicted local distance difference test) and pTM (predicted template modelling) are confidence scores.

MD simulations of the AF3 models, extended by the Fmoc modification, were performed to obtain a representative structure of the peptide fibrils. For each peptide, three independent 100 ns long MD simulations were performed and the central structure of all frames was determined using cluster analysis. As already partially visible in the AF3 models, a higher twisting can be seen for the Fmoc-RQFKFWFQNR **6** fibril and the **3**-*co*-**4** assembly. The fully parallel alignment within each sheet in the models of Fmoc-RQIKIWFQNR **3** and Fmoc-RQIRIWFQNR **4** also means that the Lys and Arg residues are all aligned, while these residues are more distributed on each sheet surface for the partially antiparallel arranged fibrils of **6** and **3**-*co*-**4**.

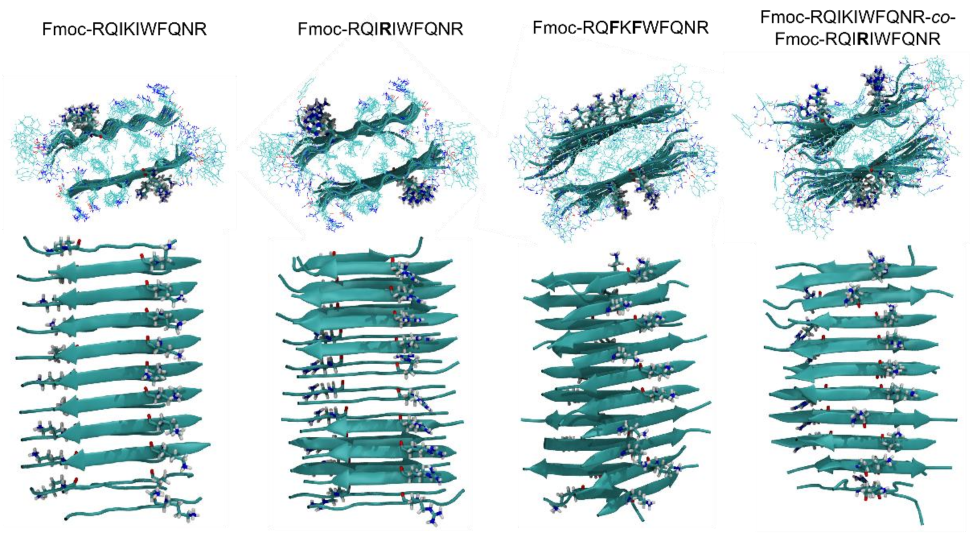

Figure S42: Representative fibril structures with Fmoc after MD simulation of Fmoc-RQIKIWFQNR **3**, Fmoc-RQIRIWFQNR **4**, Fmoc-RQFKFWFQNR **6** and **3**-*co*-**4** (1:1). The most dominant fibril structures after 100 ns of MD simulation in three replicates are shown. Clustering of the structures during the last 10 ns of each replicate was performed using the gromos method with RMSD cutoff of 0.35 – 0.44 nm in *GROMACS*. The peptides are visualized as cartoon with the Lys and Arg residues (position 4) highlighted with side chains. The top view also includes the full structures of all side chains and Fmoc to visualize the dry interface between the fibrils.

The fibril models were then used and five Methodol **1** molecules added. Each system was simulated for 300 ns in triplicates. The most dominant arrangements for each system were determined using clustering analysis. These structures do not represent permanent or stable configurations, being snapshots from the trajectories which have been either observed multiple times or the substrate **1** bound to the fibril for longer times in these more favored arrangements. For each dominant cluster structure, a top and two side views are provided.

Table S4: Overview of the size of the largest structural clusters of the simulation trajectories. The three largest clusters and any with at least 5 % abundance are listed and the central structures visualized.

Systems	Largest Clusters
Fmoc-RQIKIWFQNR **3**	Cluster 1 (9.3 %), Cluster 2 (8.7 %), Cluster 3 (3.4 %)
Fmoc-RQIRIWFQNR **4**	Cluster 1 (7.8 %), Cluster 2 (6.9 %), Cluster 3 (4.2 %)
Fmoc-RQFKFWFQNR **6**	Cluster 1 (8.4 %), Cluster 2 (4.2 %), Cluster 3 (3.3 %)
**3**-*co*-**4**	Cluster 1 (6.9 %), Cluster 2 (5.8 %), Cluster 3 (5.7 %), Cluster 4 (5.4 %)

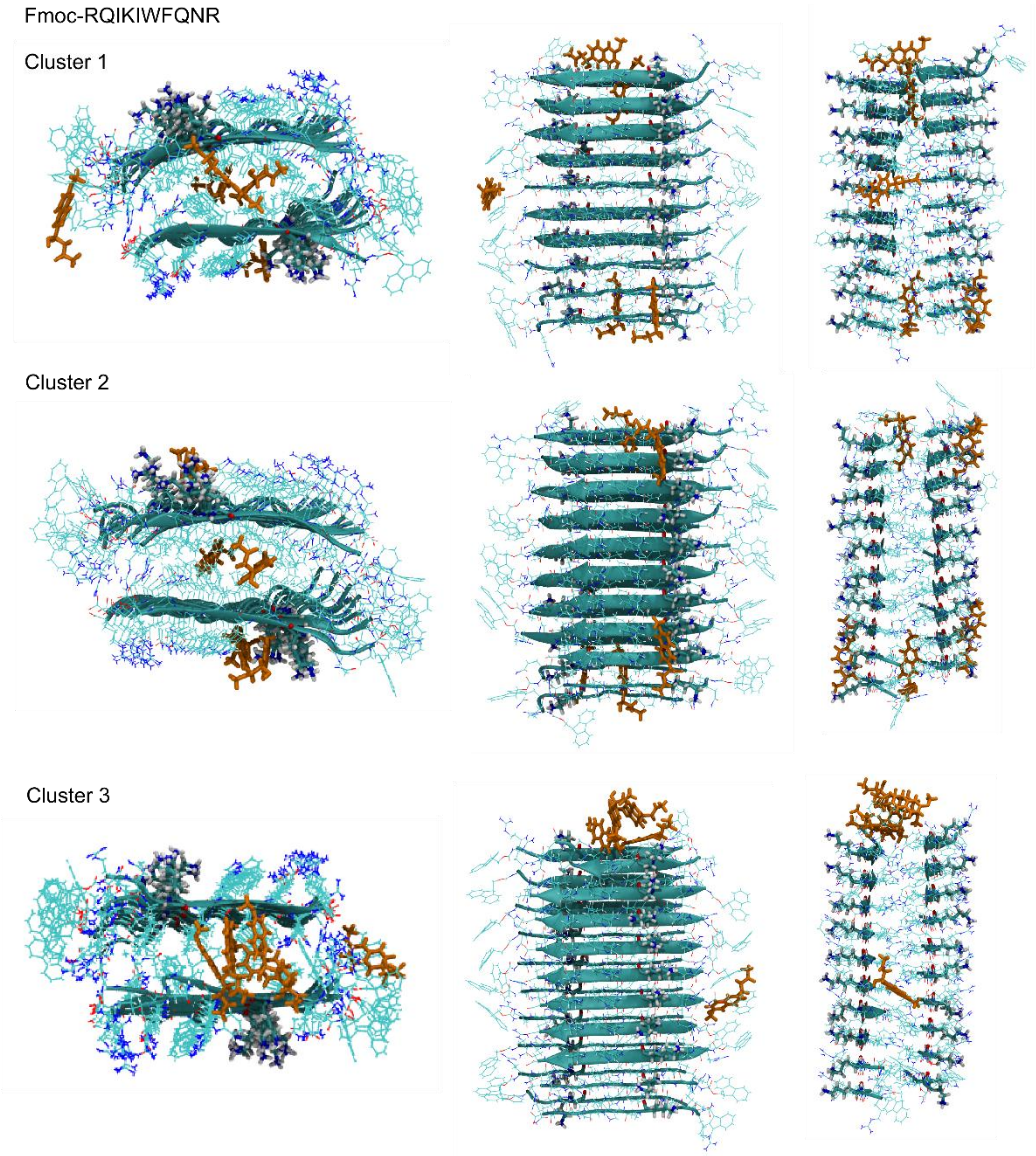

Figure S43: Dominant binding motifs of Methodol **1** to Fmoc-RQIKIWFQNR **3**. The three most dominant binding motifs of five Methodol **1** molecules (in orange) to the peptide fibril are shown. Clustering of structures of the three replicates (each 300 ns) was performed using the gromos method with RMSD cutoff of 0.5 nm in *GROMACS*. The fibrils are visualized as cartoons with the side chains as lines and the Lys residues (position 4) highlighted.

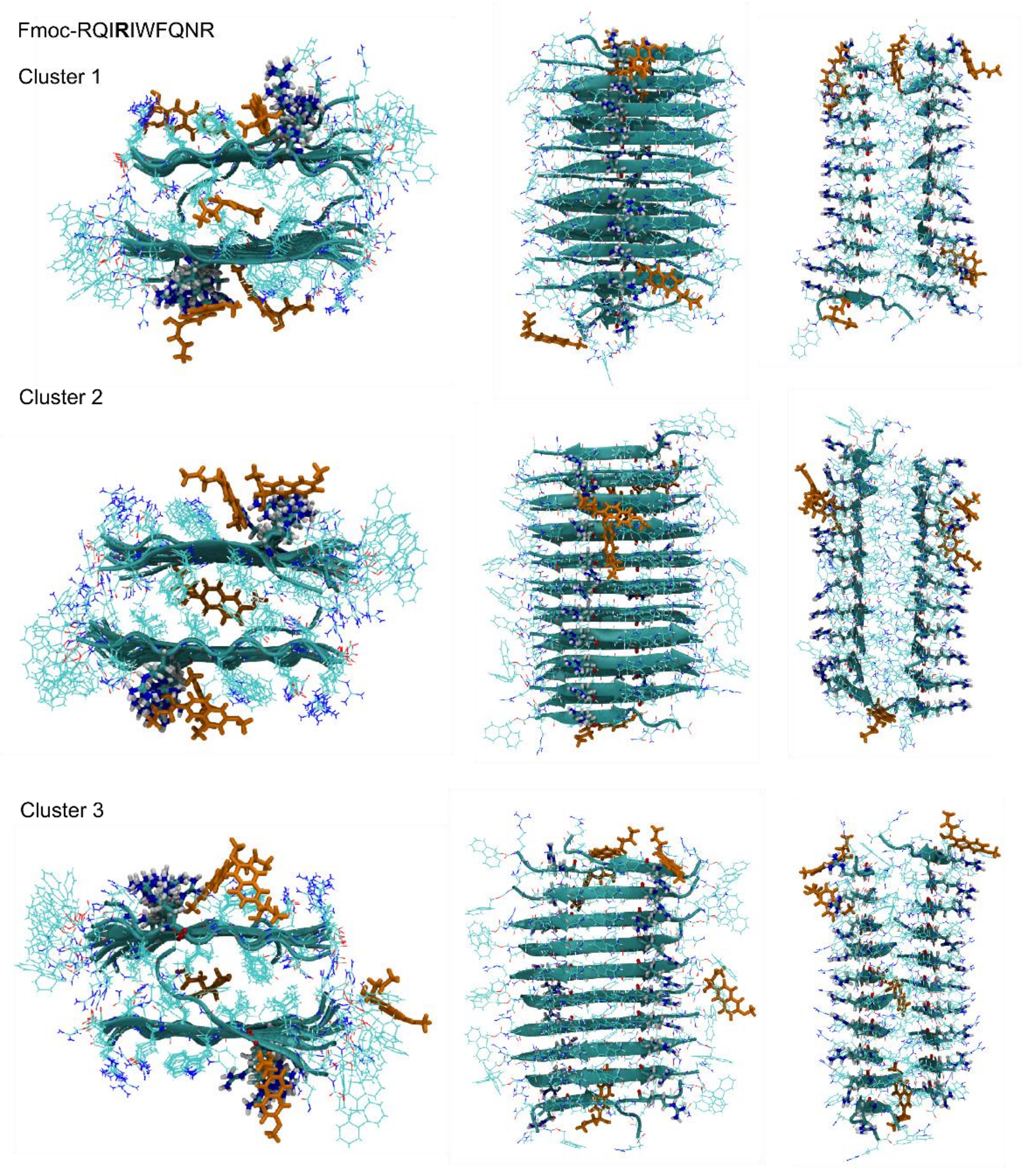

Figure S44: Dominant binding motifs of Methodol **1** to Fmoc-RQIRIWFQNR **4**. The three most dominant binding motifs of five Methodol **1** molecules (in orange) to the peptide fibril are shown. Clustering of structures of the three replicates (each 300 ns) was performed using the gromos method with RMSD cutoff of 0.5 nm in *GROMACS*. The fibrils are visualized as cartoons with the side chains as lines and the Arg residues (position 4) highlighted.

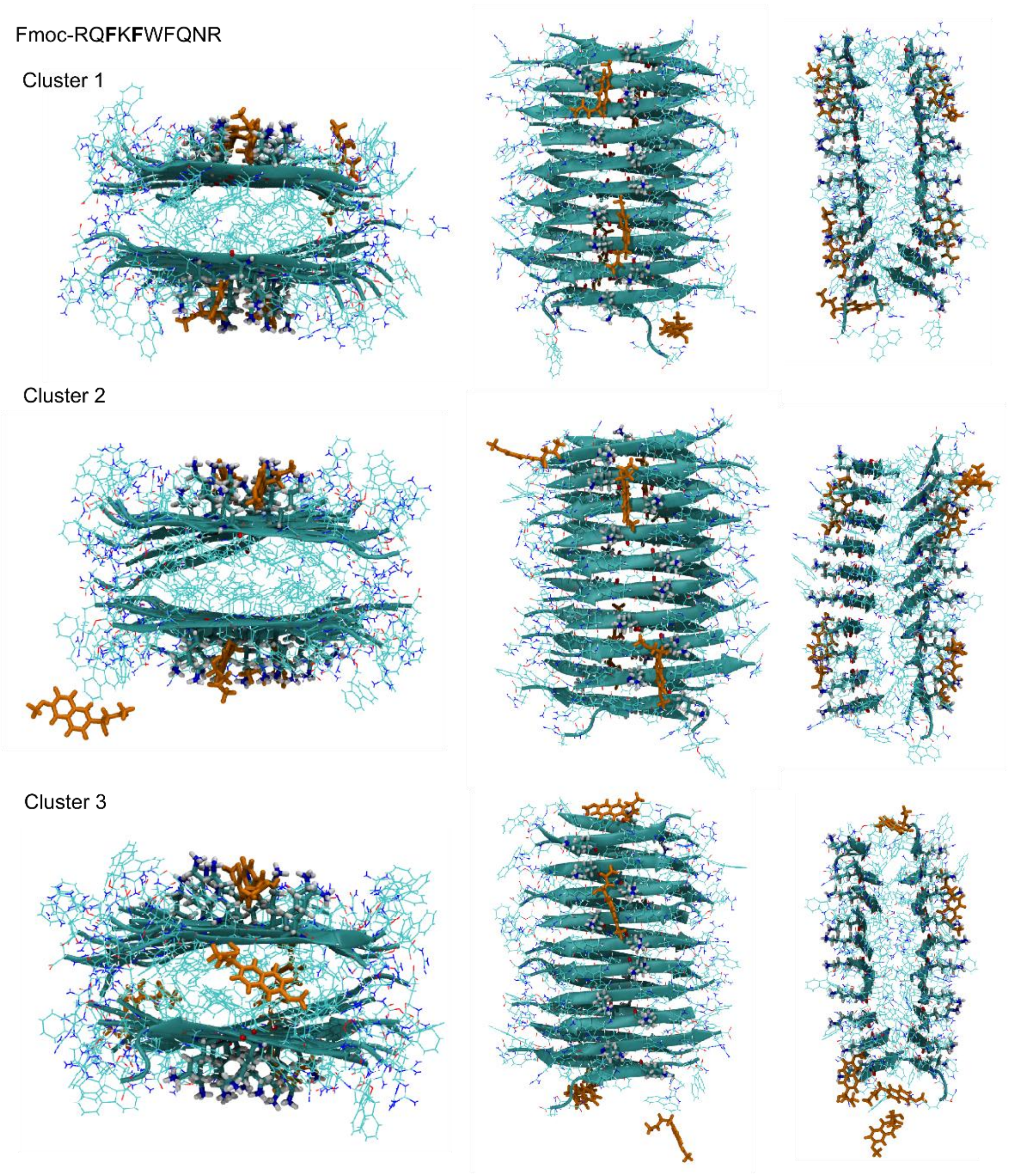

Figure S45: Dominant binding motifs of Methodol **1** to Fmoc-RQFKFWFQNR **6**. The three most dominant binding motifs of five Methodol **1** molecules (in orange) to the peptide fibril are shown. Clustering of structures of the three replicates (each 300 ns) was performed using the gromos method with RMSD cutoff of 0.5 nm in *GROMACS*. The fibrils are visualized as cartoons with the side chains as lines and the Lys residues (position 4) highlighted.

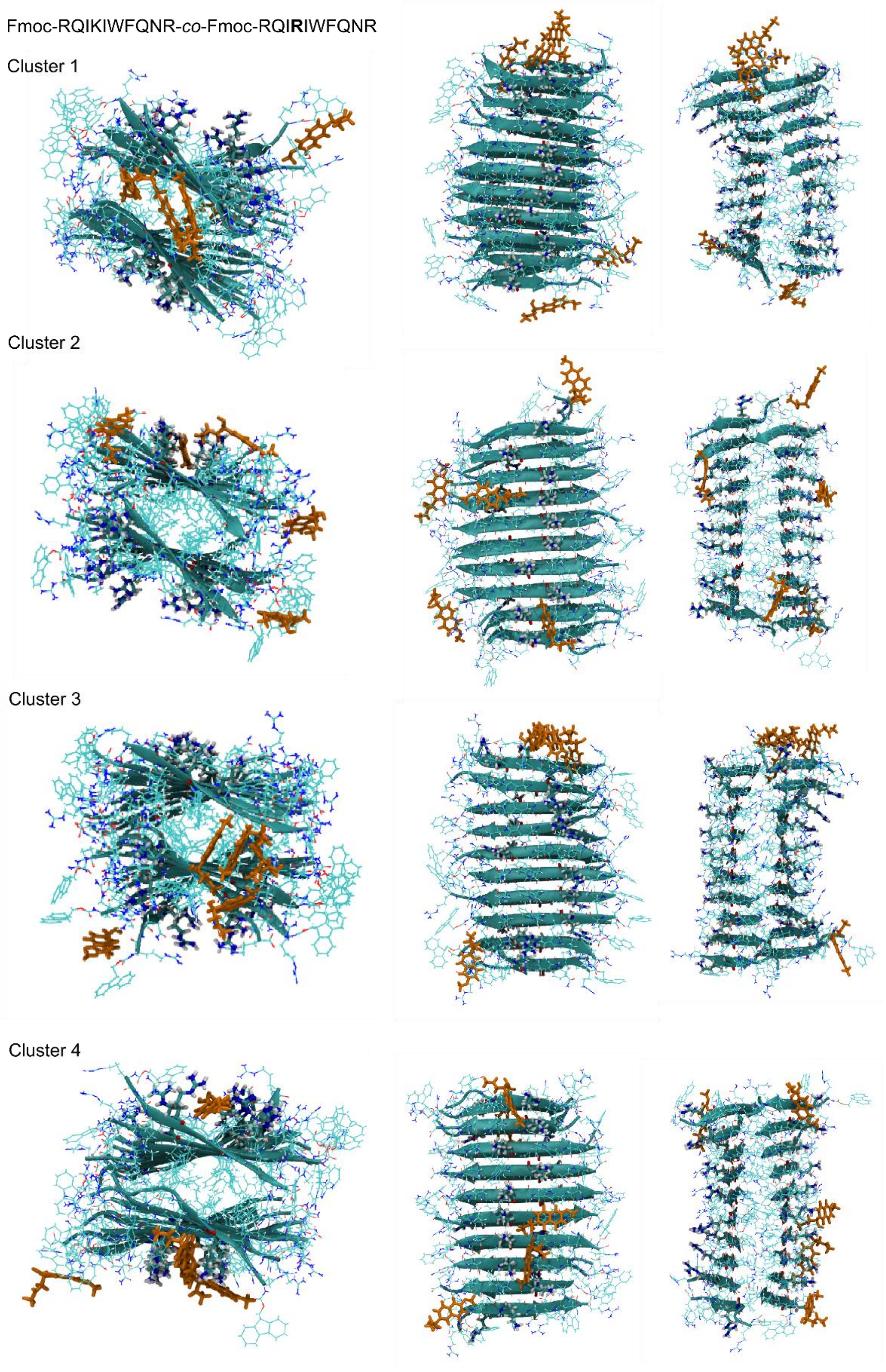

Figure S46: Dominant binding motifs of Methodol **1** to **3**-*co*-**4** (1:1). The three most dominant binding motifs of five Methodol **1** molecules (in orange) to the peptide fibril are shown. Clustering of structures of the three replicates (each 300 ns) was performed using the gromos method with RMSD cutoff of 0.5 nm in *GROMACS*. The fibrils are visualized as cartoons with the side chains as lines and the Lys and Arg residues (position 4) highlighted.

The minimum distance between the Methodol **1** molecules and any of the Lys or Arg (position 4) in the fibrils was determined over the 900 ns simulation time (300 ns in triplicate).

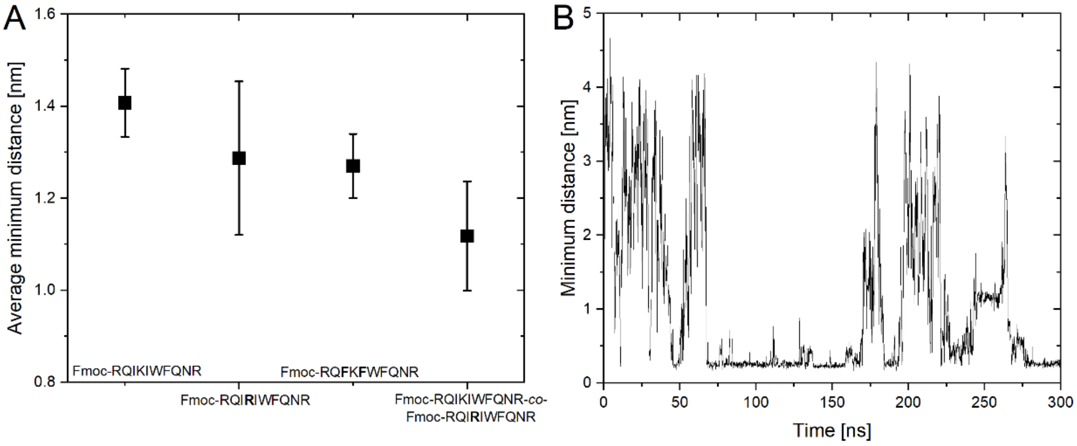

Figure S47: Minimum distances between Methodol **1** and Lys or Arg residues (position 4) in the fibrils. The minimum distance between each Methodol **1** molecule and any of the Lys or Arg residues (position 4) in the fibrils was determined and averaged for all five Methodol **1** molecules over the entire 300 ns for all replicates. **A** Averages and standard errors are shown. **B** The minimum distance of an individual Methodol **1** molecule with a fibril is shown over time for one replicate as an example.

References

[1] J. Abramson, J. Adler, J. Dunger, R. Evans, T. Green, A. Pritzel, O. Ronneberger, L. Willmore, A. J. Ballard, J. Bambrick, S. W. Bodenstein, D. A. Evans, C.-C. Hung, M. O'Neill, D. Reiman, K. Tunyasuvunakool, Z. Wu, A. Žemgulytė, E. Arvaniti, C. Beattie, O. Bertolli, A. Bridgland, A. Cherepanov, M. Congreve, A. I. Cowen-Rivers, A. Cowie, M. Figurnov, F. B. Fuchs, H. Gladman, R. Jain, Y. A. Khan, C. M. R. Low, K. Perlin, A. Potapenko, P. Savy, S. Singh, A. Stecula, A. Thillaisundaram, C. Tong, S. Yakneen, E. D. Zhong, M. Zielinski, A. Žídek, V. Bapst, P. Kohli, M. Jaderberg, D. Hassabis, J. M. Jumper, *Nature* **2024**, *630*, 493-500.

[2] W. Humphrey, A. Dalke, K. Schulten, *J. Mol. Graph.* **1996**, *14*, 33-38.

[3] J. Huang, A. D. MacKerell Jr, *J. Comput. Chem.* **2013**, *34*, 2135-2145.

[4] J. Huang, S. Rauscher, G. Nawrocki, T. Ran, M. Feig, B. L. de Groot, H. Grubmüller, A. D. MacKerell, *Nat. Methods* **2017**, *14*, 71-73.

[5] M. D. Hanwell, D. E. Curtis, D. C. Lonie, T. Vandermeersch, E. Zurek, G. R. Hutchison, *J. Cheminformatics* **2012**, *4*, 17.

[6] K. Vanommeslaeghe, E. Hatcher, C. Acharya, S. Kundu, S. Zhong, J. Shim, E. Darian, O. Guvench, P. Lopes, I. Vorobyov, A. D. Mackerell Jr., *J. Comput. Chem.* **2010**, *31*, 671-690.

[7] K. Vanommeslaeghe, A. D. MacKerell, Jr., *Journal of Chemical Information and Modeling* **2012**, *52*, 3144-3154.

[8] K. Vanommeslaeghe, E. P. Raman, A. D. MacKerell, Jr., *J. Chem. Inf. Model.* **2012**, *52*, 3155-3168.

[9] W. L. Jorgensen, J. Chandrasekhar, J. D. Madura, R. W. Impey, M. L. Klein, *J. Chem. Phys.* **1983**, *79*, 926-935.

[10] X. Daura, K. Gademann, B. Jaun, D. Seebach, W. F. van Gunsteren, A. E. Mark, *Angew. Chem. Int. Ed.* **1999**, *38*, 236-240.

[11] M. J. Abraham, T. Murtola, R. Schulz, S. Páll, J. C. Smith, B. Hess, E. Lindahl, *SoftwareX* **2015**, *1-2*, 19-25.

[12] S. Páll, M. J. Abraham, C. Kutzner, B. Hess, E. Lindahl, in *Solving Software Challenges for Exascale. EASC 2014. Lecture Notes in Computer Science, (Eds.: S. Markidis E. Laure)*, Springer, Cham, **2015**, pp. 3-27.

[13] S. Pronk, S. Páll, R. Schulz, P. Larsson, P. Bjelkmar, R. Apostolov, M. R. Shirts, J. C. Smith, P. M. Kasson, D. van der Spoel, B. Hess, E. Lindahl, *Bioinformatics* **2013**, *29*, 845-854.

[14] B. Hess, C. Kutzner, D. van der Spoel, E. Lindahl, *J. Chem. Theory Comput.* **2008**, *4*, 435-447.

[15] D. Van Der Spoel, E. Lindahl, B. Hess, G. Groenhof, A. E. Mark, H. J. C. Berendsen, *J. Comput. Chem.* **2005**, *26*, 1701-1718.

[16] E. Lindahl, B. Hess, D. van der Spoel, *Molecular modeling annual* **2001**, *7*, 306-317.

[17] H. J. C. Berendsen, D. van der Spoel, R. van Drunen, *Comput. Phys. Commun.* **1995**, *91*, 43-56.

[18] Lindahl, Abraham, Hess, V. D. Spoel, *GROMACS 2021.7 Source code*, Zenodo, **2023**.

[19] Y.-L. Tsai, P. Cavallo, Q. Lu, J. Yu, C. P. Ender, J. Link, K. Amann-Winkel, K. Endres, C. V. Synatschke, T. John, *Small Sci.* **2025**, *5*, 2500224.

[20] U. Essmann, L. Perera, M. L. Berkowitz, T. Darden, H. Lee, L. G. Pedersen, *J. Chem. Phys.* **1995**, *103*, 8577-8593.

[21] B. Hess, H. Bekker, H. J. C. Berendsen, J. G. E. M. Fraaije, *J. Comput. Chem.* **1997**, *18*, 1463-1472.

[22] B. Hess, *J. Chem. Theory Comput.* **2008**, *4*, 116-122.

[23] S. Miyamoto, P. A. Kollman, *J. Comput. Chem.* **1992**, *13*, 952-962.

[24] G. Bussi, D. Donadio, M. Parrinello, *J. Chem. Phys.* **2007**, *126*.

[25] H. J. C. Berendsen, J. P. M. Postma, W. F. van Gunsteren, A. DiNola, J. R. Haak, *J. Chem. Phys.* **1984**, *81*, 3684-3690.

[26] M. Parrinello, A. Rahman, *J. Appl. Phys.* **1981**, *52*, 7182-7190.

[27] J. Schmidt, C. Ehasz, M. Epperson, K. Klas, J. Wyatt, M. Hennig, M. Forconi, *Org. Biomol. Chem.* **2013**, *11*, 8419.
[28] E. Atherton, H. Fox, D. Harkiss, C. J. Logan, R. C. Sheppard, B. J. Williams, *J. Chem. Soc., Chem. Commun.* **1978**, 537-539.
[29] A. Dong, P. Huang, W. S. Caughey, *Biochemistry* **1990**, *29*, 3303-3308.
[30] U. Rost, C. Steinem, U. Diederichsen, *Chem. Sci.* **2016**, *7*, 5900-5907.
